# C_2_H_5_NO Isomers: From Acetamide to 1,2-Oxazetidine and Beyond

**DOI:** 10.1021/acs.jpca.1c09984

**Published:** 2022-02-03

**Authors:** John M. Simmie

**Affiliations:** School of Chemistry, National University of Ireland, Galway H91 TK33, Ireland

## Abstract

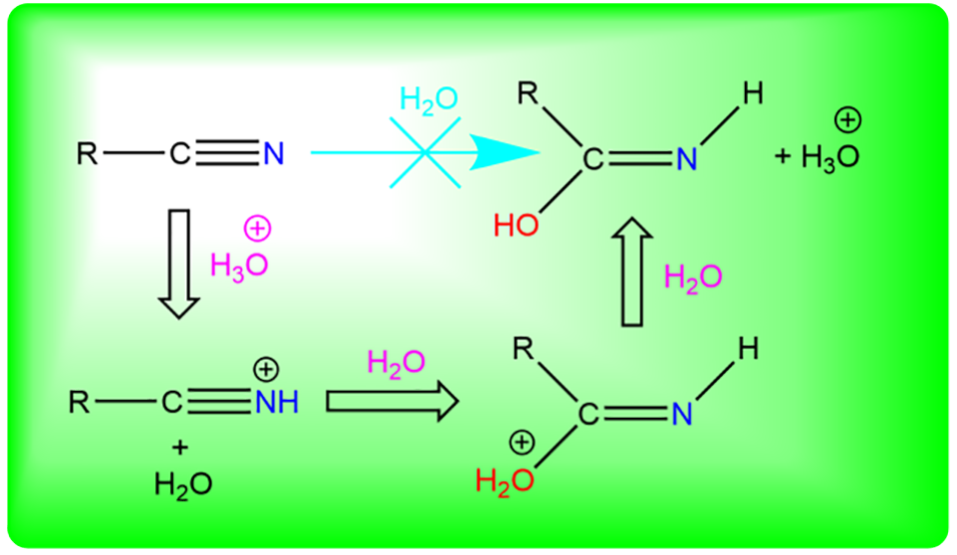

This work documents the properties of a number of isomers of molecular formula C_2_H_5_NO from the most stable, acetamide, through 1,2-oxazetidine and including even higher energy species largely of a dipolar nature. Only two of the isomers have been detected in emissions from the interstellar medium (ISM); possible further candidates are identified, and the likelihood of their being detectable is considered. In general, hardly any of these compounds have been discussed in the existing chemical literature, so this work represents an important contribution extending the canon of chemical bonding which can contribute to machine learning, providing a more exacting test of AI applications. The presence in the ISM of acetamide, CH_3_C(O)NH_2_, is the subject of current debate with no clear and obvious paths to its formation; it is shown that a 1,3-[H]-transfer from (*E,Z*)-ethanimidic acid, CH_3_C(OH)=NH, is feasible in spite of an energy barrier of 130 kJ mol^–1^. It is speculated that imidic acid can itself be formed from abundant precursors, H_2_O and CH_3_C≡N, in an acid-induced, water addition, autocatalytic reaction on water–ice grains. H_3_CC≡NH_3_CC≡NH^+^ + H_2_OH_3_CC(O^+^H_2_)=NHH_3_CC(OH)=NH + H_3_O^+^

## Introduction

The interstellar presence of molecules with a peptide moiety, −C(O)–NH–, is suggestive of an extraterrestrial origin for life on Earth.^1^ Acetamide, H_3_CC(O)NH_2_, is not only one of the most abundant organic molecules present in the neighborhood of Sagittarius B2^2,3^ but has also been found in comets.^4^ It has been postulated to be the key precursor to more complex organic species, but there are currently no obvious routes that would explain its formation in the gas phase.^5,6^ The very recent detection of propionamide, C_2_H_5_CONH_2_, the next member in the homologous series, C_*n*_H_2*n*+1_CONH_2_, suggests that peptide-like molecules might be widespread in space;^7^ however, Kolesniková et al. have not confirmed this claim and did not detect neither propionamide nor the unsaturated prop-2-enamide or acrylamide, H_2_C=CH–CONH_2_, toward Sgr B2(N).^8^ Since nitriles are abundant in the interstellar medium (ISM), Alonso et al. speculated that the hydrolysis of cyanoacetylene, H–C≡C–C≡N, would lead to the formation of 2-propynamide, H–C≡C–CONH_2_; however, the search was unsuccesful.^9^

Apart from *N*-methyl formamide^10^ no other species with molecular formula C_2_H_5_NO has been found, and it is therefore of interest to document those isomers and provide some key background detail of these neutral species which contain C, H, N, and O, the four basic exobiological elements.

For a wide-ranging and authoritative review of prebiotic astrochemistry and the formation of molecules of astrobiological interest in interstellar clouds and protostellar disks, see Sandford et al.^11^ A comprehensive study of peptide-like bond molecules, the GUAPOS project, focused on HNCO, HC(O)NH_2_, CH_3_NCO, CH_3_C(O)NH_2_, CH_3_NHCHO, CH_3_CH_2_NCO, NH_2_C(O)NH_2_, NH_2_C(O)CN, and HOCH_2_C(O)NH_2_ toward the hot core G31.41+0.31, concluding that the first five of these species which were detected were formed on grain surfaces and later released to the gas phase by either thermal or shock-triggered desorption.^12^

Frigge et al. calculated the adiabatic ionization energies of a number of C_2_H_5_NO isomers in a vacuum ultraviolet photoionization study of the formation of *N*-methyl formamide in deep space.^13^ However, there has not been a comparable study to that of Gronowski et al. on the structure and spectroscopy of C_2_HNO isomers for the C_2_H_5_NO isomers.^14^

However, a case study^15^ investigation of the possible routes to the formation of acetamide in the interstellar medium effectively studied its constitutional isomers, creating 198 structures of which 91 were unique. Further refinement led to 53 unimolecular species (none of these unfortunately were available for abstraction by Chemical Abstracts Services) at the G3MP2B3 level of theory from which the authors deduced that the formation of acetamide could involve the bimolecular reactions
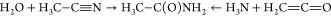
However, they concluded that neither these reactions nor the isomerization of higher energy isomers are likely to be significant contributors to formation of acetamide.

McCarthy and McGuire recently summarized what is known about cyclic molecules in the interstellar medium; their review focuses on C_5_ and C_6_ aromatic species because cyanobenzene had been previously detected.^16^ Subsequently, five-membered rings have been found, 1-cyano-1,3-cyclopentadiene, its highly polar nature μ_*a*_ = 4.15 D no doubt contributing to its discovery, while two isomers of ethynyl cyclopentadiene have been found in the cold prestellar core of the Taurus Molecular Cloud 1.^17,18^

Speculation as to the presence of three-membered rings includes a laboratory study of cyclopropenone^19^ as well as its actual detection,^20^ followed by the first interstellar organic ring, cyclopropenylidene or cyclo-C_3_H_2_, being discovered.^21^ In a laboratory study of the rotational spectrum of furan, Barnum et al. concluded that heterocyclics are peculiarly less abundant than cyclic hydrocarbons in the ISM.^22^

**Figure 1:**
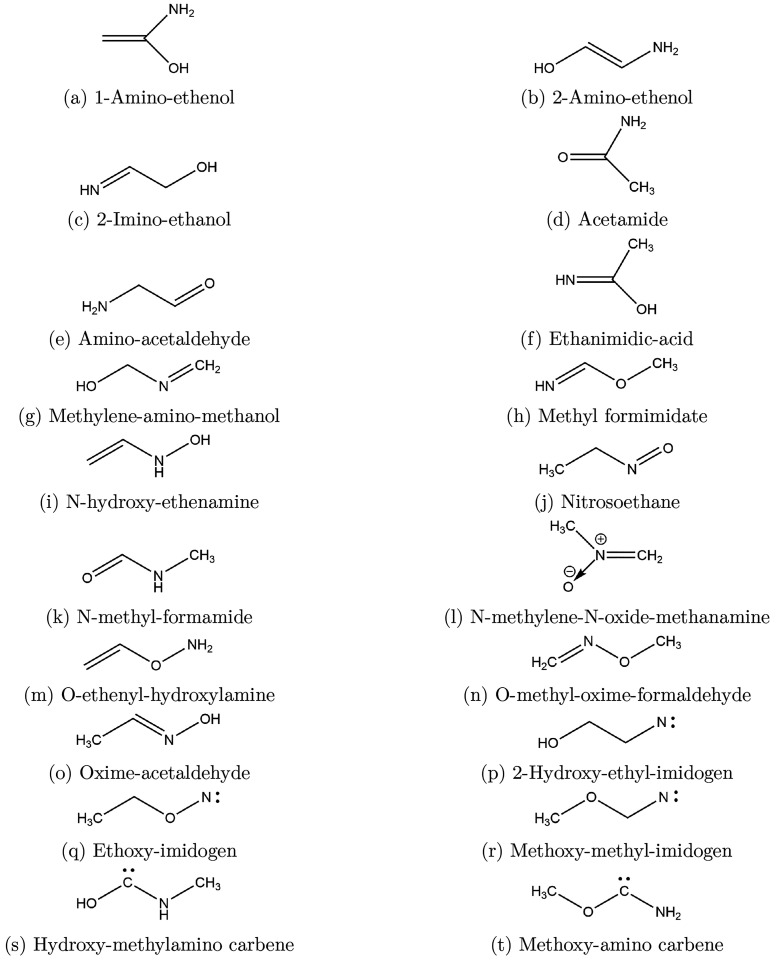
Structures of acyclic species.

**Figure 2:**
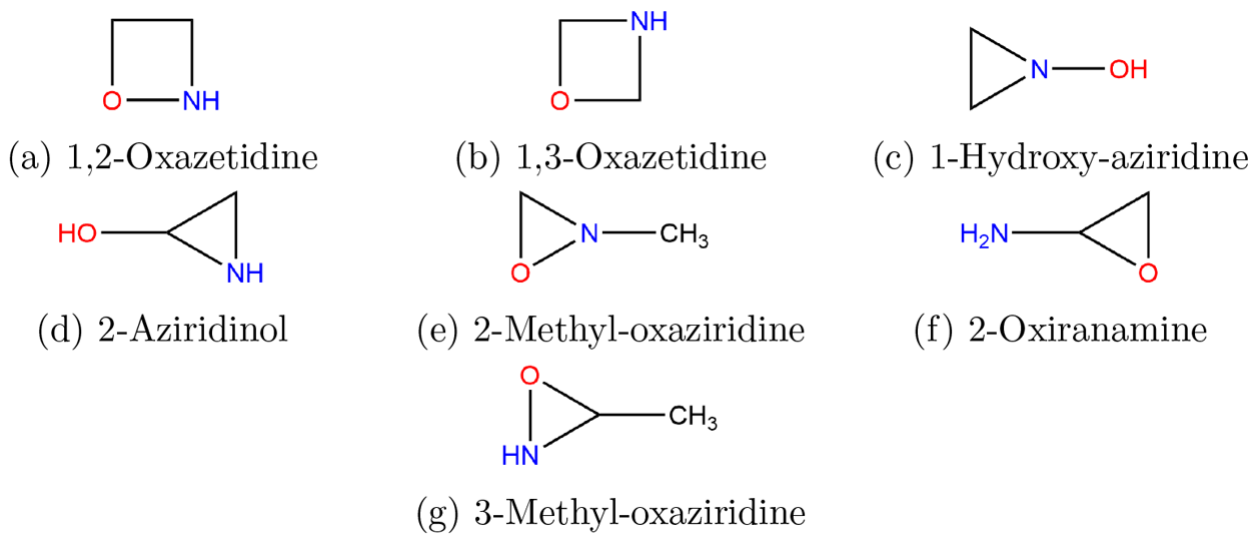
Structures of cyclic species.

A search for already known isomers was conducted here with SciFinder, and those acyclics found are shown in Figure 1, while cyclic species are shown in Figure 2. The objectives of this work are 2-fold: (1) to provide quality data for a series of species, some of which lie outside the normal range of molecules encountered in most chemical databases, which can then be used by machine-learning artificial intelligence procedures as standards or learning sets, and (2) to test whether any of the isomers would be easily detectable in emissions from the ISM. Note that the GDB-17 database^23^ which contains upward of 166 billion organic small molecules with the number of “heavy” atoms ≤ 17 and is comprised of the elements C, N, O, S, and the halogens only lists three such isomers, H_3_C–NH–CHO, H_3_C–CH=N–OH, and H_3_C–O–N=CH_2_ apart from acetamide.^24^

## Theoretical Methods

Preliminary calculations used Spartan’s conformer generation algorithm^25^ at the ωB97X-V/6-311+G(2df,2p) level to determine the lowest lying states; those with an abundance greater than 10%, based on populations, *x*_*i*_, calculated from symmetries, σ_*i*_, and Gibbs free energies, Δ_f_*G*_*i*_^⊖^, and the equation
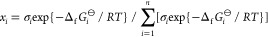
where *R* (J mol^–1^ K^–1^) is the molar gas constant and *T* (K) is the temperature, were retained and reoptimized at B3LYP/cc-pVTZ.

High-level ab initio composite methods were used to compute the atomization energy of each species and hence the formation enthalpies. In principal, Chan and Radom’s W3X-L protocol^26^ was employed, which is based on B3LYP/cc-pVTZ+d geometries and frequencies. The latter are scaled by 0.9886, 0.9926, and 0.9970 to account for the zero-point energy, thermal corrections to the enthalpy, and thermal corrections to the entropy, respectively. Energetics were computed by a combination of coupled-cluster determinations, CCSD(T) and CCSD(T)-F12b, extrapolated to the complete basis set limit (CBS) with aug-cc-pVnZ basis sets up to aug-cc-pVQZ. Core valence correlation and scalar relativistic calculations were performed at the CCSD(T)/cc-pCVTZ level using nonrelativistic frozen-core and all-electron Douglas–Kroll–Hess methods and MP2 and CCSD(T) energies. The above signifies the W2X component of the W3X-L method computed by the Molpro^27,28^ code; the final steps involve post-CCSD(T) effects up to CCSDT(Q) using the multireference application MRCC.^29,30^

In some cases, the computationally less demanding WMS method was used, also centered on B3LYP/cc-pVTZ+d geometries and not on those originally specified by the WMS developers.^31^ It has been recently shown^32^ that this functional, B3LYP, has excellent performance relative to W1-F12//CCSD(T)/CBS. Consequently, W1-F12 energies computed from the functional geometry, which is W-F12//B3LYP/Def2-TZVPP, exhibit a root-mean-square deviation of only 0.29 kJ mol^–1^, and those using a cc-pVTZ+d basis set lead to even better, if unspecified, results. Since only the CHNO species are considered here, cc-pVTZ+d is equivalent to cc-pVTZ.

The WMS composite method can be summarized as folows: (1) achievement of the CCSD(T)/CBS valence correlation energy but via the CCSD(T)-F12b method with only double-ζ and triple-ζ basis sets, (2) parametrization to extrapolate the higher order valence correlation energy from the MP2/CBS, CCSD/CBS, and CCSD(T)/CBS components, and (3) low-cost procedures for inner-shell contributions and scalar relativistic corrections.^31^

Ionization energies were calculated from G4 computations^33^ of the neutral molecule and the associated cation; in addition, formation enthalpies derived from G4 atomization values were obtained.^34^

The applications Gaussian and ChemCraft were employed to carry out the calculations and to view and animate the results.^35,36^ Rate constant calculations were carried out with the Thermo module of MultiWell with scaled vibrational frequencies, rotational constants, and relaxed potential energy scans at the M06-2X/6-311++G(d,p) level but with WMS-derived zero-point-corrected electronic energies.^37−39^

## Results and Discussion

**Figure 3:**
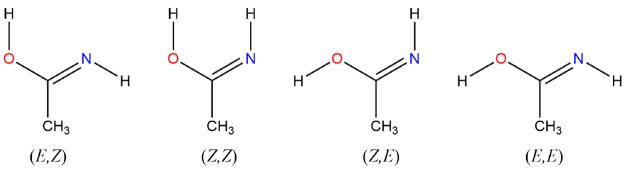
Ethanimidic acid conformers.

**Figure 4:**
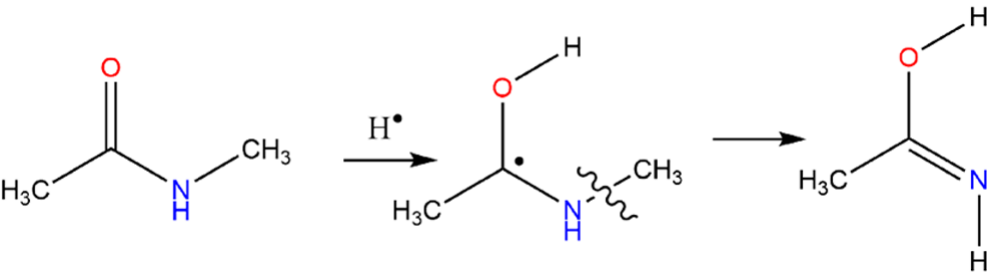
1-Hydroxy-1-(*N*-methyl)aminoethyl radical dissociation.

**Figure 5:**
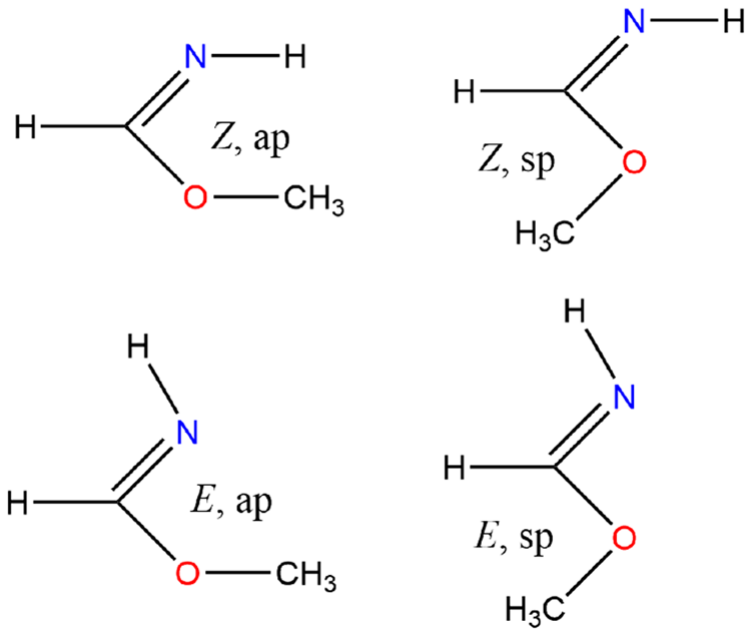
*E* and *Z* stereoisomers and syn and anti periplanar conformations.

**Figure 6:**
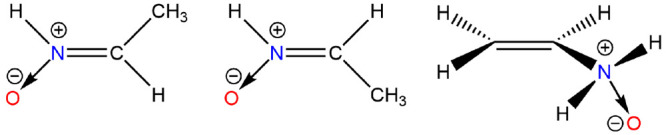
(*E*)- and (*Z*)-*C*-methyl nitrones and vinyl nitrone.

**Figure 7:**
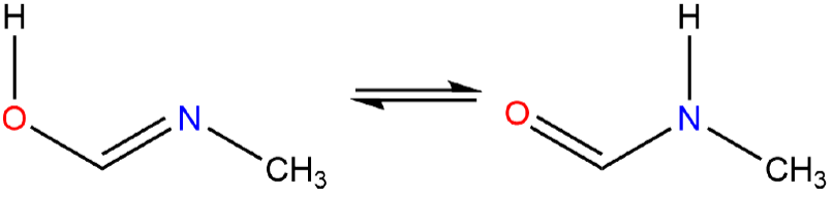
*N*-Methyl-methanimidic acid ⇌ *N*-methyl formamide.

**Figure 8:**
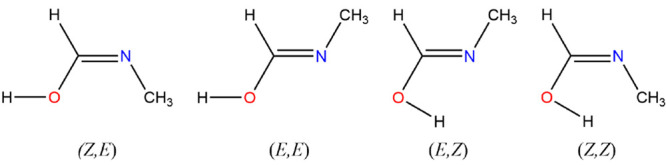
*N*-Methyl formimidic acids.

**Figure 9:**
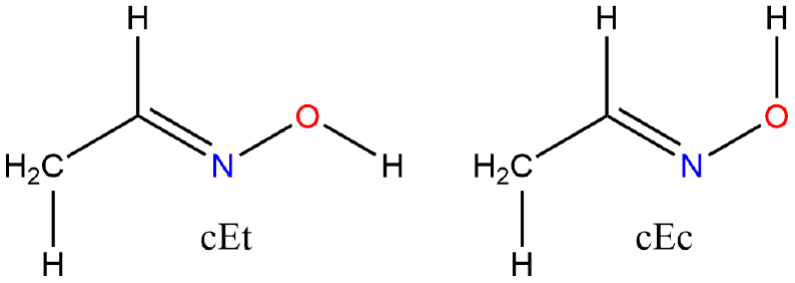
Acetaloximes, *E*.

**Figure 10:**
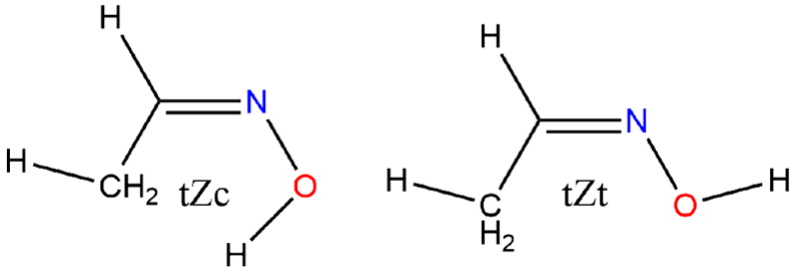
Acetaloximes, *Z*.

**Figure 11:**
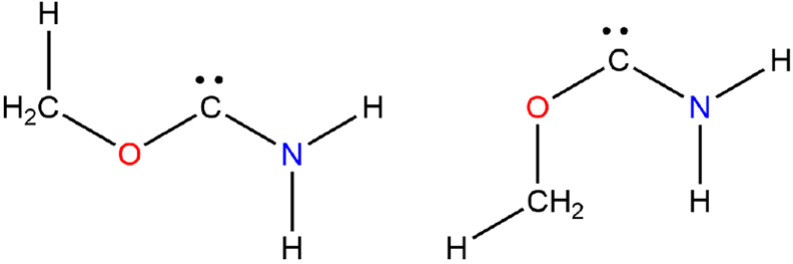
Methoxy(amino) carbenes.

**Figure 12:**
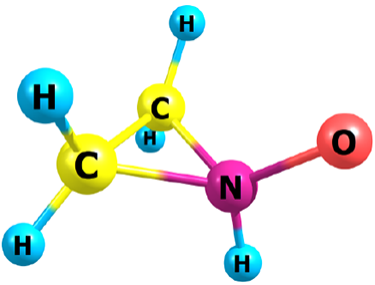
Aziridine *N*-oxide.

**Figure 13:**
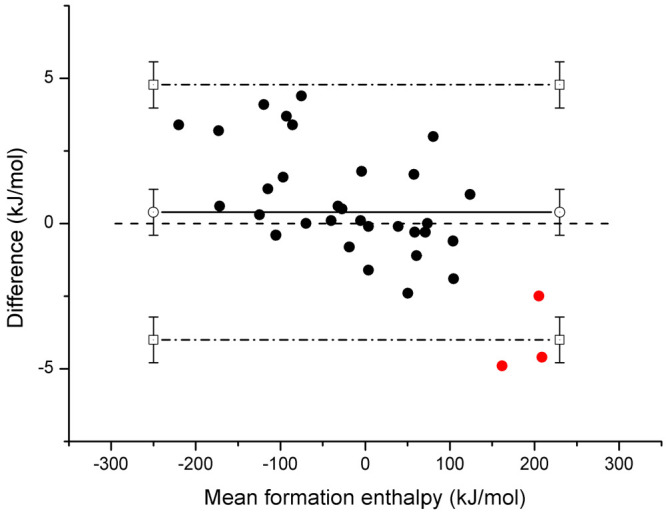
High-level and G4 Δ_f_*H*^⊖^(0 K): bias (solid line), limits of agreement (dotted–dashed line), and nitrenes (red dot).

Computational results for the different categories of molecules under consideration are grouped as follows: first, acyclics, whose structures are shown in Figure 1 and results listed in Table 1, carbenes, whose results are listed separately in Table 2, second, cyclics (Figure 2 and Table 3) and, third, dipolar species with atypical valences. The first two sets of compounds merit discussion on an individual basis as shown in Figures 3–12. An extensive comparison of the properties of those species with the literature is not possible except for adiabatic ionization energies; this comparison is discussed below, centered around Table 4, while Figure 13 compares higher level formation enthalpies with G4-derived values.

**Table 1:** Results for Acyclics (kJ mol^–1^)

species	Δ_f_*H*^⊖^(0 K)	Δ_f_*H*^⊖^(298.15 K)	⟨μ⟩ (D)
1-amino-ethenol	–121.5	–138.3	1.069
2-amino-ethenol *Z*	–94.9	–112.2	2.190
2-amino-ethenol *E*	–77.3	–93.5	1.602
2-imino-ethanol *E*	–87.4	–103.6	3.375
2-imino-ethanol *Z*	–97.6	–115.2	3.073
acetamide	–221.7	–237.1	3.818
amino-acetaldehyde	–115.2	–121.7	1.624
ethanimidic acid *E,Z*	–174.7	–192.1	1.503
methylene-amino-methanol	–69.7	–77.1	1.515
methyl formimidate (*E*, ap)	–104.9	–122.3	0.671
*N*-hydroxy-ethenamine	57.0	40.0	1.320
nitroso-ethane	58.8	42.1	2.471
*N*-methyl-formamide cis	–172.2	–188.6	3.901
*N*-methyl-methanimidic acid	–124.6	–141.7	0.738
*N*-methyl-nitrone	61.5	43.7	3.567
*C*-methyl-nitrone *Z*	37.2	22.3	3.500
*C*-methyl-nitrone *E*	48.3	33.6	4.124
vinyl nitrone	170.9	154.7	4.776
*O*-ethenyl-hydroxylamine	79.0	61.7	1.895
*O*-methyl-oxime-formadehyde *E*	51.8	35.4	0.265
oxime-acetaldehyde *cEt*	–5.6	–22.1	0.725
oxime-acetaldehyde *tZt*	–5.0	–21.3	0.694
carbenes and nitrenes			
amino-hydroxymethyl-carbene ^1^A′	39.3	23.7	2.431
hydroxy-methylamino-carbene ^1^A′	–18.3	–33.6	1.490
methoxy-amino-carbene ^1^A′	4.6	–11.5	1.472
2-hydroxy-ethyl-imidogen ^3^A	164.1	147.9	1.451
ethoxy-imidogen ^3^A″	206.6	190.5	2.927
methoxy-methyl-imidogen ^3^A	211.2	194.9	1.847

**Table 2:** Other Carbenes (kJ mol^–1^)

species	Δ_f_*H*^⊖^(0 K)	Δ_f_*H*^⊖^(298.15 K)	⟨μ⟩ (D)
H_2_N–C̈–CH_2_–OH	39.3	23.7	2.14
H–C̈–NH–CH_2_OH	90.0	74.9	4.13
HO–C̈–CH_2_–NH_2_	116.7	102.2	1.96
H–C̈–O–CH_2_–NH_2_	132.6	117.2	1.43
H_3_C–C̈–NH–OH	165.9	152.3	1.57
H–C̈–N(CH_3_)–OH	182.3	167.1	1.49
H–C̈–O–NH–CH_3_	278.4	263.0	1.99

**Table 3:** Results for Cyclics (kJ mol^–1^)

species	Δ_f_*H*^⊖^(0 K)	Δ_f_*H*^⊖^(298.15 K)	⟨μ⟩ (D)
1,2-oxazetidine	123.7	104.6	2.852
1,3-oxazetidine	4.1	–15.1	1.707
1-hydroxy-aziridine	104.2	86.7	0.502
2-aziridinol trans	–32.2	–50.9	1.702
2-aziridinol cis	–27.5	–40.1	1.009
2-methyl-oxaziridine	105.4	87.2	2.381
2-oxiranamine	–40.1	–58.4	1.058
3-methyl-oxaziridine trans	71.2	53.1	2.426
3-methyl-oxaziridine cis	73.5	55.4	2.771

**Table 4:** G4 Results

		IE (eV)	
species	Δ_f_*H*^⊖^(0 K) (kJ mol^–1^)	calcd	lit.^13^
1-amino-ethenol	–117.4	7.86	7.88
2-amino-ethenol *Z*	–91.2	7.42	7.39
2-amino-ethenol *E*	–82.6	7.61	
2-imino-ethanol *E*	–84.0	9.53	9.51
2-imino-ethanol *Z*	–96.0	9.74	
acetamide	–218.3	9.70	9.74
amino-acetaldehyde	–114.0	9.12	9.12
ethanimidic acid *E,Z*	–171.5	9.72	9.65
methylene-amino-methanol	–69.7	9.39	9.18
methyl formimidate (*E*, ap)	–105.3	9.80	
methyl formimidate (*Z*, sp)	–89.3	9.69	9.71
*N*-hydroxy-ethenamine	58.7	8.14	
nitroso-ethane	58.5	8.91	
*N*-methyl-formamide cis	–171.6	9.79	9.80
*N*-methyl-methanimidic acid	–124.3	9.44	
*N*-methyl-nitrone	60.4	9.02	9.02
*C*-methyl-nitrone *Z*	37.6	8.88	
*C*-methyl-nitrone *E*	49.6	8.16	
*O*-ethenyl-hydroxylamine	82.0	8.83	8.79
*O*-methyl-oxime-formadehyde *E*	49.4	9.49	9.51
oxime-acetaldehyde *cEt*	–5.5	9.76	
oxime-acetaldehyde *tZt*	–3.2	9.55	
carbenes and nitrenes			
amino-hydroxymethyl-carbene ^1^A′	39.2	7.89	
hydroxy-methylamino-carbene ^1^A′	–19.1	8.09	
methoxy-amino-carbene ^1^A′	3.0	8.01	
2-hydroxy-ethyl-imidogen ^3^A	159.2	7.11	
ethoxy-imidogen ^3^A″	204.1	8.16	
methoxy-methyl-imidogen ^3^A″	206.6	9.85	
cyclics			
1,2-oxazetidine	124.7	8.21	
1,3-oxazetidine	4.0	9.00	9.43
1-hydroxy-aziridine	103.6	9.00	
2-aziridinol trans	–31.6		
2-aziridinol cis	–27.0		
2-methyl-oxaziridine	103.5	9.11	
2-oxiranamine	–40.0		
3-methyl-oxaziridine trans	70.9	9.45	
3-methyl-oxaziridine cis	73.5	9.41	9.02

The dipolar species are simply illustrated, much later in the text, in Figure 15, and their basic data are listed in Table 5.

**Table 5:** Dipolar Species (kJ mol^–1^)

	Δ_*f*_*H*^⊖^		
	0 K	298.15 K	⟨μ⟩ (D)
Figure 15a	185.2	169.6	6.46
Figure 15b	177.0	160.8	5.22
Figure 15c	310.8	293.6	2.49
Figure 15d	153.4	138.4	4.31
Figure 15e	421.5	403.9	6.41
Figure 15f	37.6	22.0	3.50
Figure 15g	65.5	50.1	9.14
Figure 15h	328.0	312.6	3.67
Figure 15I	182.3	167.1	1.36
Figure 15j	197.6	181.6	0.81
Figure 15k	141.6	126.7	3.60
Figure 15l	415.4	399.9	3.14
Figure 15m	287.0	272.6	3.29
Figure 15n	522.2	507.6	5.36
Figure 15o	475.8	460.3	4.67
Figure 15p	412.1	397.6	1.83
Figure 15q	195.7	178.0	4.86
Figure 15r	222.3	206.9	4.87
Figure 15s	169.7	153.5	4.78
Figure 15t	80.6	66.2	1.45
Figure 15u	330.0	313.5	4.89

Arising out of the results there is a speculative discussion of the possible routes by which the most stable isomers, acetamide and *N*-methyl formamide, might be formed in the ISM in the section Possible Candidates with the intention of identifying other suitable contenders for detection and their connection to either acetamide or *N*-methyl formamide.

### Alicyclics

#### 1-Amino-ethenol

1-Amino-ethenol has been considered as an intermediate in a quantum chemical study of the ammonolysis of ketene, H_2_C=C=O + NH_3_ → H_2_C=C(NH_2_)OH, which leads ultimately to acetamide.^40^ They place this species at −9.0 kcal mol^–1^ relative to ketene^41^ + ammonia^41^ from CCSD(T)/CBS//MP2/aug-cc-pVTZ calculations, which implies a formation enthalpy of (−45.35 ± 0.12) + (−38.564 ± 0.029) + (−37.66) = −121.6 kJ mol^–1^. This is in excellent agreement with that computed directly here of −121.5 kJ mol^–1^ as indeed are the geometries, cf. C=C 134.2 and 133.9 pm; C=O 136.5 and 136.6 pm; =N 138.5 and 138.4 pm; CCOH −3.3° and +3.3°. A second syn conformer lies 4.5 kJ mol^–1^ above with ∠CCOH = −150.2°.

The compound was recently synthesized via the flash vacuum pyrolysis of malonamic acid and characterized spectroscopically, by trapping in an argon matrix at 10 K, as part of a study of the interstellar presence of prebiotic molecules.^42^ The authors show via coupled cluster computations at the AE-CCSD(T)/cc-pVTZ level of theory reaction energy profiles of 1,3-[H]-transfers linking *anti*-1-amino ethenol and (*Z,E*)-ethanimidic acid, and, *syn*-1-amino ethenol and (*Z,Z*)-ethanimidic acid, Figure 3.

#### 2-Amino-ethenol *Z*

The *Z* conformer is only mentioned once in the literature, where it is postulated as an intermediate in the atmospheric chemical reaction between the solvent monoethanolamine, H_2_NCH_2_CH_2_OH, and the OH radical; that study was prompted by the possible large-scale use of the solvent in postcombustion carbon dioxide capture technologies.^43^

The ground state ^1^A′ of *C*_*s*_ symmetry has ∠HOCC = 0° and ∠CCNH = ± 119.9°; relaxed potential energy scans are compromised by through-space interactions between the OH and NH_2_ groups—it is considerably more stable than the *E* conformer by some 16 kJ mol^–1^.

#### 2-Amino-ethenol *E*

The lowest energy rotamer of the *E* conformers has a CCOH dihedral angle of ca. 0°; scans about the C–O and C–N bonds are well behaved, while the more symmetric *C*_*s*_ state with ∠HOCC = 0.0° and ∠CCNH = ± 120.4° lies 1.7 kJ mol^–1^ higher with Δ_f_*H*^⊖^ (0 K) = −75.6 kJ mol^–1^.

These amino ethenols or enamines are tautomers of the imino ethanols or imines below; Lin et al. showed^44^ that the enamine HOCH=CH–NH_2_ or 2-amino-ethenol *E* lies 18.3 kJ mol^–1^ higher in energy than the corresponding imine, HOCH_2_CH=NH, 2-imino-ethanol *Z*; the corresponding numbers found here, Table 1, are |(−97.6) – (−77.3)| = 20.3 kJ mol^–1^.

#### 2-Imino-ethanol *Z*

The lowest energy conformer can be categorized, based on HNCH/CCOH dihedrals, as (*Z,Z*) with *C*_*s*_ symmetry overall. Rotation about the C–O bond yields the (*Z,E*) conformer, which is at +27.5 kJ mol^–1^.

#### 2-Imino-ethanol *E*

The lowest energy conformer is best described as (∼*E, gauche*) according to NCCO/CCOH dihedrals of −4.3° and −75.1°; the more symmetric *C*_*s*_ state is very close at +0.7 kJ mol^–1^. Two other conformers, variously (*g,g*), lie within +11 kJ mol^–1^.

In a study of the dissociation of amide bonds in peptides, Paizs et al. showed that the neutral *E* imine is 11.7 kJ mol^–1^ more stable (electronic energies uncorrected for ZPEs) than the *Z* imine,^45^ a conclusion which is reinforced here with a zero-point-corrected electronic energy difference of 10.2 kJ mol^–1^.

2-Imino-ethanol, of an unspecific sterochemistry, crops up as an intermediate in flow experiments synthesizing 2-aminooxazole—a key heterocycle leading to nucleotides—from possible prebiotic feedstocks under conditions thought to have existed on an early Earth.^46^

It is also an end product in a G3SX study of the atmospheric chemistry of monoethanolamine, or 2-amino ethanol, which is a widely used solvent for so-called “carbon capture”. Specifically, da Silva uses quantum chemical calculations and master equation kinetic modeling to explore the reaction between the H_2_NĊHCH_2_OH radical and O_2_, in which the imine, 2-amino ethanol, and the hydroperoxyl radical are formed.^47^

#### Acetamide

The most stable of all of the species with molecular formula C_2_H_5_NO, acetamide, is also very well characterized with a formation enthalpy at 298.15 K determined by combustion calorimetry^48^ of −238.33 ± 0.78 kJ mol^–1^. At 0 K, the Thermodynamics Research Centre^49^ recommends −221.0 kJ mol^–1^. The W3X-L results shown in Table 1 are in substantial agreement with Δ_f_*H*^⊖^ = −221.7 kJ mol^–1^ at 0 K and −237.1 kJ mol^–1^ at 298.15 K as indeed is WMS.

It has been detected in the emission and absorption in a star-forming region near the Galactic center together with its parent formamide, HCONH_2_.^50^ The GUAPOS project indicated its presence, outside the Galactic center, in the hot molecular cloud G31 and speculated that acetamide and more generally −C(O)NH– species are prevalent in massive and clustered star-forming regions akin to that in which our own Sun was formed.^12^

The very low barrier to internal rotation of the methyl group of ca. 24 cm^–1^ means that syn, anti, and perpendicular conformations have been found depending on the level of theory and basis set.^51^ Here, the conformation is best declared as syn. The availability of multiple low-lying rotamers makes the rotational spectrum very complex^51^ and has also hindered computations of its thermochemistry such as entropy and heat capacity. Only a very recent determination^52^ exists for the isobaric heat capacity *C*_*P*_^⊖^ = 73.38 and entropy *S*^⊖^ = 274.9 J K^–1^ mol^–1^ at 1 atm and 298.15 K; tests indicate that the results are strongly dependent on the treatment applied with the best values obtained here of *S*^⊖^ = 312.2 and *C*_*P*_^⊖^ = 74.27 J K^–1^ mol^–1^ when the two vibrational modes ν̅_1_ = 27.4 and ν̅_4_ = 503.7 cm^–1^ are replaced by a methyl torsion and a H_2_N–C torsion, respectively, with all other frequencies anharmonics. The H_2_NX “umbrella” mode at ν̅_2_ = 146.3 cm^–1^ remains as a stumbling block to precise evaluation.

An adiabatic ionization energy of 9.71 ± 0.02 eV has been determined by VUV photoionization experiments^53^ using synchrotron and photoelectron/photoion coincidence spectroscopy, in excellent agreement with a G4-computed value of 9.70 eV, Table 4.

#### Amino-acetaldehyde

Balabin showed, from focal-point analysis and ab initio limit computations up to CCSD(T)/CBS, that this keto form is 31.4 ± 1.8 kJ mol^–1^ more stable than the enol form, in this case, 2-amino-ethenol.^54^ The difference found here is |(−115.2) – (−77.3)| = 37.9 kJ mol^–1^, but this is for the most stable conformer of amino-acetaldehyde, whose OCCN dihedral of 0° and CCNH dihedrals of ±57.7° result in *C*_*s*_ symmetry and not the implied structure in the Balabin work (OCCN = −150.0° and CCNH = 79.8° and −160.8°), which is 7.3 kJ mol^–1^*higher* in energy. The directly comparable results are in good agreement, viz. 37.9–7.3 = 30.6 vs 31.4 ± 1.8 kJ mol^–1^.

#### Ethanimidic Acid

The dominant conformer is the (*E,Z*) conformer according to the OCNH/HOCN dihedrals with the (*Z,Z*) conformer at +11.9 kJ mol^–1^, the (*Z,E*) conformer at +14.3 kJ mol^–1^, and the (*E,E*) conformer at +25.3 kJ mol^–1^; in all cases, the molecules exhibit *C*_*s*_ symmetry, Figure 3.

Seasholtz et al. studied the energetics of imino compounds at the G2 level of theory including acetimidic or ethanimidic acid.^55^ They reported Δ_f_*H*^⊖^ = −175.3 and −191.2 kJ mol^–1^ at 0 and 298.15 K, respectively, and a methyl rotor barrier of 5.0 kJ mol^–1^ for “the most stable” but unspecified conformer. WMS-, W2X-, and W3X-L-based values are Δ_f_*H*^⊖^(0 *K*) = −173.1, −174.6, and −174.7 kJ mol^–1^, and at 298.15 K the values are −190.5, −192.0, and −192.1 kJ mol^–1^ as found here for the (*E,Z*) conformer.

Here, the WMS, W2X, and W3X-L 0 K values are −159.1, −160.4, and −160.3 kJ mol^–1^; also, a barrier of 6.8 kJ mol^–1^ is reported for the (*Z,E*) conformer.

The literature value^13^ for the ionization energy of ethanimidic acid is given as 9.65 eV, Table 4, vs a computed value of 9.72 eV for the (*E,Z*) conformer, but the former probably refers to the (*Z,E*) conformer for which we compute a more agreeable 9.62 eV.

Ethanimidic acids appear as intermediates in the dissociation of a radical formed by femtosecond electron transfer to the stable cation formed by O protonation of *N*-methylacetamide. Figure 4 shows the formation of the (*E,Z*) conformer. The objective of the study was to investigate simpler models of the process known as electron capture dissociation with implications for research into medical aspects of aging, radiation damage, and oxidative stress.^56^

#### Methyleneamino-methanol

The lowest conformer, 1, has OCNC and HOCN dihedrals of 1.7° and 79.5°, respectively, 2 has *C*_*s*_ symmetry at +4.0 kJ mol^–1^, and 3 has dihedrals of 130.6° and 47.9° also at +4.0 kJ mol^–1^. The conformer tabulated by Frigge et al. is probably closest to 3, which is described as 165 kJ mol^–1^ less stable than acetamide and with an ionization energy of 9.18 eV.^13^ The comparable values obtained here for the lowest conformer are 149 kJ mol^–1^ and 9.39 eV, clearly not a valid comparison.

During UV photodissociation experiments^57^ conducted at 5 K of the explosive RDX (1,3,5-trinitro-1,3,5-triazinane), mass to charge ratio peaks at 59^+^ were detected by a time-of-flight mass spectrometer during the temperature-programmed desorption phase; this was attributed to the presence of both methylamino methanol, HO–CH_2_–N=CH_2_, and *O*-methyloxime formaldehyde, H_3_C–O–N=CH_2_.

#### Methyl Formimidate

The methyl ester of methanimidic acid or *O*-methyl formimidate exists in *E* and *Z* stereoisomers and anti and syn periplanar conformations of *C*_*s*_ symmetry, Figure 5, as delineated by Lumbroso and Papparlardo in early SCF-MO/4–31G calculations, who found that the (*E*, ap) form is the most stable.^58^

A conclusion which is reinforced by the G4 calculations ranks their Δ_f_*H*^⊖^(0 K) as follows: (*Z*, ap):(*Z*, sp):(*E*, ap):(*E*, sp) = −93.1:–89.3:–105.3:–83.2 kJ mol^–1^. In absolute terms, there is good agreement for (*E*, ap) for which WMS predicts −104.9 kJ mol^–1^ at 0 K. Note that the total dipole moment, ⟨μ⟩ = (μ_*x*_^2^ + μ_*y*_^2^ + μ_*z*_^2^)^1/2^, varies considerably from a low of 0.67 D for the (*E*, ap) conformer to a high of 3.59 D for the (*E*, sp) conformer.

The rotational barriers about the N–C–O–C dihedral are quite high, viz. (*E*, ap) → (*E*, sp) = 44.7 and (*Z*, ap) → (*Z*, sp) = 32.2 kJ mol^–1^, whereas the methyl rotors are typically much lower at 6–10 kJ mol^–1^.

#### *N*-Hydroxy-ethenamine

The ground state has effectively a cis/gauche conformation of CCNO/CNOH dihedrals of −18.4°/116.4°; a change in CNOH to −46.7° results in a conformer at +6 kJ mol^–1^, while a gauche/gauche conformation of dihedrals of 140.0°/124.6° lies 26 kJ mol^–1^ above. Rotation about the C–N bond faces a barrier of 36.6 kJ mol^–1^, while a relaxed potential energy scan about the N–O bond has a barrier of 21.1 kJ mol^–1^ to the next low-lying conformer.

#### Nitroso-ethane

The lowest energy conformer has a cis or syn arrangement with *C*_*s*_ symmetry with two gauche forms, ∠CCNO = ±123.4°, lying very close at ∼2 kJ mol^–1^. The difference between the syn and the anti forms (described as anti but in reality gauche) is slight, ranging from 1 to 3 kJ mol^–1^ according to Fu and co-workers.^59^

Detailed explorations of the microwave spectrum and the potential functions have been carried out by Cox et al.^60,61^ In careful relative intensity measurements they determined a cis/gauche zero-point energy difference of 175 ± 35 cm^–1^ or 2.1 ± 0.4 kJ mol^–1^. This is in excellent agreement with WMS calculations which yield Δ{Δ_f_*H*^⊖^(0 K)|syn – gauche|} = |58.72 – 60.79| = 2.07 kJ mol^–1^.

Relaxed potential energy scans of the methyl and ethyl rotors have barriers of 9.4 and 8.1 kJ mol^–1^, respectively, for the ground state conformer.

#### *N*-Methylene-*N*-oxide-methanamine

The ground state ^1^A′ of *N*-methylene-*N*-oxide-methanamine or *N*-methylnitrone has *C*_*s*_ symmetry; the 3-fold methyl rotor has a barrier of 6.9 kJ mol^–1^. Łukomska et al. discussed the nature of the bonding in this compound and showed that the N–O bond in this acyclic *N*-oxide should be considered as a single dative bond N^⊕^ → O^⊖^ with only a negligible contribution from a double bond.^62^ Furthermore, they showed that although primarily a single bond, the NO bond is significantly shorter at 1.266 Å (here1.262 Å) and stronger at 556 kJ mol^–1^ than other cyclic *N*-oxides. Komaroni et al. argued that the electronic structures of the nitrones could not be represented by one well-defined Lewis-type structure but instead are a mixture of the zwitterionic and the hypervalent structures.^63^

Other nitrones are possible, for example, a *C*-methyl nitrone rather than an *N*-methyl, Figure 6, with *E* and *Z* conformers. The order of stability is *Z* > *E* > H_3_CN(O)=CH_2_, which parallels that for the ethanimines HN=CHCH_3_ and *N*-methylene methanime H_3_CN=CH_2_. Boyd and Boyd carried out theoretical studies of the addition and abstraction by methyl radicals from a series of nitrones,^64^ but, otherwise, they rarely feature in the chemical literature.

Here, both conformers share *C*_*s*_ symmetry and similar N–O bond lengths of 1.263 and 1.266 Å with the *Z*conformer 11.1 kJ mol^–1^ more stable. A significant difference is that the methyl rotor barrier slumps to 1.4 kJ mol^–1^ in the *Z* conformer from 7.0 kJ mol^–1^ for the *E* conformer.

Yet another nitrone is feasible as found by Foo et al.;^15^ this vinyl-nitrone, H_2_C=CHN(→O)H_2_, has a significantly longer N–O bond length at 1.361 Å, not unexpectedly a large dipole moment of ⟨μ⟩ = 4.776 D, and Δ_f_*H*^⊖^ (0 K) = 170.9 ± 2.6 kJ mol^–1^ estimated from multicomposite atomization computations, Figure 6.

#### *N*-Methyl-formamide

On the basis of a molecular line survey at 84.1–114.4 GHz, *N*-methylformamide has been tentatively detected^10^ by Belloche and co-workers as well as more recently and confidently toward Sgr B2(N) and in the star-forming region NGC 63341.^6,65^

Terrestrially, this very well known species has a ground state of ^1^A′ and *C*_*s*_ symmetry. The conformer with cis H’s, technically this is the (*Z*) conformer, is some 5.5 kJ mol^–1^ more stable than that with trans H’s or the (*E*) conformer, in agreement with earlier work^66^ and with a study of nitrogen species by a series of composite methods^67^ where the difference was reported to be 5.38 ± 0.8 kJ mol^–1^ at 298.15 K.

Leach et al. quoted^68^ a value of *ΔH*^⊖^ of −1.938 ± 0.031 eV and referenced the *NIST Chemistry WebBook* as of June 2005, but this link no longer exists; their photoionization mass spectrometric study yielded an adiabatic ionization energy of 9.55 ± 0.04 eV, substantially different from previous determinations. The *WebBook* itself quotes 9.83 ± 0.04 eV, which is in accord with all recent theoretical determinations (viz. this work, 9.79 eV; Frigge et al.,^13^ 9.80 eV).

#### *N*-Methyl-methanimidic Acid

Imidic acids are tautomers of amides and are isomeric to oximes. This particular imidic acid, also known as *N*-methyl formimidimic acid, which currently is not catalogued by SciFinder (Jan 6, 2022), is a tautomer of *N*-methyl-formamide, Figure 7.

An extensive theoretical study of the parent imidic acid HN=C(OH)H was carried out in order to understand the relationship between its tautomers,^69^ while Maier and Endres demonstrated the formation of imidic acid during the photolysis of formamide in an argon matrix with the (*s-Z*)-(*E*) conformer being formed preferentially.^70^ The same applies here where the (*Z,E*) conformer is preferred to the (*E,E*), (*E,Z*), and (*Z,Z*) conformers at +16.9, +18.3, and +20.4 kJ mol^–1^, respectively, where the first refers to the orientation about the CN=CO bond and the second about the NC–OH bond, Figure 8.

Crespo-Otero and co-workers^71^ showed that the (*s,Z*)-(*E*) conformer, which has ∠NCOH = 0°, is 21.6 kJ mol^–1^ more stable than the (*s,E*)-(*E*) conformer, ∠NCOH = 180°.

#### *O*-Ethenyl-hydroxylamine

There are three main conformers to consider; all have *C*_*s*_ symmetry. The lowest has a CCON trans configuration with CONH dihedrals of ±124.9°, while the two have a CCON cis arrangement with ∠CONH = ±124.9° and lie very close in energy at ∼0.1 kJ mol^–1^ or with ±56.4° at +6.1 kJ mol^–1^; there is almost nothing known about this compound.

#### *O*-Methyl-oxime-formadehyde

Both conformers are of *C*_*s*_ symmetry and electronic state ^1^A′ with the *E* value being some 23.9 kJ mol^–1^ more stable than the *Z* value. Relaxed potential energy scans of the methyl group show a typical 3-fold symmetry with a barrier of 19.1 kJ mol^–1^; rotation about O–N faces a barrier of 41.1 kJ mol^–1^ before interconverting to the *Z* conformer at 22.4 kJ mol^–1^.

Kalinowski et al. studied the ozonolysis of *O*-methyloxime as a means to understanding the stability of Crigee intermediates; their starting point is the higher energy *Z* conformer.^72,73^

#### Oxime-acetaldehyde-*E*

Aldoximes have the general formula RHC=NOH and for R = CH_3_ exist in the *E* and *Z* forms. A matrix isolation FTIR and molecular orbital study classified the structures according to rotation about the single bonds C–C and N–O as cis or trans about HCCN and CNOH: *cEc* and *cEt* and *tZc* and *tZt*. The global minimum corresponds to *cEt* with the *tZt* form only 2.6 kJ mol^–1^ higher.^74^

A number of composite methods, G2, G3, G3B3, and G3MP2B3, was used to calculate the enthalpies of formation of substituted hydroxylamines and oximes^75^ including acetaloxime with Δ_f_*H*^⊖^ (298.15 K) = −21.9 → −22.5 kJ mol^–1^, which agrees with an earlier determination^76^ of −22.55 ± 0.29 kJ mol^–1^; the corresponding value found here is −22.1 kJ mol^–1^, Table 1.

Hosoi and co-workers observed the microwave spectra of six isotopic species of (*E*)- and (*Z*)-acetaldehyde oximes or acetaloximes.^77^ For the *E* conformer they determined rotational constants of 45 453 ± 560, 4237.665 ± 21, and 3973.807 ± 21 MHz and an average barrier height for the methyl rotor of 7.88 ± 0.20 kJ mol^–1^, Figure 9. The comparable numbers calculated here of *A*_*e*_ = 46 620, *B*_*e*_ = 4238, and *C*_*e*_ = 3980 MHz are probably uncertain to the extent of ±1%.^78^

#### Oxime-acetaldehyde-*Z*

For the *Z* conformer, Hosoi et al. determined rotational constants of 17 215 ± 18, 6626.48 ± 40, and 4920.70 ± 34 MHz and an average barrier height for the methyl rotor of 1.65 ± 0.08 kJ mol^–1^, Figure 10. They attributed the drastically lower barrier to steric repulsion between the methyl and the hydroxyl groups, a conclusion with which we concur as the methyl rotor barrier slumps from 8.1 to 0.2 kJ mol^–1^.

Here, rotational constants (MHz) of *A*_*e*_ = 17 520 ± 170, *B*_*e*_ = 6680 ± 75, and *C*_*e*_ = 4983 ± 45 are computed from B3LYP/B2PLYP/M06-2X/ωB97XD/PBE0DH calculations all at cc-pVTZ. Note that for both the *E* and the *Z* stereoisomers, Hosoi et al. did not directly measure either the rotational constant *A* or the moment of inertia, *I*_*a*_ = *h*/(8π^2^*cA*), but instead estimated the latter from the approximate relation *I*_*a*_ ≈ *I*_*c*_ – *I*_*b*_.

### Carbenes

#### Hydroxy(methylamino) Carbene

The existence of hydroxy(methylamino) carbene was demonstrated by gas-phase experiments in which one-electron reduction of radical cations was followed by neutralization–reionization.^79^ Thus, H_3_C–N=C(H)–OH, *N*-methyl-methanimidic acid, and H_3_C–NH–C̈–OH were established.

The lowest state corresponds to a trans/trans arrangement for HOCN and OCNC dihedrals with a cis/trans structure at +19.7 kJ mol^–1^; both singlet states are of *C*_*s*_ symmetry; the triplet is at a very much higher energy.

Very recently, a *trans*-aminohydroxymethylene carbene was synthesized, H_2_N–C̈–OH, by pyrolysis of oxalic acid monoamide and trapping in solid argon.^80^ IR spectra at 3 K together with a computed anharmonic spectrum at B3LYP/6-311++G(3df,3pd) enabled the identification. Geometrically, ∠OC̈N = 107.8°, *d*(O–C̈) = 1.347 Å, and *d*(C̈–N) = 1.322 Å; these compare well with the values for hydroxy(methylamino), viz. 107.7°, 1.356 Å, and 1.319 Å.

#### Methoxy(amino) Carbene

In agreement with Alkorta and Elguero,^81^ the singlet is considerably more stable than the triplet carbene, at the G4 level by 311.9 kJ mol^–1^; an index name for this species, H_2_N–C̈–O–CH_3_, is as yet unassigned by SciFinder (Jan 6, 2022).

The lowest conformer has a trans COCN structure with the cis conformer at +28.9 kJ mol^–1^; both have *C*_*s*_ symmetry, Figure 11.

#### Other Carbenes

A number of variants are feasible, all of which are considerably less stable than the two carbenes considered above but still more stable than cyclopropenylidene or indeed methylene. These higher energy isomers, some of which are derived from the structural work of Foo and colleagues,^15^ are ranked in order of increasing formation enthalpy in Table 2.

### Nitrenes

#### 2-Hydroxy-ethyl-imidogen

The lowest triplet state has HOCC and OCCN dihedrals of −64.2° and +66.9°, respectively, with *C*_*s*_ symmetry at +4.5 kJ mol^–1^. In a computational study of the reaction of triplet nitrenes with oxygen, Liu et al. found^82^ that 2-hydroxy-ethyl imidogen, HOCH_2_CH_2_^3^N, is some 41.4 kJ mol^–1^ more stable than ethoxy imidogen, CH_3_CH_2_O^3^N. This is in good agreement with the value computed here of (206.6 – 164.1) = 42.5 kJ mol^–1^, Table 1.

#### Ethoxy-imidogen

The triplet state is considerably more stable than the singlet state, in agreement with earlier work of the Hadad group;^82^ the ground state has a NOCC dihedral of 180° and *C*_*s*_ symmetry and is accompanied by two close-lying conformers with NOCC dihedrals of ±75°, generated by relaxed PE scans with barriers of 3.7 kJ mol^–1^, whereas the equivalent scan for the singlet state is accompanied by dissociation.

#### Methoxy-methyl-imidogen

MNDO investigations of the 1,2-rearrangement of singlet carbenes and nitrenes by Frenking and Schmidt included CH_3_OCH_2_–N̈ → CH_3_OCH=NH.^83^

The ground triplet state is not the obvious trans state of *C*_*s*_ symmetry but a gauche ∠COCN = −72.3°, which lies 5.3 kJ mol^–1^ lower.

### Cyclics

#### 1,2-Oxazetidine

In a study of conventional ring strain energies in oxadiazetidines, Benton and Magers showed that 1,2-oxazetidine is much less strained than all six systems examined in spite of the fact that its total electronic energy is some 121–16 kJ mol^–1^ higher than that of 1,3-oxazetidine.^84^ Galván and co-workers showed that in contrast to 1,2-dioxetane, 1,2-oxazetidine cannot undergo chemiexcitation and subsequent chemiluminescence.^85^

#### 1,3-Oxazetidine

As alluded to above, this isomer of *C*_*s*_ symmetry and state ^1^A′ is 119.6 kJ mol^–1^ more stable than the 1,2-oxazetidine, Table 3. It is noticeably less “buckled” than the 1,2 isomer.

Even a comprehensive treatise on heterocyclic chemistry which deals specifically with four-membered rings with one oxygen and one nitrogen atom has remarkably little to say about oxazetidines.^86^

#### 1-Hydroxy-aziridine

This symmetric system, *C*_*s*_ and ^1^A′, exists in two forms with the 180° form some 22.25 kJ mol^–1^ more stable than the 0° form at the G4 level where the dihedral angle is defined by H–O–N to the C–C midpoint. A relaxed potential energy scan faces a barrier of 23.6 kJ mol^–1^, leading to the 0° conformer at 22.0 kJ mol^–1^.

#### 2-Aziridinol

This chiral molecule, 2-hydroxyaziridine, is mainly, 72.6%, in the trans form with opposed OH and NH groups and ∠NCOH = 8.5°, the cis or the same side groups lie at +4.5 kJ mol^–1^ with ∠NCOH = −88.7° and contribute 18.6%, while a further cis conformer is at +7.2 kJ mol^–1^ or 4.4% and differs from the previous one by a NCOH dihedral of +156.8°.

The high-symmetry, *C*_*s*_ and ^1^A′, nitrone-type molecule^15^ shown in Figure 12 (∠ONH = 116.4°, *d*(O–N) = 1.318 Å, and *d*(N–H) = 1.024 Å) has a large dipole moment, ⟨μ⟩ = 4.869 D, but also a very large formation enthalpy of 195.7 kJ mol^–1^ (178.0 at 298.15 K).

#### 2-Methyl-oxaziridine

This *N*-methyl-substituted oxaziridine has a ground state with an unusually long O–N bond^87^ of 1.498 Å; the methyl rotor barrier of 12.4 kJ mol^–1^ is unexceptional.

Apart from two articles concerned with the calculation of the optical rotation for this chiral molecule, there is little else in the literature.^88,89^

#### 2-Oxiranamine

The lowest energy conformer corresponds to OCNH dihedrals of +80°/–41°, while scans about the C–N bond indicate two others at +12.6 kJ mol^–1^ with ∠OCNH = +96°/–143° and +12.3 kJ mol^–1^ with −56°/–179°.

In an interesting article, Ellinger and others addressed the question of chirality in the interstellar medium.^90^ Arguing from the fact that currently the only chiral species detected is propylene oxide, c-(H_2_COCH)–CH_3_, they adduced that successful detection requires the following:

- a “rigid molecule”, leading to a rotational spectrum of least complexity,
- a significant dipole moment, probably on the order of 2 D,
- a high abundance, possible targets should either be the most stable isomer or at least sufficiently close in energy with a suggested upper limit of ∼125 kJ mol^–1^,
- a weak adsorption on icy surfaces, allowing the molecule to “fly free” and therefore become detectable.

Their discussion considers aminooxirane, or 2-oxiranamine, which is chiral, and they show it lies 200 kJ mol^–1^ above acetamide, has a dipole moment of 1.0 D, and has an adsorption energy on water ice of 64.4 kJ mol^–1^. Although none of these values by themselves render 2-oxiranamine undetectable, they makes it unlikely, unless of course precursor species are present in high abundance. Here, it is found that 2-oxiranamine lies 182 kJ mol^–1^ above acetamide and has a dipole moment of 1.06 D, Tables 1–3.

Complex formation between a single water molecule and 2-oxiranamine is stabilized, according to M06-2X/aug-cc-pVTZ counterpoise calculations, by −34.3 → −36.7 kJ mol^–1^, depending upon the exact structure of the complex; this must be compared to values of −36.0 → −46.3 kJ mol^–1^ for acetamide–water complexes. The latter values agree with the PBE+GD3/aug-cc-pVTZ results by Krestyaninov et al.^91^ While not directly comparable to the interactions between molecules and grains of water–ice in the interstellar medium, it does suggest that 2-oxiranamine is somewhat more likely to “fly freely”.

In a theoretical study^92^ of the reaction between CH_3_C^•^HNH_2_ and O_2_, a calculated CBS-QB3 atomization energy of −6.1 kJ mol^–1^ is given; in conjunction with Δ_f_*H*^⊖^(0 K) = +37.248 kJ mol^–1^ from ATcT,^41^ a formation enthalpy for 2-oxiranamine of −43.4 kJ mol^–1^ can be derived. This this compares not unfavorably with the higher level result of −40.1 kJ mol^–1^ computed here, Table 3.

#### 3-Methyl-oxaziridine

3-Methyl-oxaziridine is postulated as a potential product in a kinetic study of the CH_3_C^•^HNH_2_ + O_2_ reaction,^92^ while Taghizadev et al. investigated the structures of a number of methyl derivatives of oxaziridines.^93^

This chiral system exists mainly (71.2%) as the trans form with the NH and methyl groups on opposite sides of the ring with the cis or same-side conformer at +2.25 kJ mol^–1^ (28.8%).

### Ionization Energies

Adiabatic ionization energies were computed with the composite method G4, IE (eV) = 27.2116 × {*G*4(0 K)[cation] – *G*4(0 K)[neutral]}, which has been shown to perform adequately.^94^ There is good agreement with the results of the Kaiser group^13^ except for the cyclics 1,3-oxazetidine and 3-methyl-oxaziridine which have been mistabulated^95^ as 9.43 and 9.02 eV, respectively, instead of 9.02 and 9.43 eV, see Table 4, and for methylene-amino-methanol which can be rationalized because here the lowest energy conformer with ∠CNCO = 0° is considered as opposed to what is probably a gauche conformer.

There are very few experimental measurements against which these results can be compared. The electron/ion coincidence spectroscopic data of Schwell et al. yielded 9.71 eV for acetamide^53^ vs a computed 9.70 eV, but for *N*-methyl formamide the calculated value of 9.79 eV is wildly at odds with the 9.55 eV value obtained in a photoionization mass spectrometric study by Leach and co-workers.^68^

In addition, atomization energies and consequently formation enthalpies at 0 K were tabulated, Table 4, and compared to the higher level results in Tables 1–3 by means of a Bland–Altman^96^ plot, Figure 13. The bias, which is the average deviation of G4 from the higher level methods, is a modest 0.39 kJ mol^–1^. The most obvious deviations are shown by the nitrenes in their triplet state, Figure 13, which is not totally unexpected.

### Dipolar Species

Additional calculations were carried out for a number of mainly dipolar species derived from the work of Foo et al.;^15^ these are shown in Figure 15 and cross-referenced to Table 5. These molecules are characterized by large dipole moments, unsurprisingly, with the species in Figure 15g outstanding at ⟨μ⟩ = 9.14 D.

Of the five cyclic compounds present, none of them appear to be likely candidates for detection since they do not fit the Ellinger criteria.^90^ Although there has been a tentative detection of aziridine^97^ in hot cores around young stars, the aziridine *N*-oxide, Figure 15q, does have an enhanced dipole moment vis-à-vis aziridine, which would make it more liable for detection since it retains the high symmetry and rigid structure of its parent. However, apart probably from the nitrone, Figure 15f, which corresponds to *ZC*-methyl nitrone discussed earlier and whose values are listed in Table 1, *energetically* these compounds are unlikely to be of relevance based on our current understanding of conditions in the ISM.

**Figure 14:**
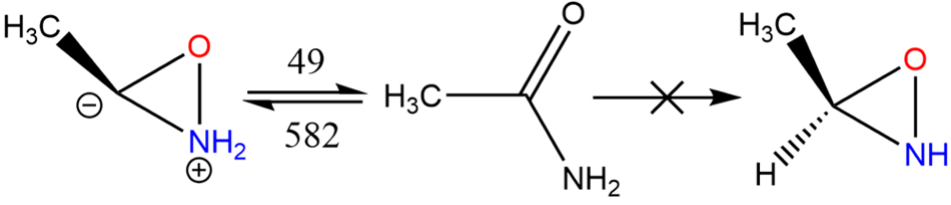
Ring closure (kJ mol^–1^).

**Figure 15:**
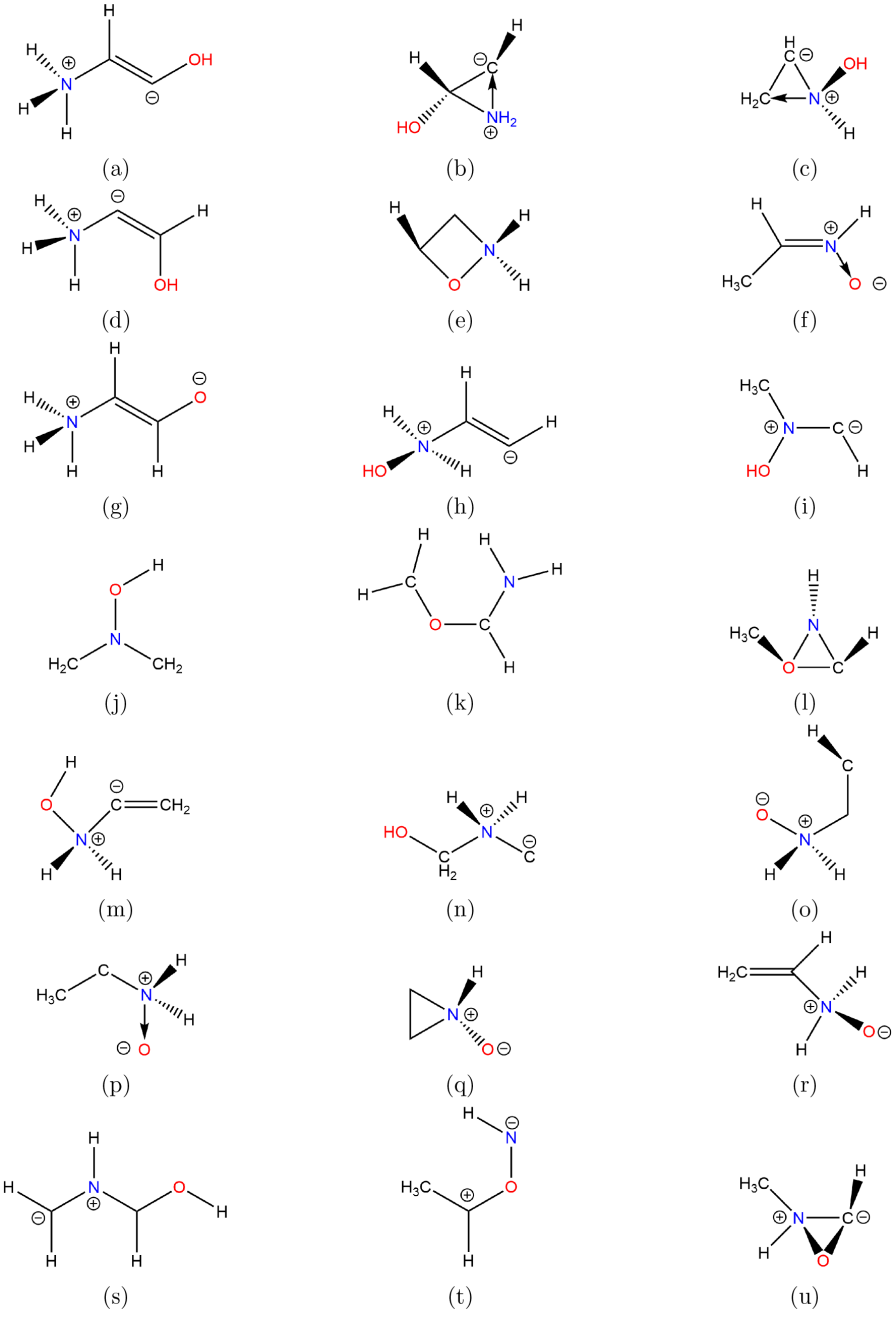
Structures of dipolar species.

An indication of the complexity of this system can be gained from the fact that an attempt to carry out a ring closure reaction from acetamide, in the expectation that 3-methyl oxaziridine would be formed, proved illusory, Figure 14. Yet another dipolar compound is formed whose formation enthalpy of 312.4 kJ mol^–1^ (296.1 at 298.15 K) and dipole moment of 3.88 D makes it akin to the species in Figure 15c. A similar attempt to ring-close *N*-methyl formamide resulted in the structure in Figure 15u, for which Δ_f_*H*^⊖^(0 K) = 330.0 kJ mol^–1^ (313.5 at 298.15 K) and ⟨μ⟩ = 4.89 D; clearly this system exhibits quite a diversity of chemical bonding.

### Possible Candidates

If the criteria outlined by Ellinger et al. are decisive in determing whether a particular species is detectable or not then it is unlikely that any of the chiral cyclic molecules featured here will qualify.^90^

More generally, are any of the acyclics likely candidates? If only those species which lie within an arbitrary 100 kJ mol^–1^ are worthy of consideration then 1-amino ethenol, amino acetaldehyde, ethanimidic acid, methyl formimidate, and *N*-methyl methanimidic acid qualify. However, all of these have dipole moments toward the lower end of the scale. Another consideration that can impact the detectability of these compounds is the number of low-lying conformers, which effectively reduces the population and hence the intensity of a particular rotational transition.

**Figure 16:**
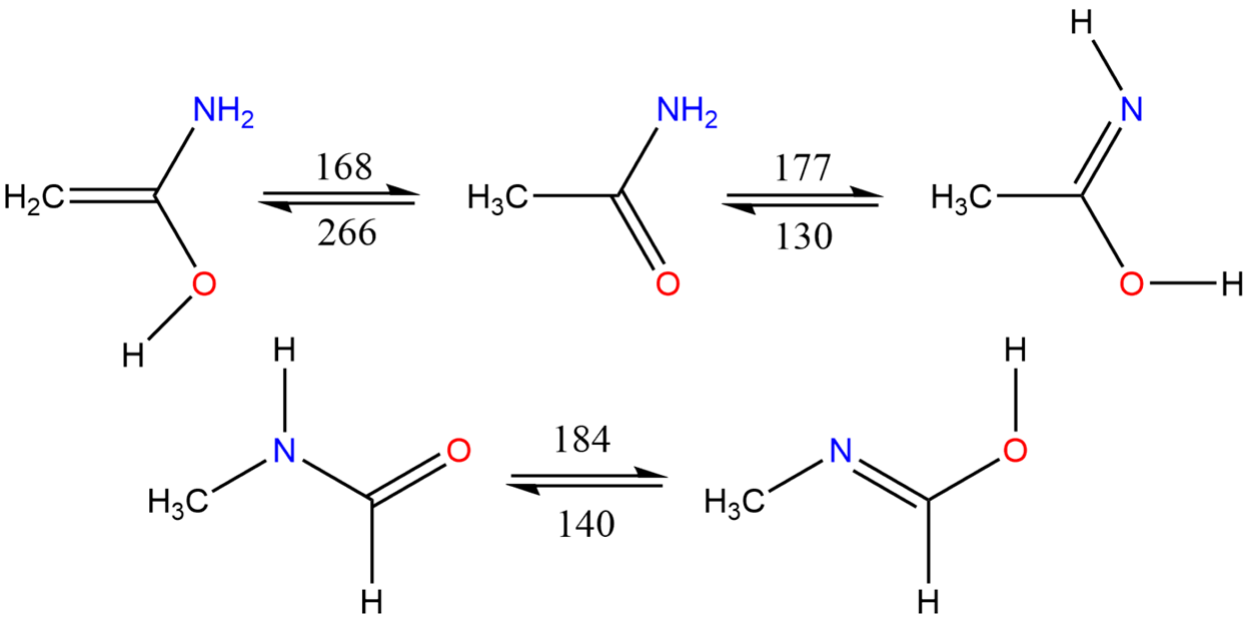
H-Transfer reactions: forward and reverse barriers (kJ mol^–1^).

In the absence of comprehensive chemical kinetic mechanisms for the formation of species in the interstellar medium, including the key compound acetamide, it is difficult to propose routes to the candidate C_2_H_5_NO molecules. Given that acetamide is present in high abundance, it is not unreasonable to look at this as a possible source; then, it can be noted that simple H-atom transfer reactions, Figure 16, link acetamide to 1-amino ethenol and (*E,Z*)-ethanimidic acid, Figure 3, while the reasonably abundant (*Z*) rotamer of *N*-methyl formamide is linked to (*E,Z*)-*N*-methyl formimidic acid, Figure 8.

Some studies have explored the opposite approach, that is the formation of higher energy isomers from which lower energy species would emerge;^42,98^ so, for example, Foo et al. considered bimolecular (not of interest here) and unimolecular routes to acetamide—the latter from *N*-methyl formamide, acetimidic (ethanimidic) acid, 1-amino ethenol, and 2-amino acetaldehyde.^15^ They show that the energy barrier for (*E,Z*)-acetimidic acid → acetamide is 119 kJ mol^–1^ (this work, 130 kJ mol^–1^), while the barrier for *anti*-1-amino ethenol → acetamide is 170 kJ mol^–1^; the latter agrees well with values of 164 and 168 kJ mol^–1^ from Mardyukov^42^ and this work, respectively.

While it is true that a barrier of 168 kJ mol^–1^ renders the process H_2_C=C(OH)NH_2_ → H_3_C–C(O)NH_2_ unfeasible thermally, tunnelling plays an increasing role at temperatures less than 300 K. Thus, for the 1,3-[H]-transfer reaction from (*E,Z*)-ethanimidic acid to acetamide, an approximate rate constant of *k* ≈ 1.6 × 10^–11^ s^–1^ at 100 K can be calculated based on M06-2X/6-311++G(d,p)-scaled frequencies and relaxed potential energy scans for the methyl and OH hindered rotors in the reactant and just the methyl rotor in the transition state. Eckart tunnelling is included based on *iω̅* = 1973 cm^–1^, WMS-computed zero-point electronic energies for the reactant, transition state, and product of −174.2, −44.5, and −221.5 kJ mol^–1^, respectively, a forward barrier of 129.7 kJ mol^–1^, and a reverse barrier of 177.0 kJ mol^–1^.^37^ A derived half-life of 1400 years probably means that such a reaction could possibly play a role, provided of course that there are independent routes to ethanimidic acid.

Given that species with a C≡N bond are abundant (27 such have been detected^99^), one might speculate that acid-induced addition of H_2_O could provide a feasible channel
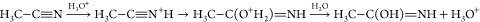
presumably on water–ice grains. It is known experimentally that H_3_O^+^ exists as protonic defects in the lattice as a result of UV photolysis of ice, and as stated by Moon et al., “H_3_O^+^ may have a substantial population in interstellar ice in UV-irradiating environments and participate in acid–base reactions in the solid phase” as well as being detected in the ISM.^100,101^

In a recent review article, Lee and Kang discussed the intricacies of proton transport in ice and distinguished between the highly mobile proton in the interior which hops along a chain of water molecules and protons trapped on the surface.^102^ They concluded that “spontaneous acid–base reactions may occur under interstellar ice conditions, even without external energy input. Excess protons may be generated by the photolysis of ice particles under ionizing radiation or by the injection of cosmic protons into the ice. The excess protons can be stored as hydronium ion in the ice and utilized for subsequent chemical reaction...”.

The reaction between H–C≡N and H_2_O to form first methanimidic acid, HC(OH)=NH, and then formamide, HC(O)–NH_2_, has been shown by Rimola et al. not to be competitive, despite the cooperation of additional water molecules, due to a high energy barrier in theoretical calculations of this reaction on a 33-H_2_O ice cluster model.^103^ This is view was reinforced by Darla et al. in ωB97xD/aug-ccpVTZ gas-phase calculations in which they showed that the presence of an additional water molecule neither as a participant nor as a spectator makes a sufficient reduction in the barriers to reaction to render the process kinetically significant.^104^

Woon showed in density functional calculations of a cluster of 24 water molecules that C^+^(^2^P) reacts with H–C≡N to form a transient species H–C=N–C^+^ first, which then reacts with a neighboring H_2_O to form H–C(OH_2_)=N–C^+^, which then loses a proton from the O atom to a water molecule forming H–C(OH)=NC + H_3_O^+^; “the entire process has no activation barriers whatsoever”.^105^

Under severe conditions^106^ the methanimidic acid can be further protonated at the N atom, CH_3_C(OH)=N^+^H_2_ ↔ CH_3_C(O^+^H)–NH_2_, and then deprotonated at O to yield the amide directly, CH_3_C(O)NH_2_, and indeed even further to a carboxylic acid, CH_3_C(O)OH, but it is unclear whether such severe conditions would apply in the ISM.

Direct confirmation of this speculation, that is, that the acid-induced water addition to methyl cyanide might be difficult to obtain since ethanimidic acid is probably less easily detectable (rotational and centrifugal distortion constants and harmonic and anharmonic frequencies are shown in the Supporting Information, Tables S6 and S7) due to its much lower dipole moment in comparison to acetamide, although the not unrelated ethanimime, CH_3_–CH=NH, has been found in a survey of Sagittarius B2 North in both the (*Z*) and the (*E*) conformations.^107^ Note that a counterpoise M06-2X/aug-cc-pVTZ calculation shows that (*E,Z*)-ethanimidic acid is somewhat more strongly bound (−52.8 kJ mol^–1^) than acetamide (−36.0 → −46.3 kJ mol^–1^) by a single water molecule—an indication that it might be less likely to fly freely.

Bulak et al. carried out UV photolysis of water-rich ices with H_3_C–C≡N at 20 K and using laser desorption postionization time-of-flight mass spectrometry found the prompt appearance of *m*/*e* peaks at 59^+^ (and at 61^+^ with ^18^O) from an irradiated 20:1 water:methyl cyanide mixture.^108^ They concluded that acetamide/*N*-methyl formamide is formed and deduced that the O atom and OH radical addition followed by hydrogenation represent viable pathways to the products; however, product identification was nonspecific, and alternative explanations are feasible.

**Figure 17:**
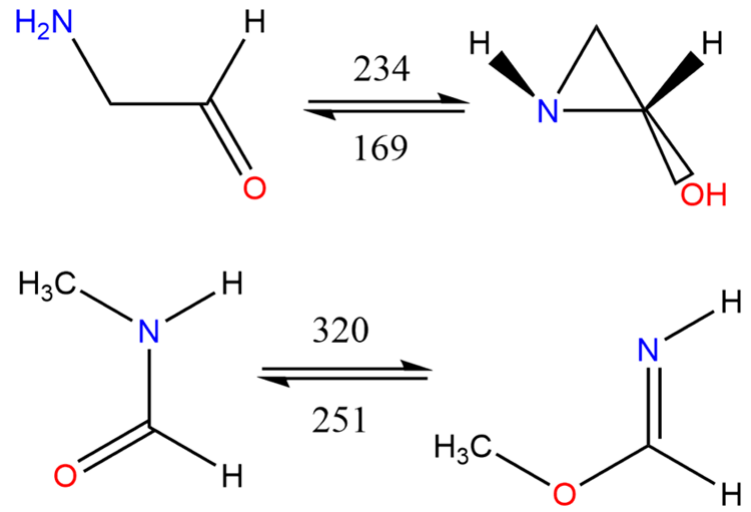
Speculative reactions: forward and reverse barriers (kJ mol^–1^).

There are no obvious routes from or to 2-amino acetaldehyde to acetamide or indeed from/to *N*-methyl formamide, but 2-amino acetaldehyde does connect rather surprisingly to *trans*-2-aziridinol as does (*E*,ap)-methyl formimidate with *N*-methyl formamide with a 1,3-[CH_3_]-transfer, Figure 17, but these are unlikely to be of any real importance in the absence of a significant tunnelling contribution.

## Conclusions

High-level ab initio atomization energy calculations have been carried out to rank a number of “known” C_2_H_5_NO neutral molecules from the most stable, acetamide, to the least stable, methoxy methyl imidogen. In addition, dipole moments and adiabatic ionization energies are reported and compared to the literature, what little of it exists. Higher energy species incorporating more diverse types of bonding than the traditional tetravalent carbon, trivalent nitrogen, and divalent oxygen are also documented.

An alternative scheme is outlined to explain acetamide formation routes in the ISM; kinetically, tunnelling in a 1,3-[H]-transfer reaction from a higher energy isomer, ethanimidic acid, seems to offer a possible channel which is probably more reasonable than the current examples^2,3,109^ in the literature
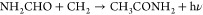

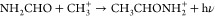

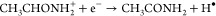

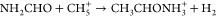

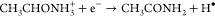

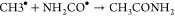


A general mechanism is then needed to generate this imidic acid from more abundant precursors, and it is proposed that acid-induced water addition to carbon–nitrogen triple bonds, hosted on water–ice grains, not the gas-phase, of , meets this requirement. The autocatalytic nature of this reaction is a point in its favor as well as the fact that the more direct addition  has been discounted.^103,104^

Since this system exhibits quite a range of bonding, even within the restrictive context of neutral and closed-shell molecules, the results are likely to be useful for incorporation into databases for artificial intelligence learning/predictive efforts, ameliorating the well-known problem of data set bias.

## Supporting Information Available

Cartesian coordinates, rotational constants, frequencies, and energetics of species discussed (PDF)
