# Supplementary material for: C2H5NO Isomers: From Acetamide to 1,2-Oxazetidine and Beyond
Source: J Phys Chem A. 2022 Feb 3;126(6):924–39. doi: 10.1021/acs.jpca.1c09984 (PMC8859852; doi:10.1021/acs.jpca.1c09984)
Supplement: Supplementary file 1 — jp1c09984_si_001.pdf [file jp1c09984_si_001.pdf]

Supporting Information for

C_2H_5NO Isomers: from Acetamide to 1,2-Oxazetidine and Beyond

Author: John M. Simmie

john.simmie@nuigalway.ie

School of Chemistry, National University of Ireland

Galway, Ireland H91 TK33

Appendix

Tables S6 and S7 contain spectroscopic data for (E,Z) ethanimidic acid at B3LYP/cc-pVTZ.

Table S6: Rotational and centrifugal distortion constants.
See Watson, J. K. G., *J. Chem. Phys.* **1967**, *46*, 1935–1949; ibid. **1966**, *45*, 1360-1361.

Parameter	A	Parameter	S	Units
Δ_J	8.23	D_J	9.36	/ kHz
Δ_K	−0.628	D_{JK}	−13.7	/ kHz
Δ_{JK}	−6.83	D_K	5.07	/ kHz
δ_J	0.403	d_1	−0.403	/ kHz
δ_K	14.4	d_2	0.570	/ kHz
Φ_J	0.0102	H_J	0.0170	/ Hz
Φ_K	−0.581	H_K	−0.0104	/ Hz
Φ_{JK}	0.238	H_{JK}	−0.0558	/ Hz
Φ_{KJ}	−0.829	H_{KJ}	0.04914	/ Hz
ϕ_J	0.000378	h_1	0.00288	/ Hz
ϕ_K	−1.37	h_2	−0.00339	/ Hz
ϕ_{JK}	0.129	h_3	−0.00251	/ Hz
$A_e = 10,886, B_e = 9,327.8, C_e = 5183.5$ / GHz				
$A_0 = 10,800, B_0 = 9,258.1, C_0 = 5138.0$ / GHz				
At CCSD(T)-F12b/aVTZ:				
$A_e = 10,893, B_e = 9,337.1, C_e = 5,188.8$ / GHz				

Table S7: Harmonic, $\bar{\nu}$, anharmonic frequencies, ω, and symmetries, σ / cm^{-1}

$\bar{\nu}$	ω	σ	$\bar{\nu}$	ω	σ	$\bar{\nu}$	ω	σ
3743.92	3555.09	a'	1390.83	1354.76	a'	3099.10	2959.37	a''
3519.96	3351.82	a'	1261.35	1248.16	a'	1474.65	1429.17	a''
3135.21	2993.77	a'	1102.46	1070.58	a'	1072.70	1047.36	a'
3044.00	2945.03	a''	1016.28	993.95	a'	844.65	822.11	a''
1738.26	1710.55	a'	868.52	849.97	a''	631.33	609.00	a''
1486.30	1448.79	a'	552.22	547.70	a'	525.21	514.75	a'
1437.55	1382.24	a''	425.94	426.61	a'	132.08	114.98	a''

#1 1-amino-ethenol

Charge = 0 Multiplicity = 1 Stoichiometry C2H5NO

OPTIMIZED GEOMETRY

Center Number	Atomic Number	Atomic Type	Coordinates (Angstroms) X	Y	Z
1	6	0	-0.098496	-0.000636	0.003809
2	6	0	-1.401390	-0.278992	0.007973
3	7	0	0.949461	-0.926260	-0.081140
4	8	0	0.347858	1.290565	0.047666
5	1	0	-1.748741	-1.296192	0.097672
6	1	0	-2.125624	0.514648	-0.075791
7	1	0	1.626073	-0.829060	0.666100
8	1	0	0.630137	-1.881171	-0.147386
9	1	0	1.188381	1.328843	-0.424640

A, B, C / GHz 10.4676840 9.8787368 5.1622496

FREQUENCIES AND ROTATIONAL CONSTANTS

INDEX NO.	DOF TYPE	CM-1(UNSCALED)	CM-1(SCALED BY)	SYMMETRY	IR-INTENSITY
1	vib	322.49	0.00	A	22.8881
2	vib	450.45	0.00	A	106.8977
3	vib	457.13	0.00	A	21.1293
4	vib	502.88	0.00	A	26.1824
5	vib	584.42	0.00	A	72.0742
6	vib	681.22	0.00	A	42.1468
7	vib	782.81	0.00	A	77.5178
8	vib	799.90	0.00	A	146.1423
9	vib	920.80	0.00	A	27.7564
10	vib	981.63	0.00	A	23.0046
11	vib	1150.46	0.00	A	18.4855
12	vib	1242.28	0.00	A	61.3117
13	vib	1366.82	0.00	A	183.2122
14	vib	1440.02	0.00	A	3.1579
15	vib	1635.49	0.00	A	45.7097
16	vib	1747.15	0.00	A	215.9462
17	vib	3173.27	0.00	A	1.1042
18	vib	3263.93	0.00	A	5.3890
19	vib	3505.60	0.00	A	3.6441
20	vib	3616.78	0.00	A	10.1448
21	vib	3774.52	0.00	A	37.7459
22	rot	0.3491644	-		
23	rot	0.3295192	-		
24	rot	0.1721941	-		

```
--------------------------------------------------------------------------------

--------------------------------------------------------------------------------
------------------------ ZPE and THERMAL CONTRIBUTIONS -------------------------
--------------------------------------------------------------------------------
Eelectronic                    [kJ/mol]    = -549416.559
Ezpe                           [kJ/mol]    = +193.796
Eelectronic+Ezpe               [kJ/mol]    = -549222.763

Eelectronic                    [hartrees] = -209.261687
Ezpe                           [hartrees] = +0.073813
Eelectronic+Ezpe               [hartrees] = -209.187874

Thermal Correction to Energy   [kJ/mol]    = +205.776
Thermal Correction to Enthalpy [kJ/mol]    = +208.255
Thermal Correction to Gibbs    [kJ/mol]    = +124.457
--------------------------------------------------------------------------------
```

#10 Figure 15a

Charge = 0 Multiplicity = 1 Stoichiometry   C2H5NO

```
---------------------------------------------------------------------
---------------------- OPTIMIZED GEOMETRY --------------------------
---------------------------------------------------------------------
```

Center Number	Atomic Number	Atomic Type	Coordinates (Angstroms) X	Y	Z
1	6	0	-0.843495	-0.859227	-0.323829
2	6	0	0.220830	-0.195805	0.396868
3	7	0	-0.905815	0.701885	-0.062732
4	8	0	1.440366	-0.008326	-0.241810
5	1	0	-1.563327	-1.308059	0.360981
6	1	0	0.314280	-0.162231	1.486427
7	1	0	-0.733485	1.268252	-0.879344
8	1	0	-1.469874	1.147302	0.651472
9	1	0	2.006182	0.538336	0.315842

A, B, C / GHz   17.4809641   7.1388508   5.9929899

```
--------------------------------------------------------------------------------
-------------------- FREQUENCIES AND ROTATIONAL CONSTANTS ---------------------
--------------------------------------------------------------------------------
```

INDEX NO.	DOF TYPE	CM-1(UNSCALED)	CM-1(SCALED BY)	SYMMETRY	IR-INTENSITY
1	vib	378.64	0.00	A	6.5531
2	vib	423.17	0.00	A	94.2689
3	vib	492.02	0.00	A	6.6798
4	vib	560.06	0.00	A	1.6772

5	vib	748.84	0.00	A	7.4922
6	vib	820.14	0.00	A	67.3257
7	vib	848.48	0.00	A	36.8659
8	vib	914.22	0.00	A	1.1653
9	vib	943.49	0.00	A	199.7825
10	vib	1076.30	0.00	A	115.0154
11	vib	1111.89	0.00	A	9.8776
12	vib	1228.42	0.00	A	3.0498
13	vib	1252.57	0.00	A	112.2166
14	vib	1328.46	0.00	A	37.0050
15	vib	1441.39	0.00	A	32.2993
16	vib	1603.14	0.00	A	23.2838
17	vib	2997.52	0.00	A	97.6250
18	vib	3075.12	0.00	A	51.5406
19	vib	3471.11	0.00	A	8.4229
20	vib	3606.36	0.00	A	41.3069
21	vib	3774.71	0.00	A	10.2099
22	rot	0.5831022	-		
23	rot	0.2381264	-		
24	rot	0.1999046	-		

```
--------------------------------------------------------------------------------

--------------------------------------------------------------------------------
----------------------- ZPE and THERMAL CONTRIBUTIONS ------------------------
--------------------------------------------------------------------------------
Eelectronic                     [kJ/mol]   = -549117.166
Ezpe                            [kJ/mol]   = +191.977
Eelectronic+Ezpe                [kJ/mol]   = -548925.189

Eelectronic                     [hartrees] = -209.147654
Ezpe                            [hartrees] = +0.073120
Eelectronic+Ezpe                [hartrees] = -209.074534

Thermal Correction to Energy    [kJ/mol]   = +203.248
Thermal Correction to Enthalpy  [kJ/mol]   = +205.726
Thermal Correction to Gibbs     [kJ/mol]   = +123.441
--------------------------------------------------------------------------------
```

#100 methyl formimidate

Charge = 0 Multiplicity = 1 Stoichiometry C2H5NO

```
---------------------------------------------------------------------
---------------------- OPTIMIZED GEOMETRY --------------------------
---------------------------------------------------------------------
Center     Atomic      Atomic             Coordinates (Angstroms)
Number     Number       Type             X           Y           Z
---------------------------------------------------------------------
```

1	6	0	-0.783408	0.488306	0.000000
2	6	0	1.380787	-0.443150	-0.000000
3	7	0	-1.292877	-0.666913	0.000000
4	8	0	0.537020	0.716794	-0.000000
5	1	0	2.398289	-0.063728	-0.000001
6	1	0	1.196007	-1.050848	-0.884813
7	1	0	1.196008	-1.050847	0.884814
8	1	0	-2.306149	-0.612143	-0.000000
9	1	0	-1.314455	1.440671	-0.000000

A, B, C / GHz 19.6545885 6.6674288 5.1383084

FREQUENCIES AND ROTATIONAL CONSTANTS

INDEX NO.	DOF TYPE	CM-1(UNSCALED)	CM-1(SCALED BY)	SYMMETRY	IR-INTENSITY
1	vib	169.43	0.00	A	0.0111
2	vib	306.17	0.00	A	49.1396
3	vib	315.73	0.00	A	15.0746
4	vib	724.56	0.00	A	5.6492
5	vib	831.80	0.00	A	38.9329
6	vib	926.19	0.00	A	53.7011
7	vib	1042.82	0.00	A	1.1242
8	vib	1077.36	0.00	A	144.9285
9	vib	1176.08	0.00	A	1.1308
10	vib	1216.71	0.00	A	26.3922
11	vib	1303.77	0.00	A	125.1285
12	vib	1400.36	0.00	A	24.2800
13	vib	1469.04	0.00	A	8.8960
14	vib	1480.68	0.00	A	9.0281
15	vib	1500.94	0.00	A	11.2284
16	vib	1704.85	0.00	A	228.2464
17	vib	3044.23	0.00	A	31.4146
18	vib	3085.39	0.00	A	38.4930
19	vib	3110.48	0.00	A	22.5721
20	vib	3145.82	0.00	A	18.1015
21	vib	3521.64	0.00	A	5.1660
22	rot	0.6556065	-		
23	rot	0.2224015	-		
24	rot	0.1713955	-		

ZPE and THERMAL CONTRIBUTIONS

Eelectronic [kJ/mol] = -549412.653

Ezpe [kJ/mol] = +194.718

Eelectronic+Ezpe [kJ/mol] = -549217.935

Eelectronic [hartrees] = -209.260199

```
Ezpe                              [hartrees] = +0.074164
Eelectronic+Ezpe                  [hartrees] = -209.186035

Thermal Correction to Energy   [kJ/mol]   = +206.811
Thermal Correction to Enthalpy [kJ/mol]   = +209.292
Thermal Correction to Gibbs    [kJ/mol]   = +124.598
--------------------------------------------------------------------------------
```

#11 Figure 15c

Charge = 0 Multiplicity = 1 Stoichiometry C2H5NO

OPTIMIZED GEOMETRY

Center Number	Atomic Number	Atomic Type	Coordinates (Angstroms) X	Y	Z
1	6	0	0.801331	0.830041	-0.313137
2	6	0	1.000255	-0.646338	-0.148602
3	7	0	-0.158352	0.041907	0.439166
4	8	0	-1.474224	-0.122497	-0.110812
5	1	0	1.382107	1.455512	0.360233
6	1	0	0.747167	-1.304756	-0.971837
7	1	0	1.646128	-1.106870	0.600322
8	1	0	-0.268817	0.034405	1.447329
9	1	0	-1.413842	0.506111	-0.853284

A, B, C / GHz 18.4378574 7.1491507 6.2362163

FREQUENCIES AND ROTATIONAL CONSTANTS

INDEX NO.	DOF TYPE	CM-1(UNSCALED)	CM-1(SCALED BY)	SYMMETRY	IR-INTENSITY
1	vib	378.66	0.00	A	17.3513
2	vib	419.13	0.00	A	34.8569
3	vib	468.08	0.00	A	73.5050
4	vib	711.44	0.00	A	104.2360
5	vib	739.61	0.00	A	27.2633
6	vib	842.48	0.00	A	12.9437
7	vib	901.65	0.00	A	17.5883
8	vib	909.69	0.00	A	26.9837
9	vib	1087.72	0.00	A	40.6640
10	vib	1102.81	0.00	A	7.2579
11	vib	1137.60	0.00	A	6.9932
12	vib	1212.08	0.00	A	12.7329
13	vib	1234.41	0.00	A	0.9451
14	vib	1370.52	0.00	A	39.1199

15	vib	1469.00	0.00	A	39.1816
16	vib	1514.67	0.00	A	2.0352
17	vib	3032.87	0.00	A	80.0570
18	vib	3100.81	0.00	A	25.4395
19	vib	3138.21	0.00	A	17.3608
20	vib	3485.79	0.00	A	11.5323
21	vib	3620.32	0.00	A	43.6435
22	rot	0.6150207	-		
23	rot	0.2384700	-		
24	rot	0.2080178	-		

```
---------------------------------------------------------------------------------
------------------------ ZPE and THERMAL CONTRIBUTIONS ------------------------
---------------------------------------------------------------------------------
Eelectronic                    [kJ/mol]    = -548987.878
Ezpe                           [kJ/mol]    = +190.669
Eelectronic+Ezpe               [kJ/mol]    = -548797.209

Eelectronic                    [hartrees] = -209.098411
Ezpe                           [hartrees] = +0.072622
Eelectronic+Ezpe               [hartrees] = -209.025789

Thermal Correction to Energy   [kJ/mol]    = +201.707
Thermal Correction to Enthalpy [kJ/mol]    = +204.185
Thermal Correction to Gibbs    [kJ/mol]    = +122.327
---------------------------------------------------------------------------------
```

#111 Figure 15r

Charge = 0 Multiplicity = 1 Stoichiometry   C2H5NO

OPTIMIZED GEOMETRY

Center Number	Atomic Number	Atomic Type	Coordinates (Angstroms) X	Y	Z
1	6	0	0.601314	-0.382304	0.278004
2	6	0	1.726511	0.089878	-0.223849
3	7	0	-0.642826	0.389628	0.286519
4	8	0	-1.620678	-0.254546	-0.406442
5	1	0	0.440586	-1.384422	0.643201
6	1	0	2.611791	-0.526646	-0.274764
7	1	0	1.800690	1.096315	-0.616459
8	1	0	-0.437393	1.324279	-0.099624
9	1	0	-0.917413	0.553995	1.268624

```
A, B, C / GHz         27.7340126          4.7366673          4.5999598

--------------------------------------------------------------------------------
-------------------- FREQUENCIES AND ROTATIONAL CONSTANTS ----------------------
--------------------------------------------------------------------------------
INDEX NO. DOF TYPE  CM-1(UNSCALED)  CM-1(SCALED BY ) SYMMETRY IR-INTENSITY
   1        vib          135.37          0.00             A        22.0712
   2        vib          348.31          0.00             A        45.9921
   3        vib          443.83          0.00             A         4.4302
   4        vib          665.26          0.00             A        10.0557
   5        vib          903.35          0.00             A         5.6812
   6        vib          935.50          0.00             A         3.5221
   7        vib          944.54          0.00             A       102.6400
   8        vib          983.41          0.00             A         6.5381
   9        vib         1001.32          0.00             A         6.2500
  10        vib         1163.60          0.00             A         6.6664
  11        vib         1273.86          0.00             A         4.1904
  12        vib         1310.11          0.00             A         0.9378
  13        vib         1390.97          0.00             A        17.4523
  14        vib         1444.52          0.00             A         6.9676
  15        vib         1645.55          0.00             A        16.9625
  16        vib         1710.33          0.00             A        31.7243
  17        vib         3143.01          0.00             A         2.1298
  18        vib         3191.09          0.00             A        34.8990
  19        vib         3212.40          0.00             A        11.0872
  20        vib         3224.20          0.00             A         4.1093
  21        vib         3239.79          0.00             A         2.7204
  22        rot       0.9251071          -
  23        rot       0.1579982          -
  24        rot       0.1534381          -
--------------------------------------------------------------------------------

--------------------------------------------------------------------------------
------------------------ ZPE and THERMAL CONTRIBUTIONS -------------------------
--------------------------------------------------------------------------------
Eelectronic                    [kJ/mol]   = -549127.791
Ezpe                           [kJ/mol]   = +193.258
Eelectronic+Ezpe               [kJ/mol]   = -548934.533

Eelectronic                    [hartrees] = -209.151701
Ezpe                           [hartrees] = +0.073608
Eelectronic+Ezpe               [hartrees] = -209.078093

Thermal Correction to Energy   [kJ/mol]   = +205.191
Thermal Correction to Enthalpy [kJ/mol]   = +207.669
Thermal Correction to Gibbs    [kJ/mol]   = +123.013
--------------------------------------------------------------------------------
```

#118 Z,E ethanimidic acid

Charge = 0 Multiplicity = 1 Stoichiometry C2H5NO

OPTIMIZED GEOMETRY

Center Number	Atomic Number	Atomic Type	X	Y	Z
1	6	0	-0.117617	-0.104509	0.000000
2	6	0	1.380143	-0.194380	-0.000000
3	7	0	-0.858549	-1.123639	0.000000
4	8	0	-0.634042	1.166854	-0.000000
5	1	0	1.683680	-1.236135	-0.000000
6	1	0	1.794625	0.298522	0.882712
7	1	0	1.794625	0.298522	-0.882712
8	1	0	-1.841894	-0.854209	0.000000
9	1	0	0.075982	1.817274	0.000002

Coordinates (Angstroms)

A, B, C / GHz 10.6804968 9.3604650 5.1481232

FREQUENCIES AND ROTATIONAL CONSTANTS

INDEX NO.	DOF TYPE	CM-1(UNSCALED)	CM-1(SCALED BY)	SYMMETRY	IR-INTENSITY
1	vib	166.71	0.00	A	0.1372
2	vib	374.64	0.00	A	107.5016
3	vib	427.26	0.00	A	3.0209
4	vib	542.31	0.00	A	26.5921
5	vib	543.86	0.00	A	11.6583
6	vib	853.72	0.00	A	40.7383
7	vib	915.98	0.00	A	64.4434
8	vib	1007.12	0.00	A	5.6625
9	vib	1077.52	0.00	A	0.7500
10	vib	1116.43	0.00	A	44.2791
11	vib	1229.20	0.00	A	50.6291
12	vib	1354.95	0.00	A	265.1785
13	vib	1415.44	0.00	A	70.3546
14	vib	1478.43	0.00	A	1.9424
15	vib	1488.56	0.00	A	7.8695
16	vib	1771.98	0.00	A	165.4072
17	vib	3026.09	0.00	A	13.1582
18	vib	3074.06	0.00	A	13.5538
19	vib	3152.93	0.00	A	3.8625
20	vib	3462.95	0.00	A	2.7823
21	vib	3805.86	0.00	A	40.2240
22	rot	0.3562630	-		
23	rot	0.3122315	-		
24	rot	0.1717229	-		

ZPE and THERMAL CONTRIBUTIONS

Eelectronic	[kJ/mol]	=	-549458.685
Ezpe	[kJ/mol]	=	+193.113
Eelectronic+Ezpe	[kJ/mol]	=	-549265.572
Eelectronic	[hartrees]	=	-209.277732
Ezpe	[hartrees]	=	+0.073553
Eelectronic+Ezpe	[hartrees]	=	-209.204179
Thermal Correction to Energy	[kJ/mol]	=	+205.451
Thermal Correction to Enthalpy	[kJ/mol]	=	+207.929
Thermal Correction to Gibbs	[kJ/mol]	=	+122.808

#119 tZt oxime-acetaldehyde

Charge = 0 Multiplicity = 1 Stoichiometry C2H5NO

OPTIMIZED GEOMETRY

Center Number	Atomic Number	Atomic Type	Coordinates (Angstroms) X	Y	Z
1	6	0	-0.521186	0.704092	-0.000001
2	6	0	-1.418567	-0.492619	-0.000000
3	7	0	0.749582	0.696472	-0.000000
4	8	0	1.255814	-0.618245	0.000001
5	1	0	-0.945857	1.701687	-0.000001
6	1	0	-1.225398	-1.115946	-0.874961
7	1	0	-2.464883	-0.195695	-0.000007
8	1	0	-1.225408	-1.115937	0.874969
9	1	0	2.206481	-0.472290	0.000001

A, B, C / GHz 17.4449931 6.5814875 4.9222861

FREQUENCIES AND ROTATIONAL CONSTANTS

INDEX NO.	DOF TYPE	CM-1(UNSCALED)	CM-1(SCALED BY)	SYMMETRY	IR-INTENSITY
1	vib	53.15	0.00	A	0.0104
2	vib	284.06	0.00	A	1.0438
3	vib	403.41	0.00	A	76.1342
4	vib	524.81	0.00	A	36.4897

5	vib	686.05	0.00	A	15.8436
6	vib	845.66	0.00	A	17.4078
7	vib	912.18	0.00	A	79.4415
8	vib	964.96	0.00	A	38.0493
9	vib	1058.14	0.00	A	0.2519
10	vib	1115.71	0.00	A	19.4621
11	vib	1337.70	0.00	A	36.7319
12	vib	1372.16	0.00	A	41.5928
13	vib	1409.80	0.00	A	13.7547
14	vib	1477.54	0.00	A	9.5018
15	vib	1483.10	0.00	A	8.5371
16	vib	1727.06	0.00	A	5.5489
17	vib	3034.91	0.00	A	9.1719
18	vib	3082.02	0.00	A	9.2816
19	vib	3121.49	0.00	A	13.3239
20	vib	3155.85	0.00	A	11.7398
21	vib	3825.56	0.00	A	90.1905
22	rot	0.5819023	-		
23	rot	0.2195348	-		
24	rot	0.1641898	-		

```
--------------------------------------------------------------------------------

--------------------------------------------------------------------------------
------------------------ ZPE and THERMAL CONTRIBUTIONS -------------------------
--------------------------------------------------------------------------------
Eelectronic                    [kJ/mol]   = -549306.422
Ezpe                           [kJ/mol]   = +190.656
Eelectronic+Ezpe               [kJ/mol]   = -549115.766

Eelectronic                    [hartrees] = -209.219738
Ezpe                           [hartrees] = +0.072617
Eelectronic+Ezpe               [hartrees] = -209.147121

Thermal Correction to Energy   [kJ/mol]   = +203.739
Thermal Correction to Enthalpy [kJ/mol]   = +206.217
Thermal Correction to Gibbs    [kJ/mol]   = +117.998
--------------------------------------------------------------------------------
```

#120 E,E N-methyl methanimidic acid

```
Charge = 0 Multiplicity = 1  Stoichiometry    C2H5NO

---------------------------------------------------------------------
---------------------- OPTIMIZED GEOMETRY ---------------------------
---------------------------------------------------------------------
Center     Atomic      Atomic             Coordinates (Angstroms)
Number     Number       Type             X           Y           Z
---------------------------------------------------------------------
```

1	6	0	-0.522801	0.295367	-0.000000
2	6	0	1.783561	0.084199	0.000001
3	7	0	0.453769	-0.485679	-0.000002
4	8	0	-1.792928	-0.183956	0.000002
5	1	0	-0.445058	1.390477	0.000001
6	1	0	1.797058	1.183109	-0.000008
7	1	0	2.330166	-0.266705	0.877182
8	1	0	2.330176	-0.266719	-0.877169
9	1	0	-2.409862	0.553844	-0.000010

A, B, C / GHz 49.4541231 4.3066557 4.0603883

FREQUENCIES AND ROTATIONAL CONSTANTS

INDEX NO.	DOF TYPE	CM-1(UNSCALED)	CM-1(SCALED BY)	SYMMETRY	IR-INTENSITY
1	vib	205.27	0.00	A	12.2501
2	vib	281.33	0.00	A	22.0062
3	vib	328.22	0.00	A	4.0957
4	vib	403.63	0.00	A	66.8689
5	vib	605.03	0.00	A	23.1118
6	vib	972.03	0.00	A	2.6672
7	vib	1012.39	0.00	A	17.2265
8	vib	1138.19	0.00	A	17.7691
9	vib	1140.20	0.00	A	0.0088
10	vib	1192.71	0.00	A	30.6411
11	vib	1294.83	0.00	A	309.9266
12	vib	1405.34	0.00	A	3.6765
13	vib	1449.86	0.00	A	13.2278
14	vib	1480.86	0.00	A	3.8487
15	vib	1507.09	0.00	A	11.3789
16	vib	1792.31	0.00	A	245.7958
17	vib	2957.15	0.00	A	63.2394
18	vib	2988.64	0.00	A	87.6228
19	vib	3052.78	0.00	A	39.1682
20	vib	3071.18	0.00	A	22.6698
21	vib	3832.60	0.00	A	74.4643
22	rot	1.6496120	-		
23	rot	0.1436546	-		
24	rot	0.1354400	-		

ZPE and THERMAL CONTRIBUTIONS

```
Eelectronic                  [kJ/mol]    = -549409.013
Ezpe                         [kJ/mol]    = +192.071
Eelectronic+Ezpe             [kJ/mol]    = -549216.942

Eelectronic                  [hartrees] = -209.258813
```

Ezpe [hartrees] = +0.073156
Eelectronic+Ezpe [hartrees] = -209.185657

Thermal Correction to Energy [kJ/mol] = +204.805
Thermal Correction to Enthalpy [kJ/mol] = +207.286
Thermal Correction to Gibbs [kJ/mol] = +122.107

#123 methylene-amino-methanol

Charge = 0 Multiplicity = 1 Stoichiometry C2H5NO

OPTIMIZED GEOMETRY

Center Number	Atomic Number	Atomic Type	Coordinates (Angstroms) X	Y	Z
1	6	0	1.719731	-0.029630	0.182019
2	6	0	-0.521516	0.502154	-0.123120
3	7	0	0.630445	-0.350137	-0.365699
4	8	0	-1.685513	-0.251800	0.107248
5	1	0	1.835243	0.844610	0.835675
6	1	0	2.605114	-0.636793	0.008870
7	1	0	-0.331101	1.207721	0.702356
8	1	0	-0.712864	1.073569	-1.032765
9	1	0	-1.514696	-0.858891	0.834373

A, B, C / GHz 31.9822562 4.6603633 4.4678112

FREQUENCIES AND ROTATIONAL CONSTANTS

INDEX NO.	DOF TYPE	CM-1(UNSCALED)	CM-1(SCALED BY)	SYMMETRY	IR-INTENSITY
1	vib	98.07	0.00	A	3.9673
2	vib	251.21	0.00	A	110.7528
3	vib	389.19	0.00	A	5.6531
4	vib	530.00	0.00	A	13.0152
5	vib	714.74	0.00	A	2.9576
6	vib	949.82	0.00	A	29.5417
7	vib	1055.19	0.00	A	76.4093
8	vib	1079.49	0.00	A	13.4927
9	vib	1095.68	0.00	A	111.0967
10	vib	1217.99	0.00	A	12.1628
11	vib	1253.19	0.00	A	18.6661
12	vib	1375.41	0.00	A	3.8079
13	vib	1426.72	0.00	A	68.5725
14	vib	1491.42	0.00	A	17.8917

15	vib	1504.09	0.00	A	3.7732
16	vib	1728.95	0.00	A	25.5795
17	vib	2914.99	0.00	A	81.3057
18	vib	2979.10	0.00	A	65.8586
19	vib	3064.49	0.00	A	19.5905
20	vib	3124.93	0.00	A	27.7784
21	vib	3808.78	0.00	A	28.0357
22	rot	1.0668132	-		
23	rot	0.1554530	-		
24	rot	0.1490301	-		

ZPE and THERMAL CONTRIBUTIONS

Eelectronic	[kJ/mol]	= -549359.767
Ezpe	[kJ/mol]	= +191.722
Eelectronic+Ezpe	[kJ/mol]	= -549168.045
Eelectronic	[hartrees]	= -209.240056
Ezpe	[hartrees]	= +0.073023
Eelectronic+Ezpe	[hartrees]	= -209.167033
Thermal Correction to Energy	[kJ/mol]	= +204.503
Thermal Correction to Enthalpy	[kJ/mol]	= +206.981
Thermal Correction to Gibbs	[kJ/mol]	= +120.419

#126 O-methyl-N-oxide-formaldehyde

Charge = 0 Multiplicity = 1 Stoichiometry C2H5NO

OPTIMIZED GEOMETRY

Center Number	Atomic Number	Atomic Type	Coordinates (Angstroms) X	Y	Z
1	6	0	-1.756378	0.056674	0.000000
2	6	0	1.674424	-0.132800	0.000000
3	7	0	-0.601340	-0.463859	-0.000000
4	8	0	0.400375	0.502565	-0.000000
5	1	0	-2.595832	-0.624601	0.000001
6	1	0	-1.918213	1.131462	0.000001
7	1	0	1.804303	-0.749090	0.891144
8	1	0	1.804300	-0.749097	-0.891139
9	1	0	2.403538	0.674571	-0.000004

```
A, B, C / GHz         41.6087644          4.9177569           4.5239963

--------------------------------------------------------------------------------
-------------------- FREQUENCIES AND ROTATIONAL CONSTANTS ----------------------
--------------------------------------------------------------------------------
INDEX NO. DOF TYPE  CM-1(UNSCALED)  CM-1(SCALED BY ) SYMMETRY IR-INTENSITY
   1        vib         117.85          0.00              A       10.0503
   2        vib         180.15          0.00              A        5.1622
   3        vib         342.09          0.00              A        9.5740
   4        vib         568.54          0.00              A        9.4396
   5        vib         795.41          0.00              A        1.0315
   6        vib         852.90          0.00              A       57.2137
   7        vib         978.61          0.00              A       33.6008
   8        vib        1083.86          0.00              A      192.1861
   9        vib        1175.03          0.00              A        1.2933
  10        vib        1175.76          0.00              A        3.4817
  11        vib        1235.24          0.00              A        6.1762
  12        vib        1427.82          0.00              A        4.5001
  13        vib        1466.12          0.00              A        1.6712
  14        vib        1479.07          0.00              A        5.8380
  15        vib        1508.39          0.00              A       15.9020
  16        vib        1691.18          0.00              A       12.0810
  17        vib        3020.80          0.00              A       62.3397
  18        vib        3083.25          0.00              A       39.5163
  19        vib        3093.53          0.00              A        7.9600
  20        vib        3126.11          0.00              A       18.7324
  21        vib        3219.64          0.00              A        5.2558
  22        rot      1.3879190          -
  23        rot      0.1640387          -
  24        rot      0.1509043          -
--------------------------------------------------------------------------------

--------------------------------------------------------------------------------
------------------------ ZPE and THERMAL CONTRIBUTIONS -------------------------
--------------------------------------------------------------------------------
Eelectronic                    [kJ/mol]   = -549256.852
Ezpe                           [kJ/mol]   = +189.138
Eelectronic+Ezpe               [kJ/mol]   = -549067.714

Eelectronic                    [hartrees] = -209.200858
Ezpe                           [hartrees] = +0.072039
Eelectronic+Ezpe               [hartrees] = -209.128819

Thermal Correction to Energy   [kJ/mol]   = +202.163
Thermal Correction to Enthalpy [kJ/mol]   = +204.642
Thermal Correction to Gibbs    [kJ/mol]   = +118.045
--------------------------------------------------------------------------------
```

#13 Z,Z 2-imino-ethanol

Charge = 0 Multiplicity = 1 Stoichiometry C2H5NO

OPTIMIZED GEOMETRY

Center Number	Atomic Number	Atomic Type	Coordinates (Angstroms) X	Y	Z
1	6	0	-0.777567	0.545945	0.000000
2	6	0	0.720336	0.631146	-0.000000
3	7	0	-1.330844	-0.591285	-0.000000
4	8	0	1.331947	-0.629478	0.000000
5	1	0	-1.319477	1.496550	0.000002
6	1	0	1.031555	1.213758	-0.878652
7	1	0	1.031556	1.213759	0.878650
8	1	0	0.606258	-1.274177	0.000000
9	1	0	-2.346171	-0.537618	0.000000

A, B, C / GHz 17.9164368 6.4762261 4.9003445

FREQUENCIES AND ROTATIONAL CONSTANTS

INDEX NO.	DOF TYPE	CM-1(UNSCALED)	CM-1(SCALED BY)	SYMMETRY	IR-INTENSITY
1	vib	220.09	0.00	A	0.0226
2	vib	307.20	0.00	A	13.3022
3	vib	442.49	0.00	A	87.6135
4	vib	645.19	0.00	A	33.3241
5	vib	725.34	0.00	A	23.9890
6	vib	882.38	0.00	A	23.7352
7	vib	1041.11	0.00	A	22.7666
8	vib	1104.93	0.00	A	105.5312
9	vib	1111.68	0.00	A	3.1149
10	vib	1177.61	0.00	A	36.1349
11	vib	1254.16	0.00	A	3.5314
12	vib	1339.05	0.00	A	21.6070
13	vib	1405.93	0.00	A	58.2726
14	vib	1460.02	0.00	A	65.1442
15	vib	1493.76	0.00	A	13.1785
16	vib	1720.43	0.00	A	61.3411
17	vib	2965.23	0.00	A	33.6236
18	vib	2976.08	0.00	A	33.1668
19	vib	3033.85	0.00	A	61.1030
20	vib	3487.41	0.00	A	1.5254
21	vib	3663.71	0.00	A	82.7140
22	rot	0.5976280	-		
23	rot	0.2160236	-		
24	rot	0.1634579	-		

ZPE and THERMAL CONTRIBUTIONS

Eelectronic	[kJ/mol]	= -549395.545
Ezpe	[kJ/mol]	= +194.140
Eelectronic+Ezpe	[kJ/mol]	= -549201.405
Eelectronic	[hartrees]	= -209.253683
Ezpe	[hartrees]	= +0.073944
Eelectronic+Ezpe	[hartrees]	= -209.179739
Thermal Correction to Energy	[kJ/mol]	= +205.907
Thermal Correction to Enthalpy	[kJ/mol]	= +208.389
Thermal Correction to Gibbs	[kJ/mol]	= +124.425

#130 1,2-oxazetidine

Charge = 0 Multiplicity = 1 Stoichiometry C2H5NO

OPTIMIZED GEOMETRY

Center Number	Atomic Number	Atomic Type	Coordinates (Angstroms) X	Y	Z
1	6	0	0.578646	0.857869	-0.068026
2	6	0	-0.891394	0.489663	0.094684
3	7	0	0.891275	-0.605437	-0.046820
4	8	0	-0.561117	-0.904499	-0.108456
5	1	0	1.041844	1.443752	0.724485
6	1	0	0.833505	1.288409	-1.035194
7	1	0	-1.301853	0.663806	1.095039
8	1	0	-1.590066	0.859175	-0.656543
9	1	0	1.143070	-0.866279	0.907652

A, B, C / GHz 12.7347343 12.1169942 6.9643787

FREQUENCIES AND ROTATIONAL CONSTANTS

INDEX NO.	DOF TYPE	CM-1(UNSCALED)	CM-1(SCALED BY)	SYMMETRY	IR-INTENSITY
1	vib	128.38	0.00	A	4.0720
2	vib	744.84	0.00	A	2.1893
3	vib	862.98	0.00	A	12.6503
4	vib	872.84	0.00	A	0.5614

5	vib	926.30	0.00	A	5.8405
6	vib	978.21	0.00	A	10.0613
7	vib	1010.94	0.00	A	42.1656
8	vib	1050.18	0.00	A	40.7436
9	vib	1153.86	0.00	A	3.3912
10	vib	1208.47	0.00	A	2.2385
11	vib	1254.26	0.00	A	18.3863
12	vib	1311.55	0.00	A	4.2351
13	vib	1350.24	0.00	A	0.7206
14	vib	1374.39	0.00	A	10.8328
15	vib	1510.09	0.00	A	0.3494
16	vib	1531.24	0.00	A	0.9499
17	vib	2996.48	0.00	A	75.0237
18	vib	3055.66	0.00	A	40.3332
19	vib	3069.58	0.00	A	30.9101
20	vib	3103.68	0.00	A	34.9936
21	vib	3391.42	0.00	A	3.9189
22	rot	0.4247850	-		
23	rot	0.4041794	-		
24	rot	0.2323067	-		

```
--------------------------------------------------------------------------------

--------------------------------------------------------------------------------
------------------------ ZPE and THERMAL CONTRIBUTIONS -------------------------
--------------------------------------------------------------------------------
Eelectronic                    [kJ/mol]    = -549176.263
Ezpe                           [kJ/mol]    = +196.700
Eelectronic+Ezpe               [kJ/mol]    = -548979.563

Eelectronic                    [hartrees] = -209.170163
Ezpe                           [hartrees] = +0.074919
Eelectronic+Ezpe               [hartrees] = -209.095244

Thermal Correction to Energy   [kJ/mol]    = +207.131
Thermal Correction to Enthalpy [kJ/mol]    = +209.612
Thermal Correction to Gibbs    [kJ/mol]    = +127.883
--------------------------------------------------------------------------------
```

#131 1,3-oxazetidine

Charge = 0 Multiplicity = 1 Stoichiometry C2H5NO

```
---------------------------------------------------------------------
---------------------- OPTIMIZED GEOMETRY ---------------------------
---------------------------------------------------------------------
Center     Atomic      Atomic              Coordinates (Angstroms)
Number     Number       Type             X           Y           Z
---------------------------------------------------------------------
```

1	6	0	1.007670	-0.026258	0.001683
2	6	0	-1.007670	-0.026258	0.001683
3	7	0	-0.000000	1.040591	-0.146195
4	8	0	0.000000	-1.050586	0.030025
5	1	0	1.599040	0.010273	0.924490
6	1	0	1.674448	-0.136850	-0.858009
7	1	0	-1.674448	-0.136850	-0.858009
8	1	0	-1.599040	0.010273	0.924490
9	1	0	-0.000000	1.688806	0.630002

A, B, C / GHz 12.9483491 12.7548106 7.1238313

FREQUENCIES AND ROTATIONAL CONSTANTS

INDEX NO.	DOF TYPE	CM-1(UNSCALED)	CM-1(SCALED BY)	SYMMETRY	IR-INTENSITY
1	vib	117.50	0.00	A	8.0880
2	vib	773.71	0.00	A	68.2324
3	vib	904.28	0.00	A	97.9553
4	vib	947.77	0.00	A	1.5293
5	vib	998.51	0.00	A	28.6335
6	vib	1012.68	0.00	A	15.7108
7	vib	1063.91	0.00	A	104.0293
8	vib	1070.95	0.00	A	0.5797
9	vib	1121.65	0.00	A	1.5711
10	vib	1144.89	0.00	A	0.4780
11	vib	1192.55	0.00	A	14.5543
12	vib	1332.88	0.00	A	2.5963
13	vib	1381.89	0.00	A	25.0628
14	vib	1420.25	0.00	A	6.8149
15	vib	1539.58	0.00	A	12.3565
16	vib	1569.01	0.00	A	0.3545
17	vib	2971.66	0.00	A	192.9142
18	vib	2981.50	0.00	A	43.5925
19	vib	3025.35	0.00	A	32.8349
20	vib	3028.18	0.00	A	94.1278
21	vib	3535.56	0.00	A	1.2973
22	rot	0.4319104	-		
23	rot	0.4254547	-		
24	rot	0.2376254	-		

ZPE and THERMAL CONTRIBUTIONS

```
Eelectronic                  [kJ/mol]   = -549294.967
Ezpe                         [kJ/mol]   = +198.186
Eelectronic+Ezpe             [kJ/mol]   = -549096.781

Eelectronic                  [hartrees] = -209.215375
```

```
Ezpe                             [hartrees] = +0.075485
Eelectronic+Ezpe                 [hartrees] = -209.139890

Thermal Correction to Energy   [kJ/mol]   = +208.533
Thermal Correction to Enthalpy [kJ/mol]   = +211.014
Thermal Correction to Gibbs    [kJ/mol]   = +129.361
--------------------------------------------------------------------------------
```

#132 3-methyl-oxaziridine

Charge = 0 Multiplicity = 1 Stoichiometry C2H5NO

OPTIMIZED GEOMETRY

Center Number	Atomic Number	Atomic Type	Coordinates (Angstroms) X	Y	Z
1	6	0	-0.106863	-0.071765	0.474697
2	6	0	-1.460848	0.047257	-0.159824
3	7	0	1.010271	0.719398	0.042594
4	8	0	0.888606	-0.747775	-0.240840
5	1	0	-0.105816	-0.269687	1.544804
6	1	0	-1.364632	0.208056	-1.234047
7	1	0	-2.036263	-0.865650	0.002052
8	1	0	-2.019212	0.876785	0.278299
9	1	0	0.751443	1.143960	-0.851777

A, B, C / GHz 18.0948799 7.0614388 6.1300441

FREQUENCIES AND ROTATIONAL CONSTANTS

INDEX NO.	DOF TYPE	CM-1(UNSCALED)	CM-1(SCALED BY)	SYMMETRY	IR-INTENSITY
1	vib	219.43	0.00	A	0.1631
2	vib	383.30	0.00	A	3.9688
3	vib	442.56	0.00	A	6.8867
4	vib	734.65	0.00	A	5.8351
5	vib	858.20	0.00	A	18.4103
6	vib	922.82	0.00	A	2.3123
7	vib	1005.32	0.00	A	17.9213
8	vib	1073.70	0.00	A	20.6687
9	vib	1203.13	0.00	A	6.6033
10	vib	1228.09	0.00	A	18.5126
11	vib	1287.30	0.00	A	28.3680
12	vib	1353.23	0.00	A	25.9339
13	vib	1407.36	0.00	A	6.4557
14	vib	1445.83	0.00	A	52.2490

15	vib	1479.33	0.00	A	6.3239
16	vib	1496.96	0.00	A	5.7368
17	vib	3028.50	0.00	A	9.9813
18	vib	3085.97	0.00	A	8.7245
19	vib	3090.39	0.00	A	14.3459
20	vib	3109.69	0.00	A	39.4819
21	vib	3384.67	0.00	A	2.0840
22	rot	0.6035802	-		
23	rot	0.2355442	-		
24	rot	0.2044763	-		

ZPE and THERMAL CONTRIBUTIONS

Eelectronic	[kJ/mol]	= -549222.503
Ezpe	[kJ/mol]	= +192.840
Eelectronic+Ezpe	[kJ/mol]	= -549029.663
Eelectronic	[hartrees]	= -209.187775
Ezpe	[hartrees]	= +0.073449
Eelectronic+Ezpe	[hartrees]	= -209.114326
Thermal Correction to Energy	[kJ/mol]	= +204.162
Thermal Correction to Enthalpy	[kJ/mol]	= +206.640
Thermal Correction to Gibbs	[kJ/mol]	= +123.816

#133 2-methyl-oxaziridine

Charge = 0 Multiplicity = 1 Stoichiometry C2H5NO

OPTIMIZED GEOMETRY

Center Number	Atomic Number	Atomic Type	Coordinates (Angstroms) X	Y	Z
1	6	0	0.942128	-0.599379	0.106111
2	6	0	-1.415995	-0.043907	0.144104
3	7	0	-0.158829	-0.034636	-0.597467
4	8	0	0.836102	0.797511	0.151560
5	1	0	0.754888	-1.119349	1.044482
6	1	0	1.746061	-1.004933	-0.501821
7	1	0	-1.273549	-0.083846	1.227697
8	1	0	-1.982395	0.849073	-0.114171
9	1	0	-1.978821	-0.918871	-0.187690

A, B, C / GHz 18.8637380 7.4299514 6.5052288

FREQUENCIES AND ROTATIONAL CONSTANTS

INDEX NO.	DOF TYPE	CM-1(UNSCALED)	CM-1(SCALED BY)	SYMMETRY	IR-INTENSITY
1	vib	226.70	0.00	A	0.9645
2	vib	408.19	0.00	A	9.4253
3	vib	445.88	0.00	A	2.6378
4	vib	722.80	0.00	A	12.7739
5	vib	828.95	0.00	A	21.7495
6	vib	981.66	0.00	A	7.4185
7	vib	1054.26	0.00	A	3.6920
8	vib	1126.95	0.00	A	6.5859
9	vib	1168.95	0.00	A	1.1593
10	vib	1199.55	0.00	A	4.9118
11	vib	1226.80	0.00	A	2.9831
12	vib	1315.99	0.00	A	21.2176
13	vib	1431.31	0.00	A	3.4194
14	vib	1471.96	0.00	A	6.4583
15	vib	1500.04	0.00	A	8.2091
16	vib	1539.58	0.00	A	3.0974
17	vib	3006.41	0.00	A	29.6496
18	vib	3052.95	0.00	A	38.5177
19	vib	3080.13	0.00	A	21.1493
20	vib	3110.33	0.00	A	17.1636
21	vib	3143.24	0.00	A	30.2119
22	rot	0.6292266	-		
23	rot	0.2478365	-		
24	rot	0.2169911	-		

ZPE and THERMAL CONTRIBUTIONS

```
Eelectronic                  [kJ/mol]   = -549191.801
Ezpe                         [kJ/mol]   = +191.659
Eelectronic+Ezpe             [kJ/mol]   = -549000.142

Eelectronic                  [hartrees] = -209.176081
Ezpe                         [hartrees] = +0.072999
Eelectronic+Ezpe             [hartrees] = -209.103082

Thermal Correction to Energy   [kJ/mol]   = +202.867
Thermal Correction to Enthalpy [kJ/mol]   = +205.348
Thermal Correction to Gibbs    [kJ/mol]   = +122.923
```

#138 nitrosoethane

Charge = 0 Multiplicity = 1 Stoichiometry C2H5NO

OPTIMIZED GEOMETRY

Center Number	Atomic Number	Atomic Type	Coordinates (Angstroms) X	Y	Z
1	6	0	0.417227	0.602291	-0.010447
2	6	0	1.631551	-0.317890	-0.088462
3	7	0	-0.753266	-0.180783	0.470113
4	8	0	-1.697742	-0.155490	-0.270476
5	1	0	0.172897	1.076036	-0.962696
6	1	0	0.574566	1.365754	0.757153
7	1	0	2.523458	0.259789	-0.328131
8	1	0	1.794420	-0.824780	0.862195
9	1	0	1.496784	-1.073803	-0.862051

A, B, C / GHz 26.8629114 4.7170807 4.5523654

FREQUENCIES AND ROTATIONAL CONSTANTS

INDEX NO.	DOF TYPE	CM-1(UNSCALED)	CM-1(SCALED BY)	SYMMETRY	IR-INTENSITY
1	vib	79.40	0.00	A	0.0184
2	vib	205.14	0.00	A	0.4162
3	vib	374.10	0.00	A	4.4297
4	vib	563.39	0.00	A	1.5243
5	vib	776.12	0.00	A	20.5473
6	vib	850.70	0.00	A	9.5389
7	vib	989.90	0.00	A	2.4258
8	vib	1040.25	0.00	A	5.7739
9	vib	1172.52	0.00	A	12.3543
10	vib	1254.81	0.00	A	5.5744
11	vib	1327.44	0.00	A	3.0053
12	vib	1409.23	0.00	A	2.1296
13	vib	1465.42	0.00	A	4.1679
14	vib	1493.82	0.00	A	8.6307
15	vib	1504.95	0.00	A	7.2162
16	vib	1656.05	0.00	A	93.3781
17	vib	3017.51	0.00	A	11.6852
18	vib	3041.12	0.00	A	17.5099
19	vib	3076.90	0.00	A	9.0755
20	vib	3108.84	0.00	A	18.9555
21	vib	3112.99	0.00	A	19.9702
22	rot	0.8960503	-		
23	rot	0.1573449	-		
24	rot	0.1518506	-		

```
------------------------------------------------------------------------------

------------------------------------------------------------------------------
----------------------- ZPE and THERMAL CONTRIBUTIONS ------------------------
------------------------------------------------------------------------------
Eelectronic                   [kJ/mol]    = -549236.07
Ezpe                          [kJ/mol]    = +188.535
Eelectronic+Ezpe              [kJ/mol]    = -549047.535

Eelectronic                   [hartrees] = -209.192942
Ezpe                          [hartrees] = +0.071809
Eelectronic+Ezpe              [hartrees] = -209.121133

Thermal Correction to Energy   [kJ/mol]   = +201.570
Thermal Correction to Enthalpy [kJ/mol]   = +204.049
Thermal Correction to Gibbs    [kJ/mol]   = +116.360
------------------------------------------------------------------------------
```

#15 cis 2-aziridinol

```
Charge = 0 Multiplicity = 1  Stoichiometry    C2H5NO

-------------------------------------------------------------------
---------------------- OPTIMIZED GEOMETRY --------------------------
-------------------------------------------------------------------
Center     Atomic      Atomic             Coordinates (Angstroms)
Number     Number       Type              X           Y           Z
-------------------------------------------------------------------
     1          6           0        0.211182   -0.021230    0.487094
     2          6           0       -0.964671   -0.662937   -0.144074
     3          7           0       -0.847593    0.810445   -0.022555
     4          8           0        1.414178    0.029147   -0.228674
     5          1           0        0.332316   -0.011293    1.566666
     6          1           0       -0.798648   -1.109413   -1.116385
     7          1           0       -1.749845   -1.111384    0.451316
     8          1           0       -0.553280    1.210261   -0.908157
     9          1           0        1.910114   -0.779466   -0.064284
-------------------------------------------------------------------
 A, B, C / GHz         18.2513624          7.1913381          6.2700414

------------------------------------------------------------------------------
-------------------- FREQUENCIES AND ROTATIONAL CONSTANTS --------------------
------------------------------------------------------------------------------
INDEX NO. DOF TYPE  CM-1(UNSCALED)  CM-1(SCALED BY ) SYMMETRY IR-INTENSITY
   1         vib          214.79         0.00              A      111.9950
   2         vib          426.92         0.00              A       13.7633
   3         vib          460.58         0.00              A       14.0688
   4         vib          760.78         0.00              A       49.1489
```

5	vib	862.97	0.00	A	39.4721
6	vib	946.92	0.00	A	18.9792
7	vib	964.73	0.00	A	20.6272
8	vib	1026.60	0.00	A	17.3604
9	vib	1110.63	0.00	A	21.2169
10	vib	1136.85	0.00	A	3.1975
11	vib	1210.15	0.00	A	20.3363
12	vib	1243.15	0.00	A	25.8477
13	vib	1263.52	0.00	A	91.7286
14	vib	1321.81	0.00	A	47.5409
15	vib	1414.33	0.00	A	27.9438
16	vib	1501.58	0.00	A	9.2617
17	vib	3097.04	0.00	A	39.6044
18	vib	3105.19	0.00	A	20.0166
19	vib	3191.16	0.00	A	16.7486
20	vib	3501.78	0.00	A	5.9581
21	vib	3803.92	0.00	A	32.2970
22	rot	0.6087999	-		
23	rot	0.2398772	-		
24	rot	0.2091461	-		

```
--------------------------------------------------------------------------------
------------------------ ZPE and THERMAL CONTRIBUTIONS -------------------------
--------------------------------------------------------------------------------
Eelectronic                   [kJ/mol]   = -549318.218
Ezpe                          [kJ/mol]   = +194.783
Eelectronic+Ezpe              [kJ/mol]   = -549123.435

Eelectronic                   [hartrees] = -209.224231
Ezpe                          [hartrees] = +0.074189
Eelectronic+Ezpe              [hartrees] = -209.150042

Thermal Correction to Energy   [kJ/mol]   = +206.057
Thermal Correction to Enthalpy [kJ/mol]   = +208.536
Thermal Correction to Gibbs    [kJ/mol]   = +125.890
--------------------------------------------------------------------------------
```

#156 Figure 15s

Charge = 0 Multiplicity = 1 Stoichiometry   C2H5NO

```
---------------------------------------------------------------------
---------------------- OPTIMIZED GEOMETRY ---------------------------
---------------------------------------------------------------------
Center     Atomic      Atomic             Coordinates (Angstroms)
Number     Number       Type             X           Y           Z
---------------------------------------------------------------------
```

1	6	0	-1.856243	0.028724	-0.044216
2	6	0	0.553913	0.473367	0.089176
3	7	0	-0.550914	-0.258072	0.042032
4	8	0	1.737638	-0.208216	-0.144827
5	1	0	-2.552725	-0.780171	0.065956
6	1	0	-2.168798	1.056965	-0.086246
7	1	0	0.506079	1.539581	-0.073089
8	1	0	-0.333575	-1.249201	0.071712
9	1	0	2.318292	-0.107489	0.616292

A, B, C / GHz 46.3967009 4.3773934 4.0542576

-------------------- FREQUENCIES AND ROTATIONAL CONSTANTS ----------------------
----

INDEX NO.	DOF TYPE	CM-1(UNSCALED)	CM-1(SCALED BY)	SYMMETRY	IR-INTENSITY
1	vib	164.16	0.00	A	2.1852
2	vib	317.53	0.00	A	27.0958
3	vib	360.44	0.00	A	174.1150
4	vib	394.10	0.00	A	139.8587
5	vib	502.40	0.00	A	77.9148
6	vib	588.88	0.00	A	4.7373
7	vib	608.94	0.00	A	58.3677
8	vib	937.69	0.00	A	74.9143
9	vib	1030.04	0.00	A	47.9665
10	vib	1145.66	0.00	A	228.3569
11	vib	1228.09	0.00	A	30.6505
12	vib	1307.09	0.00	A	22.1916
13	vib	1363.54	0.00	A	36.8541
14	vib	1407.20	0.00	A	166.1979
15	vib	1499.52	0.00	A	17.3043
16	vib	1645.80	0.00	A	82.2508
17	vib	3192.08	0.00	A	18.0472
18	vib	3198.12	0.00	A	17.3219
19	vib	3316.63	0.00	A	4.9792
20	vib	3522.81	0.00	A	29.8867
21	vib	3764.62	0.00	A	58.3570
22	rot	1.5476274	-		
23	rot	0.1460141	-		
24	rot	0.1352355	-		

------------------------ ZPE and THERMAL CONTRIBUTIONS -------------------------
----

Eelectronic [kJ/mol] = -549227.379
Ezpe [kJ/mol] = +188.385
Eelectronic+Ezpe [kJ/mol] = -549038.994

Eelectronic [hartrees] = -209.189632

```
Ezpe                           [hartrees] = +0.071752
Eelectronic+Ezpe               [hartrees] = -209.117880

Thermal Correction to Energy   [kJ/mol]   = +202.058
Thermal Correction to Enthalpy [kJ/mol]   = +204.537
Thermal Correction to Gibbs    [kJ/mol]   = +117.853
--------------------------------------------------------------------------------
```

#163 Figure 15t

Charge = 0 Multiplicity = 1 Stoichiometry C2H5NO

OPTIMIZED GEOMETRY

Center Number	Atomic Number	Atomic Type	Coordinates (Angstroms) X	Y	Z
1	6	0	-0.508441	0.697574	0.000084
2	6	0	-1.410291	-0.466178	-0.000062
3	7	0	1.477694	-0.573419	-0.000055
4	8	0	0.767473	0.571904	-0.000077
5	1	0	-0.832466	1.726938	0.000340
6	1	0	-1.235196	-1.101410	-0.878740
7	1	0	-1.235265	-1.101571	0.878516
8	1	0	-2.448691	-0.145484	-0.000079
9	1	0	0.780367	-1.328150	0.000826

A, B, C / GHz 18.7073540 6.4335950 4.9326442

FREQUENCIES AND ROTATIONAL CONSTANTS

INDEX NO.	DOF TYPE	CM-1(UNSCALED)	CM-1(SCALED BY)	SYMMETRY	IR-INTENSITY
1	vib	223.77	0.00	A	7.2230
2	vib	310.82	0.00	A	4.1834
3	vib	406.96	0.00	A	8.7446
4	vib	645.78	0.00	A	0.0898
5	vib	659.31	0.00	A	16.5090
6	vib	672.39	0.00	A	57.9360
7	vib	915.87	0.00	A	49.2066
8	vib	989.37	0.00	A	61.0471
9	vib	1022.99	0.00	A	0.2090
10	vib	1128.14	0.00	A	11.0356
11	vib	1356.45	0.00	A	43.5479
12	vib	1408.91	0.00	A	8.8114
13	vib	1460.03	0.00	A	10.1278
14	vib	1466.28	0.00	A	10.5587

15	vib	1488.94	0.00	A	26.0509
16	vib	1544.61	0.00	A	26.2505
17	vib	2969.20	0.00	A	9.4773
18	vib	2995.90	0.00	A	17.2619
19	vib	3130.06	0.00	A	5.5065
20	vib	3213.65	0.00	A	4.6210
21	vib	3337.45	0.00	A	31.5346
22	rot	0.6240102	-		
23	rot	0.2146016	-		
24	rot	0.1645353	-		

------------------------ ZPE and THERMAL CONTRIBUTIONS ------------------------

```
Eelectronic                    [kJ/mol]    = -549092.071
Ezpe                           [kJ/mol]    = +187.495
Eelectronic+Ezpe               [kJ/mol]    = -548904.576

Eelectronic                    [hartrees] = -209.138096
Ezpe                           [hartrees] = +0.071413
Eelectronic+Ezpe               [hartrees] = -209.066683

Thermal Correction to Energy   [kJ/mol]    = +199.677
Thermal Correction to Enthalpy [kJ/mol]    = +202.156
Thermal Correction to Gibbs    [kJ/mol]    = +117.685
```

#176 H–C̈–O–CH_2–NH_2

Charge = 0 Multiplicity = 1 Stoichiometry C2H5NO

---------------------- OPTIMIZED GEOMETRY --------------------------

Center Number	Atomic Number	Atomic Type	Coordinates (Angstroms) X	Y	Z
1	6	0	1.387918	-0.636398	0.145748
2	6	0	-0.633174	0.631840	0.146647
3	7	0	-1.402671	-0.519933	-0.030496
4	8	0	0.862133	0.484672	-0.192705
5	1	0	2.453582	-0.522490	-0.157260
6	1	0	-0.616152	0.953106	1.184535
7	1	0	-0.942260	1.440943	-0.507207
8	1	0	-1.008056	-1.299433	0.483663
9	1	0	-1.493940	-0.782621	-1.002991

```
A, B, C / GHz         18.8215263          6.1632786          5.0156982
```

```
--------------------------------------------------------------------------------
-------------------- FREQUENCIES AND ROTATIONAL CONSTANTS ----------------------
--------------------------------------------------------------------------------
INDEX NO. DOF TYPE  CM-1(UNSCALED)  CM-1(SCALED BY ) SYMMETRY IR-INTENSITY
   1        vib          63.73           0.00             A         8.2596
   2        vib         312.36           0.00             A        29.4337
   3        vib         418.04           0.00             A        28.9446
   4        vib         564.06           0.00             A        77.9112
   5        vib         639.27           0.00             A        31.0641
   6        vib         735.85           0.00             A        61.1085
   7        vib         832.19           0.00             A       330.3172
   8        vib         941.15           0.00             A         2.5783
   9        vib        1193.29           0.00             A        22.4249
  10        vib        1225.54           0.00             A         3.7146
  11        vib        1304.29           0.00             A        48.2502
  12        vib        1386.12           0.00             A        16.2512
  13        vib        1399.38           0.00             A        10.3835
  14        vib        1444.76           0.00             A        31.9966
  15        vib        1510.78           0.00             A         3.5056
  16        vib        1683.38           0.00             A        25.6653
  17        vib        2826.97           0.00             A       191.7020
  18        vib        3091.67           0.00             A        27.2734
  19        vib        3156.09           0.00             A        13.6555
  20        vib        3500.65           0.00             A        12.3732
  21        vib        3584.44           0.00             A        17.3380
  22        rot      0.6278185           -
  23        rot      0.2055848           -
  24        rot      0.1673057           -
--------------------------------------------------------------------------------
```

```
--------------------------------------------------------------------------------
------------------------ ZPE and THERMAL CONTRIBUTIONS -------------------------
--------------------------------------------------------------------------------
Eelectronic                    [kJ/mol]   = -549169.059
Ezpe                           [kJ/mol]   = +190.291
Eelectronic+Ezpe               [kJ/mol]   = -548978.768

Eelectronic                    [hartrees] = -209.167419
Ezpe                           [hartrees] = +0.072478
Eelectronic+Ezpe               [hartrees] = -209.094941

Thermal Correction to Energy   [kJ/mol]   = +203.269
Thermal Correction to Enthalpy [kJ/mol]   = +205.747
Thermal Correction to Gibbs    [kJ/mol]   = +118.169
--------------------------------------------------------------------------------
```

#178 H–$\ddot{C}$–O–NH–CH_3

Charge = 0 Multiplicity = 1 Stoichiometry C2H5NO

OPTIMIZED GEOMETRY

Center Number	Atomic Number	Atomic Type	Coordinates (Angstroms) X	Y	Z
1	6	0	1.334455	-0.691701	-0.045638
2	6	0	-1.341916	-0.516882	0.040039
3	7	0	-0.580363	0.699870	-0.183770
4	8	0	0.890220	0.494725	0.051320
5	1	0	2.423425	-0.537639	0.109194
6	1	0	-1.106670	-1.022738	0.981658
7	1	0	-2.386848	-0.201485	0.038846
8	1	0	-1.180135	-1.217293	-0.773843
9	1	0	-0.764221	1.373768	0.553567

A, B, C / GHz 18.7959962 6.5172382 5.0748008

FREQUENCIES AND ROTATIONAL CONSTANTS

INDEX NO.	DOF TYPE	CM-1(UNSCALED)	CM-1(SCALED BY)	SYMMETRY	IR-INTENSITY
1	vib	155.45	0.00	A	7.3941
2	vib	236.43	0.00	A	4.7047
3	vib	354.70	0.00	A	7.7697
4	vib	523.49	0.00	A	51.5762
5	vib	623.25	0.00	A	22.9240
6	vib	814.39	0.00	A	47.0799
7	vib	937.64	0.00	A	28.6607
8	vib	1006.45	0.00	A	5.7883
9	vib	1144.56	0.00	A	0.2005
10	vib	1197.83	0.00	A	18.0685
11	vib	1334.51	0.00	A	11.8023
12	vib	1361.15	0.00	A	11.8483
13	vib	1422.75	0.00	A	4.8308
14	vib	1452.46	0.00	A	7.9972
15	vib	1472.52	0.00	A	4.5512
16	vib	1516.22	0.00	A	11.2763
17	vib	2870.40	0.00	A	122.0037
18	vib	3005.49	0.00	A	25.3710
19	vib	3081.81	0.00	A	13.4670
20	vib	3136.09	0.00	A	6.0947
21	vib	3492.74	0.00	A	3.4460
22	rot	0.6269669	-		
23	rot	0.2173917	-		
24	rot	0.1692771	-		

ZPE and THERMAL CONTRIBUTIONS		
Eelectronic	[kJ/mol]	= -549027.014
Ezpe	[kJ/mol]	= +186.261
Eelectronic+Ezpe	[kJ/mol]	= -548840.753
Eelectronic	[hartrees]	= -209.113317
Ezpe	[hartrees]	= +0.070943
Eelectronic+Ezpe	[hartrees]	= -209.042374
Thermal Correction to Energy	[kJ/mol]	= +199.176
Thermal Correction to Enthalpy	[kJ/mol]	= +201.657
Thermal Correction to Gibbs	[kJ/mol]	= +115.454

#19 Figure 15d

Charge = 0 Multiplicity = 1 Stoichiometry C2H5NO

OPTIMIZED GEOMETRY

Center Number	Atomic Number	Atomic Type	Coordinates (Angstroms) X	Y	Z
1	6	0	-0.586277	0.953212	-0.003374
2	6	0	0.710824	0.646464	0.003263
3	7	0	-1.358006	-0.410567	-0.011651
4	8	0	1.228754	-0.670117	0.051434
5	1	0	1.466642	1.422198	0.011308
6	1	0	-2.081338	-0.360041	-0.720813
7	1	0	-1.820859	-0.528020	0.885400
8	1	0	-0.748692	-1.220475	-0.181915
9	1	0	2.112976	-0.676811	-0.323232

A, B, C / GHz 16.2515812 6.5794330 4.8161930

FREQUENCIES AND ROTATIONAL CONSTANTS

INDEX NO.	DOF TYPE	CM-1(UNSCALED)	CM-1(SCALED BY)	SYMMETRY	IR-INTENSITY
1	vib	158.54	0.00	A	5.6651
2	vib	220.74	0.00	A	119.0212
3	vib	305.97	0.00	A	48.1793
4	vib	454.07	0.00	A	23.9043

5	vib	623.21	0.00	A	13.1708
6	vib	689.80	0.00	A	6.7764
7	vib	802.02	0.00	A	19.1323
8	vib	960.46	0.00	A	4.8321
9	vib	974.14	0.00	A	10.1114
10	vib	1001.95	0.00	A	136.8915
11	vib	1211.66	0.00	A	75.0958
12	vib	1303.31	0.00	A	16.4750
13	vib	1348.83	0.00	A	72.5557
14	vib	1635.68	0.00	A	27.3293
15	vib	1646.77	0.00	A	30.3204
16	vib	1660.00	0.00	A	47.6515
17	vib	3161.60	0.00	A	20.9302
18	vib	3306.85	0.00	A	42.2000
19	vib	3497.74	0.00	A	48.0917
20	vib	3544.45	0.00	A	54.1369
21	vib	3832.31	0.00	A	40.9119
22	rot	0.5420944	-		
23	rot	0.2194663	-		
24	rot	0.1606509	-		

```
--------------------------------------------------------------------------------
----------------------- ZPE AND THERMAL CONTRIBUTIONS ------------------------
--------------------------------------------------------------------------------
Eelectronic                     [kJ/mol]   = -549144.694
Ezpe                            [kJ/mol]   = +193.436
Eelectronic+Ezpe                [kJ/mol]   = -548951.258

Eelectronic                     [hartrees] = -209.158139
Ezpe                            [hartrees] = +0.073676
Eelectronic+Ezpe                [hartrees] = -209.084463

Thermal Correction to Energy    [kJ/mol]   = +207.010
Thermal Correction to Enthalpy  [kJ/mol]   = +209.489
Thermal Correction to Gibbs     [kJ/mol]   = +122.002
--------------------------------------------------------------------------------
```

#2 acetamide

Charge = 0 Multiplicity = 1 Stoichiometry   C2H5NO

```
-------------------------------------------------------------------
---------------------- OPTIMIZED GEOMETRY --------------------------
-------------------------------------------------------------------
Center     Atomic      Atomic              Coordinates (Angstroms)
Number     Number       Type             X           Y           Z
-------------------------------------------------------------------
```

8	-0.363513000	1.328263000	0.001128000
6	-0.076498000	0.147236000	-0.007334000
6	1.361187000	-0.337360000	-0.000701000
1	1.469685000	-1.382458000	-0.288918000
1	1.940982000	0.287773000	-0.675802000
1	1.768768000	-0.207675000	1.002709000
7	-1.027074000	-0.831302000	-0.006501000
1	-1.993598000	-0.557102000	0.034355000
1	-0.796341000	-1.806778000	0.012353000

Rotational constants (GHZ): 10.8737276 9.2791366 5.1663717

25.6342	4.6659
155.8186	181.4092
428.1026	4.372
520.3752	5.5078
548.1844	13.476
667.7389	8.9563
839.2249	2.5951
984.242	10.7319
1056.7042	4.064
1121.4162	0.3973
1341.674	124.2662
1399.8629	55.7329
1473.2835	8.2919
1491.591	8.142
1619.4114	95.7197
1773.9548	339.6804
3043.6874	7.8273
3105.9261	14.6595
3131.809	8.9581
3591.7292	30.4219
3728.5004	33.7548

- Thermochemistry -

Temperature 298.150 Kelvin. Pressure 1.00000 Atm.

Zero-point correction=	0.073013 (Hartree/Particle)
Thermal correction to Energy=	0.078367
Thermal correction to Enthalpy=	0.079311
Thermal correction to Gibbs Free Energy=	0.044303
Sum of electronic and zero-point Energies=	-209.229887
Sum of electronic and thermal Energies=	-209.224533
Sum of electronic and thermal Enthalpies=	-209.223589
Sum of electronic and thermal Free Energies=	-209.258597

#21 Figure 15e

Charge = 0 Multiplicity = 1 Stoichiometry C2H5NO

OPTIMIZED GEOMETRY

Center Number	Atomic Number	Atomic Type	Coordinates (Angstroms) X	Y	Z
1	6	0	-0.930091	-0.753240	0.031379
2	6	0	-0.719104	0.738921	-0.145670
3	7	0	0.763984	0.524753	0.169157
4	8	0	0.628006	-0.846913	-0.216839
5	1	0	-0.990186	-0.943123	1.118380
6	1	0	-1.218029	1.462725	0.499449
7	1	0	-0.730746	1.079856	-1.185210
8	1	0	1.494519	1.011688	-0.373417
9	1	0	0.967685	0.576806	1.177159

A, B, C / GHz 12.6588216 11.7166943 7.0730836

FREQUENCIES AND ROTATIONAL CONSTANTS

INDEX NO.	DOF TYPE	CM-1(UNSCALED)	CM-1(SCALED BY)	SYMMETRY	IR-INTENSITY
1	vib	306.29	0.00	A	6.5470
2	vib	422.82	0.00	A	22.1436
3	vib	642.14	0.00	A	2.5380
4	vib	743.43	0.00	A	20.2463
5	vib	854.09	0.00	A	3.6770
6	vib	913.68	0.00	A	3.2914
7	vib	922.45	0.00	A	75.0711
8	vib	968.36	0.00	A	26.7227
9	vib	1157.74	0.00	A	35.6997
10	vib	1178.68	0.00	A	3.8621
11	vib	1221.45	0.00	A	9.7483
12	vib	1280.48	0.00	A	1.1273
13	vib	1307.82	0.00	A	21.7885
14	vib	1316.13	0.00	A	20.5849
15	vib	1490.98	0.00	A	3.7745
16	vib	1576.10	0.00	A	20.2543
17	vib	2888.95	0.00	A	143.7573
18	vib	3006.30	0.00	A	73.1048
19	vib	3082.21	0.00	A	35.7808
20	vib	3197.13	0.00	A	222.5227
21	vib	3311.15	0.00	A	3.9138
22	rot	0.4222528	-		
23	rot	0.3908269	-		
24	rot	0.2359327	-		

```
----------------------- ZPE and THERMAL CONTRIBUTIONS ------------------------
------------------------------------------------------------------------------
Eelectronic                    [kJ/mol]    = -548872.561
Ezpe                           [kJ/mol]    = +190.136
Eelectronic+Ezpe               [kJ/mol]    = -548682.425

Eelectronic                    [hartrees] = -209.054489
Ezpe                           [hartrees] = +0.072419
Eelectronic+Ezpe               [hartrees] = -208.982070

Thermal Correction to Energy   [kJ/mol]    = +200.822
Thermal Correction to Enthalpy [kJ/mol]    = +203.300
Thermal Correction to Gibbs    [kJ/mol]    = +122.136
------------------------------------------------------------------------------
```

#23 amino-acetaldehyde

Charge = 0 Multiplicity = 1 Stoichiometry   C2H5NO

```
--------------------------------------------------------------------
---------------------- OPTIMIZED GEOMETRY --------------------------
--------------------------------------------------------------------
```

Center Number	Atomic Number	Atomic Type	Coordinates (Angstroms) X	Y	Z
1	6	0	-0.758674	-0.313659	0.195571
2	6	0	0.495113	0.531152	0.085855
3	7	0	1.676233	-0.312513	-0.057763
4	8	0	-1.846030	0.034373	-0.183081
5	1	0	0.572015	1.071164	1.035845
6	1	0	0.338117	1.281816	-0.696649
7	1	0	2.519101	0.192351	0.178712
8	1	0	1.779434	-0.636665	-1.010904
9	1	0	-0.592687	-1.301024	0.673426

A, B, C / GHz   33.4719497   4.2840339   4.0540002

```
------------------------------------------------------------------------------
------------------- FREQUENCIES AND ROTATIONAL CONSTANTS ----------------------
------------------------------------------------------------------------------
```

INDEX NO.	DOF TYPE	CM-1(UNSCALED)	CM-1(SCALED BY)	SYMMETRY	IR-INTENSITY
1	vib	88.37	0.00	A	9.0922
2	vib	245.80	0.00	A	41.5304
3	vib	346.61	0.00	A	11.7967
4	vib	531.97	0.00	A	4.7939
5	vib	729.74	0.00	A	9.5399
6	vib	816.50	0.00	A	147.7441
7	vib	991.65	0.00	A	8.3809

8	vib	1061.72	0.00	A	24.1080
9	vib	1095.80	0.00	A	3.3616
10	vib	1190.55	0.00	A	0.4725
11	vib	1305.25	0.00	A	13.1691
12	vib	1371.53	0.00	A	0.1742
13	vib	1405.70	0.00	A	8.0414
14	vib	1468.71	0.00	A	8.5116
15	vib	1668.83	0.00	A	21.5177
16	vib	1813.03	0.00	A	168.1661
17	vib	2886.46	0.00	A	95.4388
18	vib	2993.07	0.00	A	37.8506
19	vib	3033.56	0.00	A	16.8028
20	vib	3511.52	0.00	A	0.4562
21	vib	3590.70	0.00	A	4.1764
22	rot	1.1165041	-		
23	rot	0.1429000	-		
24	rot	0.1352269	-		

```
--------------------------------------------------------------------------------

--------------------------------------------------------------------------------
------------------------ ZPE and THERMAL CONTRIBUTIONS -------------------------
--------------------------------------------------------------------------------
Eelectronic                    [kJ/mol]   = -549408.58
Ezpe                           [kJ/mol]   = +192.281
Eelectronic+Ezpe               [kJ/mol]   = -549216.299

Eelectronic                    [hartrees] = -209.258648
Ezpe                           [hartrees] = +0.073236
Eelectronic+Ezpe               [hartrees] = -209.185412

Thermal Correction to Energy   [kJ/mol]   = +205.330
Thermal Correction to Enthalpy [kJ/mol]   = +207.808
Thermal Correction to Gibbs    [kJ/mol]   = +120.453
--------------------------------------------------------------------------------
```

#24 aziridine-N-oxide

Charge = 0 Multiplicity = 1 Stoichiometry   C2H5NO

```
---------------------------------------------------------------------
---------------------- OPTIMIZED GEOMETRY --------------------------
---------------------------------------------------------------------
```

Center Number	Atomic Number	Atomic Type	Coordinates (Angstroms) X	Y	Z
1	6	0	0.857266	0.745506	-0.141361
2	6	0	0.857267	-0.745505	-0.141361
3	7	0	-0.317823	-0.000001	0.409651

```
      4          8          0      -1.483410   -0.000000   -0.204566
      5          1          0       1.479120    1.275633    0.567033
      6          1          0       0.594280    1.261034   -1.051107
      7          1          0       0.594281   -1.261034   -1.051107
      8          1          0       1.479121   -1.275632    0.567032
      9          1          0      -0.341955   -0.000001    1.433448
 ---------------------------------------------------------------------
A, B, C / GHz        17.8799969          7.4661646          6.3913754

 --------------------------------------------------------------------------------
 -------------------- FREQUENCIES AND ROTATIONAL CONSTANTS ----------------------
 --------------------------------------------------------------------------------
 INDEX NO. DOF TYPE  CM-1(UNSCALED)  CM-1(SCALED BY ) SYMMETRY IR-INTENSITY
    1        vib        409.31           0.00              A        5.5345
    2        vib        426.21           0.00              A       14.8352
    3        vib        678.94           0.00              A       23.3610
    4        vib        754.95           0.00              A        2.6058
    5        vib        818.50           0.00              A        1.3924
    6        vib        919.28           0.00              A        1.0002
    7        vib       1020.33           0.00              A       55.0317
    8        vib       1099.90           0.00              A        4.6449
    9        vib       1101.77           0.00              A       65.3671
   10        vib       1128.46           0.00              A        3.3484
   11        vib       1154.38           0.00              A       45.1894
   12        vib       1207.59           0.00              A        2.2562
   13        vib       1227.21           0.00              A       10.6212
   14        vib       1431.24           0.00              A        7.2294
   15        vib       1454.95           0.00              A        5.0311
   16        vib       1482.55           0.00              A        2.8219
   17        vib       3131.97           0.00              A        8.3453
   18        vib       3136.49           0.00              A        6.4125
   19        vib       3242.62           0.00              A        1.3381
   20        vib       3254.28           0.00              A        0.0312
   21        vib       3309.80           0.00              A       22.5508
   22        rot      0.5964125          -
   23        rot      0.2490444          -
   24        rot      0.2131933          -
 --------------------------------------------------------------------------------

 --------------------------------------------------------------------------------
 ------------------------ ZPE and THERMAL CONTRIBUTIONS -------------------------
 --------------------------------------------------------------------------------
 Eelectronic                    [kJ/mol]   = -549101.877
 Ezpe                           [kJ/mol]   = +193.738
 Eelectronic+Ezpe               [kJ/mol]   = -548908.139

 Eelectronic                    [hartrees] = -209.141831
 Ezpe                           [hartrees] = +0.073791
 Eelectronic+Ezpe               [hartrees] = -209.068040
```

```
Thermal Correction to Energy   [kJ/mol]   = +204.059
Thermal Correction to Enthalpy [kJ/mol]   = +206.538
Thermal Correction to Gibbs    [kJ/mol]   = +125.788
--------------------------------------------------------------------------------
```

#28 Figure 15f

```
Charge = 0 Multiplicity = 1  Stoichiometry    C2H5NO

---------------------------------------------------------------------
---------------------- OPTIMIZED GEOMETRY ---------------------------
---------------------------------------------------------------------
Center     Atomic      Atomic             Coordinates (Angstroms)
Number     Number       Type             X           Y           Z
---------------------------------------------------------------------
     1          6           0       -0.490737    0.693024   -0.000000
     2          6           0       -1.469375   -0.421058    0.000000
     3          7           0        0.785993    0.442198    0.000000
     4          8           0        1.343774   -0.694280   -0.000000
     5          1           0       -0.766469    1.736617   -0.000000
     6          1           0       -1.322720   -1.062180   -0.873196
     7          1           0       -2.489016   -0.043099    0.000006
     8          1           0       -1.322712   -1.062186    0.873190
     9          1           0        1.409446    1.257904    0.000000
---------------------------------------------------------------------
 A, B, C / GHz         19.0172843          6.3095501          4.8782482

--------------------------------------------------------------------------------
-------------------- FREQUENCIES AND ROTATIONAL CONSTANTS ----------------------
--------------------------------------------------------------------------------
INDEX NO. DOF TYPE  CM-1(UNSCALED)  CM-1(SCALED BY ) SYMMETRY IR-INTENSITY
   1        vib          82.07          0.00              A        1.4272
   2        vib         265.05          0.00              A       12.8322
   3        vib         455.92          0.00              A       32.4121
   4        vib         714.49          0.00              A       30.2512
   5        vib         739.04          0.00              A       10.9527
   6        vib         928.06          0.00              A       21.4763
   7        vib         963.50          0.00              A        3.7587
   8        vib        1045.22          0.00              A        0.6838
   9        vib        1121.41          0.00              A        5.2622
  10        vib        1156.95          0.00              A       65.6837
  11        vib        1389.19          0.00              A       87.1052
  12        vib        1425.84          0.00              A       44.5414
  13        vib        1465.61          0.00              A       11.0437
  14        vib        1479.65          0.00              A        1.8831
  15        vib        1540.88          0.00              A        4.5401
  16        vib        1656.76          0.00              A       90.8264
  17        vib        3020.23          0.00              A       11.2330
```

18	vib	3057.13	0.00	A	10.5878
19	vib	3124.45	0.00	A	13.0179
20	vib	3206.95	0.00	A	5.3028
21	vib	3317.36	0.00	A	13.1439
22	rot	0.6343483	-		
23	rot	0.2104639	-		
24	rot	0.1627208	-		

ZPE and THERMAL CONTRIBUTIONS

Eelectronic	[kJ/mol]	= -549271.461
Ezpe	[kJ/mol]	= +192.334
Eelectronic+Ezpe	[kJ/mol]	= -549079.127
Eelectronic	[hartrees]	= -209.206422
Ezpe	[hartrees]	= +0.073256
Eelectronic+Ezpe	[hartrees]	= -209.133166
Thermal Correction to Energy	[kJ/mol]	= +204.750
Thermal Correction to Enthalpy	[kJ/mol]	= +207.228
Thermal Correction to Gibbs	[kJ/mol]	= +120.823

#3 Figure 15u

Charge = 0 Multiplicity = 1 Stoichiometry C2H5NO

OPTIMIZED GEOMETRY

Center Number	Atomic Number	Atomic Type	Coordinates (Angstroms) X	Y	Z
1	6	0	1.148005	0.729945	0.027460
2	6	0	-1.415479	0.062127	-0.179207
3	7	0	-0.103201	0.011607	0.459789
4	8	0	0.852039	-0.756290	-0.228487
5	1	0	0.816504	1.200049	-0.918177
6	1	0	-1.259094	0.263869	-1.234200
7	1	0	-1.933267	-0.888389	-0.048817
8	1	0	-1.993594	0.867229	0.274460
9	1	0	-0.119610	-0.226120	1.446589

A, B, C / GHz 18.4161009 7.2307510 6.2978684

```
-------------------- FREQUENCIES AND ROTATIONAL CONSTANTS ---------------------
--------------------------------------------------------------------------------
INDEX NO. DOF TYPE  CM-1(UNSCALED)  CM-1(SCALED BY ) SYMMETRY IR-INTENSITY
   1        vib         214.06          0.00             A         0.4782
   2        vib         352.04          0.00             A         2.5650
   3        vib         438.61          0.00             A        11.5038
   4        vib         542.32          0.00             A        24.8605
   5        vib         818.38          0.00             A        17.0138
   6        vib         914.95          0.00             A         8.2722
   7        vib        1022.32          0.00             A        25.9313
   8        vib        1108.19          0.00             A        15.2134
   9        vib        1158.63          0.00             A        12.2823
  10        vib        1189.54          0.00             A        24.9718
  11        vib        1220.51          0.00             A         2.0051
  12        vib        1343.96          0.00             A         8.8798
  13        vib        1443.78          0.00             A         4.5665
  14        vib        1448.81          0.00             A         6.2342
  15        vib        1480.86          0.00             A         7.8903
  16        vib        1493.77          0.00             A        11.4611
  17        vib        2878.46          0.00             A       123.6950
  18        vib        3039.95          0.00             A        24.7136
  19        vib        3108.38          0.00             A        11.8710
  20        vib        3157.71          0.00             A         2.7970
  21        vib        3493.27          0.00             A        25.3059
  22        rot      0.6142950          -
  23        rot      0.2411919          -
  24        rot      0.2100743          -
--------------------------------------------------------------------------------

--------------------------------------------------------------------------------
------------------------ ZPE and THERMAL CONTRIBUTIONS ------------------------
--------------------------------------------------------------------------------
Eelectronic                     [kJ/mol]   = -548964.246
Ezpe                            [kJ/mol]   = +190.617
Eelectronic+Ezpe                [kJ/mol]   = -548773.629

Eelectronic                     [hartrees] = -209.089410
Ezpe                            [hartrees] = +0.072602
Eelectronic+Ezpe                [hartrees] = -209.016808

Thermal Correction to Energy    [kJ/mol]   = +202.342
Thermal Correction to Enthalpy  [kJ/mol]   = +204.821
Thermal Correction to Gibbs     [kJ/mol]   = +121.432
--------------------------------------------------------------------------------
```

#30 N-methylene-N-oxide-methanamine

Charge = 0 Multiplicity = 1 Stoichiometry   C2H5NO

OPTIMIZED GEOMETRY

Center Number	Atomic Number	Atomic Type	Coordinates (Angstroms) X	Y	Z
1	6	0	1.084109	-0.819874	0.000000
2	6	0	-1.286283	-0.384318	-0.000000
3	7	0	0.126432	0.061510	0.000000
4	8	0	0.282444	1.313987	0.000000
5	1	0	0.837833	-1.866455	-0.000000
6	1	0	2.097637	-0.456046	-0.000000
7	1	0	-1.754896	0.036678	0.885430
8	1	0	-1.754896	0.036678	-0.885431
9	1	0	-1.357207	-1.468176	0.000000

A, B, C / GHz 11.3052812 10.0607088 5.5066955

FREQUENCIES AND ROTATIONAL CONSTANTS

INDEX NO.	DOF TYPE	CM-1(UNSCALED)	CM-1(SCALED BY)	SYMMETRY	IR-INTENSITY
1	vib	166.72	0.00	A	1.3754
2	vib	472.80	0.00	A	4.1795
3	vib	530.02	0.00	A	0.6341
4	vib	558.88	0.00	A	16.6874
5	vib	689.90	0.00	A	14.5976
6	vib	805.04	0.00	A	51.9664
7	vib	861.91	0.00	A	0.1330
8	vib	1083.25	0.00	A	6.3376
9	vib	1124.42	0.00	A	58.9842
10	vib	1146.96	0.00	A	0.0038
11	vib	1354.48	0.00	A	44.8107
12	vib	1426.38	0.00	A	1.5281
13	vib	1458.16	0.00	A	48.3732
14	vib	1469.99	0.00	A	10.2608
15	vib	1497.52	0.00	A	5.3039
16	vib	1639.55	0.00	A	144.5555
17	vib	3065.16	0.00	A	11.0412
18	vib	3149.03	0.00	A	3.5341
19	vib	3157.27	0.00	A	9.1216
20	vib	3184.54	0.00	A	1.0894
21	vib	3309.35	0.00	A	0.3986
22	rot	0.3771036	-		
23	rot	0.3355891	-		
24	rot	0.1836836	-		

```
------------------------ ZPE and THERMAL CONTRIBUTIONS ------------------------
-------------------------------------------------------------------------------
Eelectronic                    [kJ/mol]   = -549248.075
Ezpe                           [kJ/mol]   = +192.307
Eelectronic+Ezpe               [kJ/mol]   = -549055.768

Eelectronic                    [hartrees] = -209.197515
Ezpe                           [hartrees] = +0.073246
Eelectronic+Ezpe               [hartrees] = -209.124269

Thermal Correction to Energy   [kJ/mol]   = +203.965
Thermal Correction to Enthalpy [kJ/mol]   = +206.443
Thermal Correction to Gibbs    [kJ/mol]   = +122.666
-------------------------------------------------------------------------------
```

#31 Figure 15g

Charge = 0 Multiplicity = 1 Stoichiometry   C2H5NO

```
--------------------------------------------------------------------
---------------------- OPTIMIZED GEOMETRY --------------------------
--------------------------------------------------------------------
Center     Atomic      Atomic             Coordinates (Angstroms)
Number     Number       Type             X           Y           Z
--------------------------------------------------------------------
     1          6           0       -0.742638   -0.263711    0.000000
     2          6           0        0.380421    0.553262    0.000000
     3          7           0        1.673886   -0.153405   -0.000000
     4          8           0       -1.935350    0.024290   -0.000000
     5          1           0        0.432556    1.626873   -0.000000
     6          1           0        2.258615    0.017374   -0.823364
     7          1           0        1.432337   -1.153362    0.000000
     8          1           0        2.258615    0.017374    0.823363
     9          1           0       -0.443230   -1.366049    0.000000
--------------------------------------------------------------------
A, B, C / GHz         41.8042728          4.1760206          3.8763336

-------------------------------------------------------------------------------
-------------------- FREQUENCIES AND ROTATIONAL CONSTANTS ---------------------
-------------------------------------------------------------------------------
INDEX NO. DOF TYPE  CM-1(UNSCALED)  CM-1(SCALED BY ) SYMMETRY IR-INTENSITY
   1        vib         123.28          0.00               A       46.9134
   2        vib         329.16          0.00               A        5.3501
   3        vib         339.45          0.00               A       26.6554
   4        vib         484.58          0.00               A       68.1236
   5        vib         584.31          0.00               A       27.5182
   6        vib         939.15          0.00               A       43.2559
   7        vib         974.81          0.00               A        0.0253
```

8	vib	1029.52	0.00	A	31.8717
9	vib	1070.31	0.00	A	10.7232
10	vib	1202.58	0.00	A	51.6364
11	vib	1359.14	0.00	A	28.9410
12	vib	1446.70	0.00	A	41.0979
13	vib	1463.41	0.00	A	22.5385
14	vib	1619.75	0.00	A	20.8719
15	vib	1676.80	0.00	A	61.1915
16	vib	1733.88	0.00	A	509.8741
17	vib	2523.68	0.00	A	338.6961
18	vib	3253.47	0.00	A	1.1473
19	vib	3300.17	0.00	A	73.7720
20	vib	3319.53	0.00	A	14.0941
21	vib	3354.36	0.00	A	22.0630
22	rot	1.3944404	-		
23	rot	0.1392971	-		
24	rot	0.1293006	-		

```
--------------------------------------------------------------------------------

--------------------------------------------------------------------------------
------------------------ ZPE and THERMAL CONTRIBUTIONS -------------------------
--------------------------------------------------------------------------------
Eelectronic                    [kJ/mol]   = -549248.393
Ezpe                           [kJ/mol]   = +192.168
Eelectronic+Ezpe               [kJ/mol]   = -549056.225

Eelectronic                    [hartrees] = -209.197636
Ezpe                           [hartrees] = +0.073193
Eelectronic+Ezpe               [hartrees] = -209.124443

Thermal Correction to Energy   [kJ/mol]   = +204.978
Thermal Correction to Enthalpy [kJ/mol]   = +207.457
Thermal Correction to Gibbs    [kJ/mol]   = +121.366
--------------------------------------------------------------------------------
```

#32 Figure 15h

Charge = 0 Multiplicity = 1 Stoichiometry   C2H5NO

```
---------------------------------------------------------------------
--------------------- OPTIMIZED GEOMETRY --------------------------
---------------------------------------------------------------------
```

Center Number	Atomic Number	Atomic Type	Coordinates (Angstroms) X	Y	Z
1	6	0	0.795012	0.261114	0.432436
2	6	0	1.680713	-0.367903	-0.327977
3	7	0	-0.491723	0.473990	-0.284474

4	8	0	-1.570171	-0.401902	0.113045
5	1	0	2.632317	-0.548717	0.179496
6	1	0	-0.224883	0.260146	-1.258242
7	1	0	0.737564	0.654160	1.445656
8	1	0	-0.906742	1.402017	-0.189731
9	1	0	-1.089175	-1.229595	0.283028

A, B, C / GHz 23.8933670 4.9249970 4.7498720

--------------------- FREQUENCIES AND ROTATIONAL CONSTANTS ---------------------

INDEX NO.	DOF TYPE	CM-1(UNSCALED)	CM-1(SCALED BY)	SYMMETRY	IR-INTENSITY
1	vib	124.39	0.00	A	20.2659
2	vib	361.30	0.00	A	58.4192
3	vib	411.92	0.00	A	50.7386
4	vib	459.69	0.00	A	60.5557
5	vib	690.40	0.00	A	55.3764
6	vib	747.21	0.00	A	13.9752
7	vib	879.50	0.00	A	144.2405
8	vib	921.23	0.00	A	26.9276
9	vib	976.93	0.00	A	10.2216
10	vib	1099.34	0.00	A	42.2318
11	vib	1247.64	0.00	A	19.5633
12	vib	1276.67	0.00	A	29.3268
13	vib	1320.41	0.00	A	40.1902
14	vib	1475.20	0.00	A	47.0581
15	vib	1542.75	0.00	A	20.0480
16	vib	1576.79	0.00	A	48.1657
17	vib	3053.46	0.00	A	45.0803
18	vib	3094.43	0.00	A	55.6135
19	vib	3252.80	0.00	A	35.0562
20	vib	3439.95	0.00	A	10.7490
21	vib	3677.96	0.00	A	42.5862
22	rot	0.7969969	-		
23	rot	0.1642802	-		
24	rot	0.1584387	-		

------------------------ ZPE and THERMAL CONTRIBUTIONS ------------------------

```
Eelectronic                   [kJ/mol]   = -548974.708
Ezpe                          [kJ/mol]   = +189.188
Eelectronic+Ezpe              [kJ/mol]   = -548785.520

Eelectronic                   [hartrees] = -209.093395
Ezpe                          [hartrees] = +0.072058
Eelectronic+Ezpe              [hartrees] = -209.021337
```

```
Thermal Correction to Energy   [kJ/mol]   = +201.946
Thermal Correction to Enthalpy [kJ/mol]   = +204.424
Thermal Correction to Gibbs    [kJ/mol]   = +118.365
--------------------------------------------------------------------------------
```

#38 $CH_3-\ddot{C}-NH-OH$

```
Charge = 0 Multiplicity = 1  Stoichiometry    C2H5NO

----------------------------------------------------------------------
---------------------- OPTIMIZED GEOMETRY --------------------------
----------------------------------------------------------------------
Center     Atomic      Atomic              Coordinates (Angstroms)
Number     Number       Type             X           Y           Z
----------------------------------------------------------------------
     1          6           0       -0.655138    0.779004    0.016341
     2          6           0       -1.516829   -0.434368   -0.017239
     3          7           0        0.596054    0.658967    0.015318
     4          8           0        1.437800   -0.616945   -0.109763
     5          1           0       -2.355396   -0.263154   -0.691925
     6          1           0       -1.959619   -0.550553    0.976352
     7          1           0       -1.000266   -1.357232   -0.289760
     8          1           0        1.265452    1.411856   -0.000379
     9          1           0        1.406856   -0.985951    0.781974
----------------------------------------------------------------------
A, B, C / GHz         17.4296096          5.7895608          4.5251913

--------------------------------------------------------------------------------
-------------------- FREQUENCIES AND ROTATIONAL CONSTANTS ----------------------
--------------------------------------------------------------------------------
INDEX NO. DOF TYPE  CM-1(UNSCALED)  CM-1(SCALED BY ) SYMMETRY IR-INTENSITY
   1        vib          71.18          0.00              A        7.2801
   2        vib         170.78          0.00              A       98.2329
   3        vib         248.22          0.00              A       38.0843
   4        vib         433.14          0.00              A       12.7307
   5        vib         496.71          0.00              A      186.7947
   6        vib         684.24          0.00              A       74.0894
   7        vib         693.25          0.00              A       92.9353
   8        vib         934.94          0.00              A       37.3441
   9        vib        1026.25          0.00              A        5.4962
  10        vib        1087.38          0.00              A        6.4332
  11        vib        1209.77          0.00              A       28.6818
  12        vib        1341.32          0.00              A       10.5432
  13        vib        1381.84          0.00              A        9.9192
  14        vib        1457.91          0.00              A        8.1179
  15        vib        1487.12          0.00              A        9.0238
  16        vib        1602.58          0.00              A       55.5368
  17        vib        3012.76          0.00              A       14.9435
```

18	vib	3070.69	0.00	A	13.6897
19	vib	3095.99	0.00	A	6.8775
20	vib	3603.14	0.00	A	51.1137
21	vib	3776.49	0.00	A	36.6740
22	rot	0.5813892	-		
23	rot	0.1931190	-		
24	rot	0.1509441	-		

```
--------------------------------------------------------------------------------
------------------------ ZPE and THERMAL CONTRIBUTIONS ------------------------
--------------------------------------------------------------------------------
Eelectronic                    [kJ/mol]   = -549141.992
Ezpe                           [kJ/mol]   = +184.738
Eelectronic+Ezpe               [kJ/mol]   = -548957.254

Eelectronic                    [hartrees] = -209.157110
Ezpe                           [hartrees] = +0.070363
Eelectronic+Ezpe               [hartrees] = -209.086747

Thermal Correction to Energy   [kJ/mol]   = +199.480
Thermal Correction to Enthalpy [kJ/mol]   = +201.959
Thermal Correction to Gibbs    [kJ/mol]   = +110.833
--------------------------------------------------------------------------------
```

#39 CH_3–NH–C̈–OH

Charge = 0 Multiplicity = 1 Stoichiometry C2H5NO

OPTIMIZED GEOMETRY

Center Number	Atomic Number	Atomic Type	Coordinates (Angstroms) X	Y	Z
1	6	0	-0.866438	0.698687	-0.000046
2	6	0	1.377928	-0.472603	0.000009
3	7	0	0.467359	0.679467	0.000017
4	8	0	-1.417995	-0.518712	-0.000001
5	1	0	2.400600	-0.106141	-0.002581
6	1	0	1.242919	-1.094167	-0.887925
7	1	0	1.246317	-1.091769	0.890128
8	1	0	0.895767	1.588403	0.000262
9	1	0	-0.782094	-1.259395	0.000225

A, B, C / GHz 18.4542809 6.4334075 4.9183080

```
-------------------- FREQUENCIES AND ROTATIONAL CONSTANTS ---------------------
-------------------------------------------------------------------------------
INDEX NO. DOF TYPE  CM-1(UNSCALED)  CM-1(SCALED BY ) SYMMETRY IR-INTENSITY
   1        vib          112.35          0.00              A         0.3823
   2        vib          309.85          0.00              A         3.7905
   3        vib          317.50          0.00              A        13.0538
   4        vib          607.25          0.00              A       138.5588
   5        vib          684.75          0.00              A         0.1300
   6        vib          702.83          0.00              A         1.9701
   7        vib          928.47          0.00              A         1.8782
   8        vib         1124.07          0.00              A         0.0041
   9        vib         1136.91          0.00              A        78.0271
  10        vib         1148.85          0.00              A         0.4081
  11        vib         1293.93          0.00              A         6.1810
  12        vib         1360.33          0.00              A       166.8190
  13        vib         1459.76          0.00              A        11.4173
  14        vib         1494.59          0.00              A         8.2764
  15        vib         1496.90          0.00              A        22.6263
  16        vib         1523.50          0.00              A        45.3053
  17        vib         3018.60          0.00              A        20.2007
  18        vib         3067.26          0.00              A        24.0225
  19        vib         3131.78          0.00              A         9.3932
  20        vib         3542.67          0.00              A        22.6926
  21        vib         3640.21          0.00              A        43.2021
  22        rot      0.6155686          -
  23        rot      0.2145954          -
  24        rot      0.1640571          -
-------------------------------------------------------------------------------

-------------------------------------------------------------------------------
------------------------ ZPE and THERMAL CONTRIBUTIONS ------------------------
-------------------------------------------------------------------------------
Eelectronic                    [kJ/mol]   = -549292.827
Ezpe                           [kJ/mol]   = +192.016
Eelectronic+Ezpe               [kJ/mol]   = -549100.811

Eelectronic                    [hartrees] = -209.214560
Ezpe                           [hartrees] = +0.073135
Eelectronic+Ezpe               [hartrees] = -209.141425

Thermal Correction to Energy   [kJ/mol]   = +204.855
Thermal Correction to Enthalpy [kJ/mol]   = +207.333
Thermal Correction to Gibbs    [kJ/mol]   = +120.844
-------------------------------------------------------------------------------
```

#4 2-oxiranamine

```
Charge = 0 Multiplicity = 1  Stoichiometry    C2H5NO
```

OPTIMIZED GEOMETRY

Center Number	Atomic Number	Atomic Type	Coordinates (Angstroms) X	Y	Z
1	6	0	0.196306	-0.068390	0.464592
2	6	0	-1.007645	0.606315	0.001108
3	7	0	1.468649	0.204618	-0.085542
4	8	0	-0.798783	-0.782123	-0.307082
5	1	0	-0.937300	1.284408	-0.845288
6	1	0	-1.821016	0.819488	0.687660
7	1	0	0.246979	-0.394562	1.497618
8	1	0	1.407016	0.485909	-1.055711
9	1	0	2.082072	-0.598136	-0.023033

A, B, C / GHz 19.1566184 6.9732212 6.1726644

FREQUENCIES AND ROTATIONAL CONSTANTS

INDEX NO.	DOF TYPE	CM-1(UNSCALED)	CM-1(SCALED BY)	SYMMETRY	IR-INTENSITY
1	vib	301.29	0.00	A	37.7357
2	vib	425.60	0.00	A	32.6433
3	vib	455.32	0.00	A	39.9036
4	vib	745.25	0.00	A	16.1936
5	vib	814.40	0.00	A	235.6671
6	vib	839.89	0.00	A	10.3847
7	vib	960.22	0.00	A	19.6202
8	vib	1054.36	0.00	A	19.7710
9	vib	1107.92	0.00	A	16.7802
10	vib	1147.96	0.00	A	7.4523
11	vib	1174.99	0.00	A	2.6403
12	vib	1267.95	0.00	A	22.4978
13	vib	1311.43	0.00	A	8.0545
14	vib	1466.65	0.00	A	25.2097
15	vib	1540.73	0.00	A	21.6145
16	vib	1663.78	0.00	A	36.3417
17	vib	3070.61	0.00	A	31.0018
18	vib	3131.31	0.00	A	11.8951
19	vib	3151.74	0.00	A	38.9269
20	vib	3505.49	0.00	A	2.0277
21	vib	3587.31	0.00	A	4.6978
22	rot	0.6389960	-		
23	rot	0.2326016	-		
24	rot	0.2058979	-		

```
----------------------- ZPE and THERMAL CONTRIBUTIONS ------------------------
------------------------------------------------------------------------------
Eelectronic                    [kJ/mol]    = -549337.713
Ezpe                           [kJ/mol]    = +195.734
Eelectronic+Ezpe               [kJ/mol]    = -549141.979

Eelectronic                    [hartrees] = -209.231656
Ezpe                           [hartrees] = +0.074551
Eelectronic+Ezpe               [hartrees] = -209.157105

Thermal Correction to Energy   [kJ/mol]    = +206.774
Thermal Correction to Enthalpy [kJ/mol]    = +209.255
Thermal Correction to Gibbs    [kJ/mol]    = +127.234
------------------------------------------------------------------------------
```

#40 H–C̈–N(OH)CH_3

Charge = 0 Multiplicity = 1 Stoichiometry C2H5NO

OPTIMIZED GEOMETRY

Center Number	Atomic Number	Atomic Type	Coordinates (Angstroms) X	Y	Z
1	6	0	-0.706263	1.299944	0.000016
2	6	0	1.368106	0.007594	0.000033
3	7	0	-0.075404	0.206794	-0.000081
4	8	0	-0.648385	-1.162386	-0.000044
5	1	0	-1.792395	1.134340	0.000003
6	1	0	1.835575	0.984646	-0.000033
7	1	0	1.650595	-0.558289	-0.885856
8	1	0	1.650502	-0.558103	0.886076
9	1	0	-1.600427	-0.996291	0.000435

A, B, C / GHz 10.5478988 9.8038906 5.2481133

FREQUENCIES AND ROTATIONAL CONSTANTS

INDEX NO.	DOF TYPE	CM-1(UNSCALED)	CM-1(SCALED BY)	SYMMETRY	IR-INTENSITY
1	vib	180.28	0.00	A	5.8498
2	vib	267.13	0.00	A	82.4781
3	vib	421.00	0.00	A	5.6288
4	vib	430.59	0.00	A	4.4120
5	vib	494.09	0.00	A	77.8968
6	vib	648.33	0.00	A	164.5176
7	vib	900.53	0.00	A	30.3370

8	vib	958.81	0.00	A	34.0119
9	vib	1108.95	0.00	A	46.3708
10	vib	1160.88	0.00	A	1.6265
11	vib	1195.34	0.00	A	97.3185
12	vib	1307.82	0.00	A	14.0740
13	vib	1437.97	0.00	A	1.0178
14	vib	1482.10	0.00	A	10.6454
15	vib	1494.11	0.00	A	9.5295
16	vib	1654.77	0.00	A	37.9277
17	vib	3002.83	0.00	A	49.2715
18	vib	3059.31	0.00	A	14.5948
19	vib	3122.11	0.00	A	9.9665
20	vib	3182.21	0.00	A	1.8523
21	vib	3763.16	0.00	A	26.5837
22	rot	0.3518400	-		
23	rot	0.3270226	-		
24	rot	0.1750582	-		

```
--------------------------------------------------------------------------------

--------------------------------------------------------------------------------
------------------------ ZPE and THERMAL CONTRIBUTIONS -------------------------
--------------------------------------------------------------------------------
Eelectronic                    [kJ/mol]   = -549119.555
Ezpe                           [kJ/mol]   = +187.051
Eelectronic+Ezpe               [kJ/mol]   = -548932.504

Eelectronic                    [hartrees] = -209.148564
Ezpe                           [hartrees] = +0.071244
Eelectronic+Ezpe               [hartrees] = -209.077320

Thermal Correction to Energy   [kJ/mol]   = +200.202
Thermal Correction to Enthalpy [kJ/mol]   = +202.681
Thermal Correction to Gibbs    [kJ/mol]   = +116.297
--------------------------------------------------------------------------------
```

#41 $H_2N-\ddot{C}-CH_2OH$

Charge = 0 Multiplicity = 1 Stoichiometry   C2H5NO

OPTIMIZED GEOMETRY

Center Number	Atomic Number	Atomic Type	Coordinates (Angstroms) X	Y	Z
1	6	0	0.825266	0.746866	0.050258
2	6	0	-0.665376	0.667915	-0.017384
3	7	0	1.370636	-0.434761	-0.002896

4	8	0	-1.261987	-0.647562	-0.097350
5	1	0	-1.073762	1.231011	0.826293
6	1	0	-0.962620	1.213315	-0.918311
7	1	0	0.816639	-1.289568	-0.086408
8	1	0	2.373185	-0.537759	0.014500
9	1	0	-1.611341	-0.881860	0.765755

A, B, C / GHz 17.2919984 6.4089783 4.8818649

FREQUENCIES AND ROTATIONAL CONSTANTS

INDEX NO.	DOF TYPE	CM-1(UNSCALED)	CM-1(SCALED BY)	SYMMETRY	IR-INTENSITY
1	vib	157.21	0.00	A	82.7279
2	vib	269.46	0.00	A	48.5616
3	vib	312.78	0.00	A	13.0221
4	vib	648.95	0.00	A	1.5934
5	vib	680.59	0.00	A	6.5765
6	vib	787.92	0.00	A	134.3252
7	vib	907.78	0.00	A	1.9113
8	vib	963.97	0.00	A	126.9905
9	vib	978.56	0.00	A	28.0510
10	vib	1183.65	0.00	A	27.6856
11	vib	1230.43	0.00	A	2.9771
12	vib	1334.89	0.00	A	17.7218
13	vib	1353.78	0.00	A	1.4525
14	vib	1428.50	0.00	A	23.4072
15	vib	1483.40	0.00	A	0.6499
16	vib	1641.50	0.00	A	47.8045
17	vib	3022.05	0.00	A	19.0792
18	vib	3054.45	0.00	A	11.2219
19	vib	3357.44	0.00	A	13.6836
20	vib	3592.60	0.00	A	35.5192
21	vib	3841.84	0.00	A	36.6458
22	rot	0.5767990	-		
23	rot	0.2137805	-		
24	rot	0.1628415	-		

ZPE and THERMAL CONTRIBUTIONS

Eelectronic	[kJ/mol]	= -549260.494
Ezpe	[kJ/mol]	= +192.788
Eelectronic+Ezpe	[kJ/mol]	= -549067.706
Eelectronic	[hartrees]	= -209.202245
Ezpe	[hartrees]	= +0.073429
Eelectronic+Ezpe	[hartrees]	= -209.128816

```
Thermal Correction to Energy   [kJ/mol]   = +205.564
Thermal Correction to Enthalpy [kJ/mol]   = +208.045
Thermal Correction to Gibbs    [kJ/mol]   = +121.962
--------------------------------------------------------------------------------
```

#43 $H_2N-\ddot{C}-O-CH_3$

Charge = 0 Multiplicity = 1 Stoichiometry   C2H5NO

```
---------------------------------------------------------------------
---------------------- OPTIMIZED GEOMETRY ---------------------------
---------------------------------------------------------------------
```

Center Number	Atomic Number	Atomic Type	Coordinates (Angstroms) X	Y	Z
1	6	0	-0.555394	0.542666	-0.000280
2	6	0	1.743333	0.077080	0.000228
3	7	0	-1.732672	-0.070649	0.000252
4	8	0	0.395233	-0.398005	-0.000413
5	1	0	2.246661	-0.310599	-0.885013
6	1	0	2.242379	-0.298598	0.893094
7	1	0	1.748671	1.164885	-0.006882
8	1	0	-1.832185	-1.082133	0.000569
9	1	0	-2.566318	0.486544	0.000083

A, B, C / GHz   46.9864232   4.6143622   4.3160730

```
--------------------------------------------------------------------------------
-------------------- FREQUENCIES AND ROTATIONAL CONSTANTS ----------------------
--------------------------------------------------------------------------------
```

INDEX NO.	DOF TYPE	CM-1(UNSCALED)	CM-1(SCALED BY)	SYMMETRY	IR-INTENSITY
1	vib	72.26	0.00	A	0.5102
2	vib	214.16	0.00	A	23.7398
3	vib	349.05	0.00	A	21.5968
4	vib	502.63	0.00	A	157.8730
5	vib	587.56	0.00	A	9.5058
6	vib	742.76	0.00	A	3.8235
7	vib	923.15	0.00	A	5.5832
8	vib	1145.50	0.00	A	145.7734
9	vib	1183.60	0.00	A	1.0982
10	vib	1185.64	0.00	A	12.3193
11	vib	1289.51	0.00	A	153.0959
12	vib	1357.40	0.00	A	115.2288
13	vib	1468.50	0.00	A	11.8629
14	vib	1495.13	0.00	A	3.1792
15	vib	1506.32	0.00	A	9.0608
16	vib	1629.90	0.00	A	98.1243
17	vib	3034.19	0.00	A	62.3475

18	vib	3102.07	0.00	A	29.6250
19	vib	3126.68	0.00	A	11.9052
20	vib	3443.52	0.00	A	3.0240
21	vib	3697.23	0.00	A	70.0940
22	rot	1.5672984	-		
23	rot	0.1539186	-		
24	rot	0.1439687	-		

ZPE and THERMAL CONTRIBUTIONS

```
Eelectronic                    [kJ/mol]    = -549305.12
Ezpe                           [kJ/mol]    = +191.743
Eelectronic+Ezpe               [kJ/mol]    = -549113.377

Eelectronic                    [hartrees] = -209.219242
Ezpe                           [hartrees] = +0.073031
Eelectronic+Ezpe               [hartrees] = -209.146211

Thermal Correction to Energy   [kJ/mol]    = +205.272
Thermal Correction to Enthalpy [kJ/mol]    = +207.751
Thermal Correction to Gibbs    [kJ/mol]    = +119.786
```

#44 H−C̈−NH−OCH_3

Charge = 0 Multiplicity = 1 Stoichiometry C2H5NO

OPTIMIZED GEOMETRY

Center Number	Atomic Number	Atomic Type	Coordinates (Angstroms) X	Y	Z
1	6	0	-1.871976	-0.021466	0.248691
2	6	0	1.581688	0.114965	0.240392
3	7	0	-0.742452	0.350711	-0.169745
4	8	0	0.453908	-0.499641	-0.364238
5	1	0	-1.841027	-1.081474	0.526741
6	1	0	1.754060	1.124109	-0.153225
7	1	0	2.437890	-0.505027	-0.018808
8	1	0	1.480733	0.164499	1.328192
9	1	0	-0.524031	1.279049	-0.515279

A, B, C / GHz 30.6992326 4.8617169 4.6598569

-------------------- FREQUENCIES AND ROTATIONAL CONSTANTS ---------------------

INDEX NO.	DOF TYPE	CM-1(UNSCALED)	CM-1(SCALED BY)	SYMMETRY	IR-INTENSITY
1	vib	65.40	0.00	A	1.4333
2	vib	199.25	0.00	A	0.7056
3	vib	347.88	0.00	A	5.1656
4	vib	470.73	0.00	A	105.6103
5	vib	665.23	0.00	A	34.1485
6	vib	703.59	0.00	A	143.4272
7	vib	1006.23	0.00	A	117.5112
8	vib	1057.21	0.00	A	127.6162
9	vib	1139.21	0.00	A	65.1101
10	vib	1171.39	0.00	A	2.1533
11	vib	1199.99	0.00	A	6.3978
12	vib	1394.68	0.00	A	17.8753
13	vib	1464.58	0.00	A	1.9012
14	vib	1482.10	0.00	A	8.9146
15	vib	1505.71	0.00	A	14.0382
16	vib	1570.15	0.00	A	30.5765
17	vib	2979.12	0.00	A	60.3245
18	vib	3040.29	0.00	A	49.2273
19	vib	3042.59	0.00	A	15.7524
20	vib	3113.18	0.00	A	13.8110
21	vib	3504.04	0.00	A	12.2118
22	rot	1.0240162	-		
23	rot	0.1621694	-		
24	rot	0.1554361	-		

------------------------ ZPE and THERMAL CONTRIBUTIONS ------------------------

Eelectronic	[kJ/mol]	= -549090.669
Ezpe	[kJ/mol]	= +186.153
Eelectronic+Ezpe	[kJ/mol]	= -548904.516
Eelectronic	[hartrees]	= -209.137562
Ezpe	[hartrees]	= +0.070902
Eelectronic+Ezpe	[hartrees]	= -209.066660
Thermal Correction to Energy	[kJ/mol]	= +199.795
Thermal Correction to Enthalpy	[kJ/mol]	= +202.274
Thermal Correction to Gibbs	[kJ/mol]	= +113.506

#46 H–C̈–NH–CH_2OH

Charge = 0 Multiplicity = 1 Stoichiometry C2H5NO

OPTIMIZED GEOMETRY

Center Number	Atomic Number	Atomic Type	Coordinates (Angstroms) X	Y	Z
1	6	0	1.868897	-0.029467	-0.300427
2	6	0	-0.520125	-0.377475	0.400608
3	7	0	0.730437	0.373015	0.195629
4	8	0	-1.559691	0.064630	-0.435002
5	1	0	1.738713	-1.103284	-0.544736
6	1	0	-0.800935	-0.310932	1.456193
7	1	0	-0.302444	-1.411880	0.143388
8	1	0	0.666698	1.348824	0.471245
9	1	0	-2.030192	0.790777	-0.016564

A, B, C / GHz 27.0798970 4.6483254 4.5972423

FREQUENCIES AND ROTATIONAL CONSTANTS

INDEX NO.	DOF TYPE	CM-1(UNSCALED)	CM-1(SCALED BY)	SYMMETRY	IR-INTENSITY
1	vib	64.07	0.00	A	10.8927
2	vib	243.71	0.00	A	103.3526
3	vib	384.26	0.00	A	5.1888
4	vib	498.26	0.00	A	11.5426
5	vib	658.00	0.00	A	4.4514
6	vib	938.01	0.00	A	40.2045
7	vib	1035.73	0.00	A	20.1762
8	vib	1066.67	0.00	A	206.3126
9	vib	1093.31	0.00	A	18.2770
10	vib	1206.16	0.00	A	61.1230
11	vib	1310.95	0.00	A	11.7404
12	vib	1388.35	0.00	A	15.8916
13	vib	1411.36	0.00	A	43.0043
14	vib	1466.52	0.00	A	10.1161
15	vib	1495.64	0.00	A	0.2911
16	vib	1540.29	0.00	A	16.3644
17	vib	2887.84	0.00	A	98.0604
18	vib	3009.83	0.00	A	53.1223
19	vib	3111.95	0.00	A	12.9211
20	vib	3465.41	0.00	A	5.9727
21	vib	3828.86	0.00	A	48.3890
22	rot	0.9032881	-		
23	rot	0.1550514	-		
24	rot	0.1533475	-		

------------------------ ZPE and THERMAL CONTRIBUTIONS ------------------------

Eelectronic	[kJ/mol]	= -549209.058
Ezpe	[kJ/mol]	= +192.032
Eelectronic+Ezpe	[kJ/mol]	= -549017.026
Eelectronic	[hartrees]	= -209.182654
Ezpe	[hartrees]	= +0.073141
Eelectronic+Ezpe	[hartrees]	= -209.109513
Thermal Correction to Energy	[kJ/mol]	= +205.170
Thermal Correction to Enthalpy	[kJ/mol]	= +207.651
Thermal Correction to Gibbs	[kJ/mol]	= +119.576

#5 Figure 15a

Charge = 0 Multiplicity = 1 Stoichiometry C2H5NO

--------------------- OPTIMIZED GEOMETRY -------------------------

Center Number	Atomic Number	Atomic Type	Coordinates (Angstroms) X	Y	Z
1	6	0	-0.581519	-0.448888	-0.000008
2	6	0	0.371345	0.501192	0.000007
3	7	0	1.721524	-0.137769	0.000005
4	8	0	-1.894236	-0.097063	-0.000008
5	1	0	0.390839	1.587455	0.000021
6	1	0	1.412156	-1.130913	-0.000008
7	1	0	2.288506	0.051025	0.828375
8	1	0	2.288512	0.051044	-0.828357
9	1	0	-2.015750	0.868456	0.000003

A, B, C / GHz 43.0426143 4.1242515 3.8427924

------------------- FREQUENCIES AND ROTATIONAL CONSTANTS ---------------------

INDEX NO.	DOF TYPE	CM-1(UNSCALED)	CM-1(SCALED BY)	SYMMETRY	IR-INTENSITY
1	vib	252.50	0.00	A	58.0323
2	vib	301.89	0.00	A	88.2824
3	vib	378.74	0.00	A	29.4702
4	vib	527.12	0.00	A	9.5829
5	vib	534.81	0.00	A	1.6535
6	vib	645.31	0.00	A	93.7235
7	vib	891.35	0.00	A	0.2328

8	vib	1013.12	0.00	A	0.4648
9	vib	1067.42	0.00	A	5.3776
10	vib	1104.39	0.00	A	100.3752
11	vib	1238.27	0.00	A	93.7691
12	vib	1320.72	0.00	A	4.5273
13	vib	1365.79	0.00	A	147.6443
14	vib	1510.33	0.00	A	39.8165
15	vib	1610.56	0.00	A	16.5456
16	vib	1642.93	0.00	A	16.8005
17	vib	3103.51	0.00	A	61.8325
18	vib	3137.59	0.00	A	160.2336
19	vib	3381.45	0.00	A	8.0938
20	vib	3419.77	0.00	A	13.8187
21	vib	3567.56	0.00	A	67.3999
22	rot	1.4357471	-		
23	rot	0.1375702	-		
24	rot	0.1281818	-		

```
--------------------------------------------------------------------------------

--------------------------------------------------------------------------------
----------------------- ZPE and THERMAL CONTRIBUTIONS ---------------------------
--------------------------------------------------------------------------------
Eelectronic                    [kJ/mol]   = -549119.809
Ezpe                           [kJ/mol]   = +191.493
Eelectronic+Ezpe               [kJ/mol]   = -548928.316

Eelectronic                    [hartrees] = -209.148661
Ezpe                           [hartrees] = +0.072936
Eelectronic+Ezpe               [hartrees] = -209.075725

Thermal Correction to Energy   [kJ/mol]   = +204.075
Thermal Correction to Enthalpy [kJ/mol]   = +206.553
Thermal Correction to Gibbs    [kJ/mol]   = +121.726
--------------------------------------------------------------------------------
```

#51 N-methyl-formamide

Charge = 0 Multiplicity = 1 Stoichiometry   C2H5NO

```
----------------------------------------------------------------------
---------------------- OPTIMIZED GEOMETRY ----------------------------
----------------------------------------------------------------------
```

Center Number	Atomic Number	Atomic Type	Coordinates (Angstroms) X	Y	Z
1	6	0	-1.451949	-0.431750	0.000019
2	6	0	0.866168	0.426163	-0.000034
3	7	0	-0.473590	0.640457	-0.000089

4	8	0	1.408394	-0.658767	0.000016
5	1	0	-2.080047	-0.392409	0.891338
6	1	0	1.433421	1.373519	0.000242
7	1	0	-0.791729	1.594209	0.000673
8	1	0	-2.090041	-0.382384	-0.883574
9	1	0	-0.908942	-1.372478	-0.008101

A, B, C / GHz 20.1092105 6.0935137 4.8180080

FREQUENCIES AND ROTATIONAL CONSTANTS

INDEX NO.	DOF TYPE	CM-1(UNSCALED)	CM-1(SCALED BY)	SYMMETRY	IR-INTENSITY
1	vib	66.73	0.00	A	0.0826
2	vib	269.36	0.00	A	65.3388
3	vib	295.94	0.00	A	13.5206
4	vib	537.56	0.00	A	39.1007
5	vib	768.62	0.00	A	0.6517
6	vib	951.93	0.00	A	17.2895
7	vib	1022.28	0.00	A	0.0035
8	vib	1154.30	0.00	A	0.8934
9	vib	1161.15	0.00	A	22.8437
10	vib	1218.41	0.00	A	82.4316
11	vib	1422.49	0.00	A	5.4770
12	vib	1443.62	0.00	A	20.2832
13	vib	1493.19	0.00	A	33.3830
14	vib	1502.88	0.00	A	6.4416
15	vib	1555.93	0.00	A	91.8433
16	vib	1781.88	0.00	A	325.4093
17	vib	2935.19	0.00	A	115.7132
18	vib	3028.74	0.00	A	44.6153
19	vib	3076.04	0.00	A	29.1941
20	vib	3143.31	0.00	A	0.9929
21	vib	3631.65	0.00	A	22.6070
22	rot	0.6707711	-		
23	rot	0.2032577	-		
24	rot	0.1607114	-		

ZPE and THERMAL CONTRIBUTIONS

Eelectronic	[kJ/mol]	= -549483.859
Ezpe	[kJ/mol]	= +194.161
Eelectronic+Ezpe	[kJ/mol]	= -549289.698
Eelectronic	[hartrees]	= -209.287320
Ezpe	[hartrees]	= +0.073952
Eelectronic+Ezpe	[hartrees]	= -209.213368

Thermal Correction to Energy [kJ/mol] = +207.197
Thermal Correction to Enthalpy [kJ/mol] = +209.678
Thermal Correction to Gibbs [kJ/mol] = +121.784

#52 Figure 15j

Charge = 0 Multiplicity = 1 Stoichiometry C2H5NO

OPTIMIZED GEOMETRY

Center Number	Atomic Number	Atomic Type	Coordinates (Angstroms) X	Y	Z
1	6	0	1.320282	-0.393653	-0.017033
2	6	0	-1.052372	-0.865609	0.045925
3	7	0	0.038476	-0.103197	-0.009242
4	8	0	-0.208607	1.293253	-0.062924
5	1	0	1.604403	-1.397163	-0.276813
6	1	0	-1.926813	-0.523332	-0.481425
7	1	0	-0.915298	-1.895069	0.326261
8	1	0	2.016030	0.353387	0.317380
9	1	0	-0.986261	1.394102	0.509331

A, B, C / GHz 10.8727464 10.5170096 5.4443874

FREQUENCIES AND ROTATIONAL CONSTANTS

INDEX NO.	DOF TYPE	CM-1(UNSCALED)	CM-1(SCALED BY)	SYMMETRY	IR-INTENSITY
1	vib	392.91	0.00	A	11.5470
2	vib	429.21	0.00	A	108.5356
3	vib	512.88	0.00	A	4.8584
4	vib	517.48	0.00	A	30.4160
5	vib	569.82	0.00	A	110.8011
6	vib	583.49	0.00	A	126.5943
7	vib	623.54	0.00	A	53.7688
8	vib	673.05	0.00	A	32.8650
9	vib	826.31	0.00	A	58.3113
10	vib	1084.84	0.00	A	16.9439
11	vib	1098.22	0.00	A	8.7985
12	vib	1300.90	0.00	A	41.9159
13	vib	1347.75	0.00	A	87.8008
14	vib	1447.58	0.00	A	9.4911
15	vib	1488.21	0.00	A	10.4303
16	vib	1665.95	0.00	A	282.5780
17	vib	3172.99	0.00	A	2.9226

18	vib	3196.27	0.00	A	5.4024
19	vib	3295.73	0.00	A	2.2599
20	vib	3321.84	0.00	A	0.1941
21	vib	3684.02	0.00	A	10.6147
22	rot	0.3626758	-		
23	rot	0.3508097	-		
24	rot	0.1816052	-		

ZPE and THERMAL CONTRIBUTIONS

Eelectronic	[kJ/mol]	= -549114.761
Ezpe	[kJ/mol]	= +186.815
Eelectronic+Ezpe	[kJ/mol]	= -548927.946
Eelectronic	[hartrees]	= -209.146738
Ezpe	[hartrees]	= +0.071154
Eelectronic+Ezpe	[hartrees]	= -209.075584
Thermal Correction to Energy	[kJ/mol]	= +198.976
Thermal Correction to Enthalpy	[kJ/mol]	= +201.455
Thermal Correction to Gibbs	[kJ/mol]	= +117.709

#54 HO−C̈−CH_2−NH_2

Charge = 0 Multiplicity = 1 Stoichiometry C2H5NO

OPTIMIZED GEOMETRY

Center Number	Atomic Number	Atomic Type	Coordinates (Angstroms) X	Y	Z
1	6	0	-0.643635	-0.578962	0.191018
2	6	0	0.466248	0.434842	0.155903
3	7	0	1.803218	-0.062983	-0.134443
4	8	0	-1.794893	-0.054025	-0.116735
5	1	0	0.463811	0.870745	1.165759
6	1	0	0.231140	1.266267	-0.534173
7	1	0	2.057752	-0.765638	0.550464
8	1	0	1.801737	-0.537678	-1.030175
9	1	0	-1.753498	0.904107	-0.358424

A, B, C / GHz 34.4151345 4.2155319 4.0155895

```
-------------------- FREQUENCIES AND ROTATIONAL CONSTANTS ---------------------
-------------------------------------------------------------------------------
INDEX NO. DOF TYPE  CM-1(UNSCALED)  CM-1(SCALED BY ) SYMMETRY IR-INTENSITY
   1        vib           77.52           0.00              A        5.7934
   2        vib          210.90           0.00              A       79.7206
   3        vib          342.03           0.00              A       69.7291
   4        vib          522.70           0.00              A        3.5080
   5        vib          682.23           0.00              A       38.6986
   6        vib          878.75           0.00              A      112.4156
   7        vib          894.71           0.00              A       41.0109
   8        vib          970.15           0.00              A       37.1332
   9        vib         1129.20           0.00              A        1.1492
  10        vib         1134.33           0.00              A        6.3563
  11        vib         1312.86           0.00              A       60.2647
  12        vib         1331.89           0.00              A       71.6940
  13        vib         1364.26           0.00              A       43.2619
  14        vib         1384.18           0.00              A       55.5899
  15        vib         1413.78           0.00              A        4.2346
  16        vib         1670.63           0.00              A       22.7675
  17        vib         2891.35           0.00              A       78.4473
  18        vib         2982.87           0.00              A       13.2531
  19        vib         3311.95           0.00              A      109.3975
  20        vib         3486.19           0.00              A        0.3787
  21        vib         3559.28           0.00              A        2.1325
  22        rot       1.1479653           -
  23        rot       0.1406150           -
  24        rot       0.1339456           -
-------------------------------------------------------------------------------

-------------------------------------------------------------------------------
------------------------ ZPE and THERMAL CONTRIBUTIONS ------------------------
-------------------------------------------------------------------------------
Eelectronic                     [kJ/mol]   = -549183.074
Ezpe                            [kJ/mol]   = +188.721
Eelectronic+Ezpe                [kJ/mol]   = -548994.353

Eelectronic                     [hartrees] = -209.172757
Ezpe                            [hartrees] = +0.071880
Eelectronic+Ezpe                [hartrees] = -209.100877

Thermal Correction to Energy    [kJ/mol]   = +202.108
Thermal Correction to Enthalpy  [kJ/mol]   = +204.587
Thermal Correction to Gibbs     [kJ/mol]   = +116.367
-------------------------------------------------------------------------------
```

#58 Figure 15k

```
Charge = 0 Multiplicity = 1  Stoichiometry    C2H5NO
```

----------------------- OPTIMIZED GEOMETRY ---------------------------

Center Number	Atomic Number	Atomic Type	Coordinates (Angstroms) X	Y	Z
1	6	0	1.460732	0.494475	-0.115409
2	6	0	-0.621063	-0.621954	0.034411
3	7	0	-1.341053	0.532659	-0.077537
4	8	0	0.653210	-0.611126	0.020682
5	1	0	2.447351	0.257008	0.252043
6	1	0	-1.103082	-1.583557	-0.015699
7	1	0	-2.326980	0.451747	0.108060
8	1	0	-0.919547	1.364627	0.314205
9	1	0	1.025935	1.435444	0.204682

A, B, C / GHz 20.5098700 6.5343526 4.9982893

-------------------- FREQUENCIES AND ROTATIONAL CONSTANTS ----------------------

INDEX NO.	DOF TYPE	CM-1(UNSCALED)	CM-1(SCALED BY)	SYMMETRY	IR-INTENSITY
1	vib	185.40	0.00	A	127.7461
2	vib	308.89	0.00	A	1.2925
3	vib	380.13	0.00	A	10.6015
4	vib	460.41	0.00	A	250.0262
5	vib	545.11	0.00	A	54.6033
6	vib	616.99	0.00	A	22.9475
7	vib	640.62	0.00	A	154.0560
8	vib	673.77	0.00	A	126.0867
9	vib	995.87	0.00	A	7.1935
10	vib	1077.15	0.00	A	67.8656
11	vib	1244.10	0.00	A	2.4867
12	vib	1275.98	0.00	A	71.6452
13	vib	1402.05	0.00	A	14.3136
14	vib	1480.93	0.00	A	7.3210
15	vib	1600.65	0.00	A	14.9374
16	vib	1646.52	0.00	A	122.1893
17	vib	3089.41	0.00	A	32.4832
18	vib	3225.56	0.00	A	24.9424
19	vib	3242.88	0.00	A	14.8553
20	vib	3511.46	0.00	A	4.8506
21	vib	3655.43	0.00	A	54.2484
22	rot	0.6841356	-		
23	rot	0.2179625	-		
24	rot	0.1667250	-		

```
------------------------ ZPE and THERMAL CONTRIBUTIONS ------------------------
-------------------------------------------------------------------------------
Eelectronic                    [kJ/mol]   = -549166.317
Ezpe                           [kJ/mol]   = +186.972
Eelectronic+Ezpe               [kJ/mol]   = -548979.345

Eelectronic                    [hartrees] = -209.166375
Ezpe                           [hartrees] = +0.071214
Eelectronic+Ezpe               [hartrees] = -209.095161

Thermal Correction to Energy   [kJ/mol]   = +200.431
Thermal Correction to Enthalpy [kJ/mol]   = +202.909
Thermal Correction to Gibbs    [kJ/mol]   = +116.520
-------------------------------------------------------------------------------
```

#59 Figure 15l

Charge = 0 Multiplicity = 1 Stoichiometry C2H5NO

```
---------------------------------------------------------------------
---------------------- OPTIMIZED GEOMETRY --------------------------
---------------------------------------------------------------------
```

Center Number	Atomic Number	Atomic Type	Coordinates (Angstroms) X	Y	Z
1	6	0	1.424781	0.079208	0.177387
2	6	0	-1.224017	0.727517	-0.081199
3	7	0	-0.852766	-0.620099	0.356694
4	8	0	0.207801	-0.122004	-0.563135
5	1	0	1.900458	-0.889044	0.324805
6	1	0	2.044346	0.726827	-0.439684
7	1	0	-0.727280	1.366882	0.672520
8	1	0	-1.328206	-1.278231	-0.261203
9	1	0	1.213054	0.549949	1.134657

A, B, C / GHz 19.0264740 6.8370916 6.1531051

```
-------------------------------------------------------------------------------
-------------------- FREQUENCIES AND ROTATIONAL CONSTANTS ---------------------
-------------------------------------------------------------------------------
```

INDEX NO.	DOF TYPE	CM-1(UNSCALED)	CM-1(SCALED BY)	SYMMETRY	IR-INTENSITY
1	vib	129.48	0.00	A	1.8569
2	vib	271.55	0.00	A	11.4349
3	vib	319.72	0.00	A	8.6145
4	vib	413.57	0.00	A	3.8023
5	vib	753.05	0.00	A	6.2988
6	vib	951.98	0.00	A	17.9890
7	vib	1005.60	0.00	A	62.0641

8	vib	1050.66	0.00	A	75.0131
9	vib	1146.89	0.00	A	20.6428
10	vib	1182.91	0.00	A	11.6830
11	vib	1190.08	0.00	A	8.8823
12	vib	1368.10	0.00	A	39.4218
13	vib	1395.88	0.00	A	4.8629
14	vib	1456.83	0.00	A	6.5591
15	vib	1480.54	0.00	A	6.8928
16	vib	1485.91	0.00	A	8.0957
17	vib	2893.42	0.00	A	109.8225
18	vib	3043.20	0.00	A	27.8481
19	vib	3128.77	0.00	A	11.6747
20	vib	3138.54	0.00	A	8.7681
21	vib	3415.59	0.00	A	2.6944
22	rot	0.6346549	-		
23	rot	0.2280608	-		
24	rot	0.2052455	-		

```
-------------------------------------------------------------------------------

-------------------------------------------------------------------------------
----------------------- ZPE and THERMAL CONTRIBUTIONS ------------------------
-------------------------------------------------------------------------------
Eelectronic                    [kJ/mol]   = -548879.498
Ezpe                           [kJ/mol]   = +186.752
Eelectronic+Ezpe               [kJ/mol]   = -548692.746

Eelectronic                    [hartrees] = -209.057131
Ezpe                           [hartrees] = +0.071130
Eelectronic+Ezpe               [hartrees] = -208.986001

Thermal Correction to Energy   [kJ/mol]   = +199.756
Thermal Correction to Enthalpy [kJ/mol]   = +202.237
Thermal Correction to Gibbs    [kJ/mol]   = +115.947
-------------------------------------------------------------------------------
```

#6 N-hydroxy-ethenamine

Charge = 0 Multiplicity = 1 Stoichiometry   C2H5NO

```
---------------------------------------------------------------------
--------------------- OPTIMIZED GEOMETRY --------------------------
---------------------------------------------------------------------
```

Center Number	Atomic Number	Atomic Type	Coordinates (Angstroms) X	Y	Z
1	6	0	-1.477669	-0.539280	-0.004319
2	6	0	-0.730751	0.559891	0.003154
3	7	0	0.660144	0.640990	0.135668

4	8	0	1.324976	-0.587059	-0.138758
5	1	0	-2.552654	-0.455986	-0.040396
6	1	0	-1.175585	1.548699	-0.031786
7	1	0	-1.038196	-1.524091	0.021816
8	1	0	1.064758	1.330485	-0.486928
9	1	0	1.731375	-0.813237	0.704670

A, B, C / GHz 18.5667767 6.2240864 4.7701222

-------------------- FREQUENCIES AND ROTATIONAL CONSTANTS ----------------------

INDEX NO.	DOF TYPE	CM-1(UNSCALED)	CM-1(SCALED BY)	SYMMETRY	IR-INTENSITY
1	vib	254.40	0.00	A	1.3317
2	vib	304.01	0.00	A	3.5143
3	vib	317.77	0.00	A	75.3234
4	vib	610.88	0.00	A	43.9594
5	vib	677.26	0.00	A	78.2010
6	vib	772.01	0.00	A	130.8712
7	vib	865.50	0.00	A	55.8269
8	vib	979.55	0.00	A	36.7125
9	vib	996.44	0.00	A	16.8513
10	vib	1018.07	0.00	A	5.9553
11	vib	1168.69	0.00	A	30.9414
12	vib	1325.52	0.00	A	0.8944
13	vib	1377.64	0.00	A	57.7575
14	vib	1444.66	0.00	A	4.7583
15	vib	1480.80	0.00	A	7.7782
16	vib	1711.80	0.00	A	83.2422
17	vib	3149.25	0.00	A	8.5457
18	vib	3171.28	0.00	A	0.9261
19	vib	3257.50	0.00	A	5.1679
20	vib	3506.56	0.00	A	7.8986
21	vib	3792.63	0.00	A	50.5938
22	rot	0.6193210	-		
23	rot	0.2076132	-		
24	rot	0.1591141	-		

------------------------ ZPE and THERMAL CONTRIBUTIONS -------------------------

Eelectronic	[kJ/mol]	= -549248.803
Ezpe	[kJ/mol]	= +192.491
Eelectronic+Ezpe	[kJ/mol]	= -549056.312
Eelectronic	[hartrees]	= -209.197792
Ezpe	[hartrees]	= +0.073316
Eelectronic+Ezpe	[hartrees]	= -209.124476

```
Thermal Correction to Energy   [kJ/mol]   = +204.868
Thermal Correction to Enthalpy [kJ/mol]   = +207.346
Thermal Correction to Gibbs    [kJ/mol]   = +122.508
---------------------------------------------------------------------------
```

#60 Figure 15m

Charge = 0 Multiplicity = 1 Stoichiometry   C2H5NO

```
-----------------------------------------------------------------
---------------------- OPTIMIZED GEOMETRY -----------------------
-----------------------------------------------------------------
Center     Atomic      Atomic            Coordinates (Angstroms)
Number     Number       Type              X           Y           Z
-----------------------------------------------------------------
    1          6           0         0.634711   -0.547786    0.195625
    2          6           0         1.806102    0.007017   -0.108781
    3          7           0        -0.480108    0.480636    0.123435
    4          8           0        -1.700452   -0.198058   -0.155773
    5          1           0         1.966729    1.054488   -0.380628
    6          1           0         2.699000   -0.604813   -0.092966
    7          1           0        -0.386478    1.192799   -0.608510
    8          1           0        -0.602615    0.939923    1.025743
    9          1           0        -1.357139   -1.117769   -0.082561
-----------------------------------------------------------------
A, B, C / GHz         33.0901035          4.6032611          4.2292622

---------------------------------------------------------------------------
-------------------- FREQUENCIES AND ROTATIONAL CONSTANTS ----------------------
---------------------------------------------------------------------------
INDEX NO. DOF TYPE  CM-1(UNSCALED)  CM-1(SCALED BY ) SYMMETRY IR-INTENSITY
   1        vib          76.55          0.00              A        2.6477
   2        vib         308.41          0.00              A       19.8722
   3        vib         498.01          0.00              A       19.8069
   4        vib         559.08          0.00              A      106.0142
   5        vib         634.07          0.00              A        6.2526
   6        vib         688.93          0.00              A        3.7945
   7        vib         909.36          0.00              A       40.0856
   8        vib         951.95          0.00              A       28.3699
   9        vib         978.81          0.00              A       16.1110
  10        vib        1075.23          0.00              A        8.3157
  11        vib        1245.92          0.00              A       14.1691
  12        vib        1284.47          0.00              A       38.7655
  13        vib        1415.96          0.00              A        5.6927
  14        vib        1489.61          0.00              A      117.9697
  15        vib        1620.51          0.00              A       18.3835
  16        vib        1634.35          0.00              A        9.8400
  17        vib        3012.79          0.00              A       68.6308
```

18	vib	3174.15	0.00	A	10.5843
19	vib	3324.08	0.00	A	3.6506
20	vib	3445.91	0.00	A	26.5263
21	vib	3459.34	0.00	A	119.0915
22	rot	1.1037670	-		
23	rot	0.1535483	-		
24	rot	0.1410730	-		

ZPE and THERMAL CONTRIBUTIONS

Eelectronic	[kJ/mol]	= -549012.269
Ezpe	[kJ/mol]	= +190.131
Eelectronic+Ezpe	[kJ/mol]	= -548822.138
Eelectronic	[hartrees]	= -209.107701
Ezpe	[hartrees]	= +0.072417
Eelectronic+Ezpe	[hartrees]	= -209.035284
Thermal Correction to Energy	[kJ/mol]	= +202.996
Thermal Correction to Enthalpy	[kJ/mol]	= +205.474
Thermal Correction to Gibbs	[kJ/mol]	= +118.589

#69 Figure 15n

Charge = 0 Multiplicity = 1 Stoichiometry C2H5NO

OPTIMIZED GEOMETRY

Center Number	Atomic Number	Atomic Type	Coordinates (Angstroms) X	Y	Z
1	6	0	-2.097347	0.189229	-0.009052
2	6	0	0.502102	0.537256	0.016937
3	7	0	-0.764008	-0.379246	-0.026236
4	8	0	1.687067	-0.190019	-0.109633
5	1	0	0.400724	1.116726	0.932899
6	1	0	0.395817	1.183263	-0.849117
7	1	0	-0.844001	-0.867581	0.877829
8	1	0	-0.525493	-1.141147	-0.703635
9	1	0	1.995945	-0.475293	0.755438

A, B, C / GHz 33.5443144 4.2663460 4.0020678

```
-------------------- FREQUENCIES AND ROTATIONAL CONSTANTS ---------------------
-------------------------------------------------------------------------------
INDEX NO. DOF TYPE  CM-1(UNSCALED)  CM-1(SCALED BY ) SYMMETRY IR-INTENSITY
   1        vib         109.18          0.00              A       48.1185
   2        vib         259.06          0.00              A       32.2999
   3        vib         354.23          0.00              A      114.1004
   4        vib         394.01          0.00              A        0.7441
   5        vib         572.48          0.00              A       24.9511
   6        vib         707.87          0.00              A       11.4008
   7        vib         857.58          0.00              A       13.4471
   8        vib         959.49          0.00              A       17.1733
   9        vib        1063.66          0.00              A      207.8317
  10        vib        1071.62          0.00              A        0.4530
  11        vib        1217.39          0.00              A       43.5811
  12        vib        1304.28          0.00              A        6.0264
  13        vib        1358.33          0.00              A       18.0495
  14        vib        1390.87          0.00              A       18.5969
  15        vib        1467.53          0.00              A       41.0995
  16        vib        1493.77          0.00              A        6.1794
  17        vib        2900.18          0.00              A      255.6710
  18        vib        3069.76          0.00              A       18.1503
  19        vib        3155.61          0.00              A       10.2596
  20        vib        3355.14          0.00              A       46.8752
  21        vib        3819.43          0.00              A       55.6872
  22        rot      1.1189179          -
  23        rot      0.1423100          -
  24        rot      0.1334946          -
-------------------------------------------------------------------------------

-------------------------------------------------------------------------------
----------------------- ZPE and THERMAL CONTRIBUTIONS ------------------------
-------------------------------------------------------------------------------
Eelectronic                    [kJ/mol]   = -548763.474
Ezpe                           [kJ/mol]   = +184.712
Eelectronic+Ezpe               [kJ/mol]   = -548578.762

Eelectronic                    [hartrees] = -209.012940
Ezpe                           [hartrees] = +0.070353
Eelectronic+Ezpe               [hartrees] = -208.942587

Thermal Correction to Energy   [kJ/mol]   = +198.359
Thermal Correction to Enthalpy [kJ/mol]   = +200.838
Thermal Correction to Gibbs    [kJ/mol]   = +112.991
-------------------------------------------------------------------------------
```

#77 Figure 15o

Charge = 0 Multiplicity = 1 Stoichiometry   C2H5NO

OPTIMIZED GEOMETRY

Center Number	Atomic Number	Atomic Type	Coordinates (Angstroms) X	Y	Z
1	6	0	1.576948	-0.639695	-0.135120
2	6	0	0.818139	0.539735	0.128553
3	7	0	-0.728317	0.500968	-0.150198
4	8	0	-1.280715	-0.683372	0.128198
5	1	0	0.833719	-1.450396	-0.248752
6	1	0	1.234967	1.505491	-0.171701
7	1	0	0.892860	0.461437	1.233046
8	1	0	-1.143674	1.272911	0.392641
9	1	0	-0.844460	0.770520	-1.140028

A, B, C / GHz 16.7464126 6.2623874 4.9183681

FREQUENCIES AND ROTATIONAL CONSTANTS

INDEX NO.	DOF TYPE	CM-1(UNSCALED)	CM-1(SCALED BY)	SYMMETRY	IR-INTENSITY
1	vib	123.45	0.00	A	27.7248
2	vib	279.79	0.00	A	26.9927
3	vib	431.17	0.00	A	12.0304
4	vib	546.07	0.00	A	22.0065
5	vib	618.66	0.00	A	2.3876
6	vib	762.60	0.00	A	66.7543
7	vib	950.91	0.00	A	42.0112
8	vib	1017.69	0.00	A	43.1860
9	vib	1061.82	0.00	A	18.2564
10	vib	1135.32	0.00	A	20.9029
11	vib	1218.71	0.00	A	9.2287
12	vib	1275.67	0.00	A	14.5505
13	vib	1300.45	0.00	A	25.6985
14	vib	1338.02	0.00	A	43.1518
15	vib	1402.87	0.00	A	5.4007
16	vib	1651.48	0.00	A	3.0401
17	vib	2921.38	0.00	A	19.5585
18	vib	2962.40	0.00	A	5.4184
19	vib	3042.87	0.00	A	23.5453
20	vib	3202.76	0.00	A	41.2018
21	vib	3224.12	0.00	A	7.0340
22	rot	0.5586002	-		
23	rot	0.2088908	-		
24	rot	0.1640591	-		

```
----------------------- ZPE and THERMAL CONTRIBUTIONS ------------------------
------------------------------------------------------------------------------
Eelectronic                    [kJ/mol]   = -548820.985
Ezpe                           [kJ/mol]   = +182.241
Eelectronic+Ezpe               [kJ/mol]   = -548638.744

Eelectronic                    [hartrees] = -209.034845
Ezpe                           [hartrees] = +0.069412
Eelectronic+Ezpe               [hartrees] = -208.965433

Thermal Correction to Energy   [kJ/mol]   = +195.027
Thermal Correction to Enthalpy [kJ/mol]   = +197.506
Thermal Correction to Gibbs    [kJ/mol]   = +111.166
------------------------------------------------------------------------------
```

#79 Figure 15p

Charge = 0 Multiplicity = 1 Stoichiometry   C2H5NO

```
---------------------------------------------------------------------
--------------------- OPTIMIZED GEOMETRY --------------------------
---------------------------------------------------------------------
Center     Atomic      Atomic             Coordinates (Angstroms)
Number     Number       Type             X           Y           Z
---------------------------------------------------------------------
     1          6           0       -0.675721    0.855217    0.000102
     2          6           0       -1.427143   -0.380804   -0.000193
     3          7           0        0.759334    0.485814   -0.000033
     4          8           0        1.218143   -0.763772    0.000038
     5          1           0       -2.503042   -0.247094   -0.001239
     6          1           0       -1.067218   -1.000246   -0.840766
     7          1           0       -1.069235   -0.997598    0.843311
     8          1           0        1.098597    1.054136    0.805519
     9          1           0        1.097602    1.053806   -0.806355
---------------------------------------------------------------------
A, B, C / GHz         17.0447868           6.6528057           5.0468221
```

```
------------------------------------------------------------------------------
-------------------- FREQUENCIES AND ROTATIONAL CONSTANTS ---------------------
------------------------------------------------------------------------------
INDEX NO. DOF TYPE  CM-1(UNSCALED)  CM-1(SCALED BY ) SYMMETRY IR-INTENSITY
   1        vib         127.26         0.00               A         3.6984
   2        vib         186.95         0.00               A        47.2864
   3        vib         308.82         0.00               A        29.4832
   4        vib         619.25         0.00               A        11.7454
   5        vib         631.18         0.00               A         0.0086
   6        vib         742.00         0.00               A         8.5395
   7        vib         805.66         0.00               A         2.1099
```

8	vib	1015.24	0.00	A	63.9424
9	vib	1048.94	0.00	A	42.1000
10	vib	1148.51	0.00	A	28.3125
11	vib	1214.97	0.00	A	14.8401
12	vib	1271.36	0.00	A	65.8084
13	vib	1303.11	0.00	A	60.3219
14	vib	1398.76	0.00	A	21.3430
15	vib	1434.57	0.00	A	7.5314
16	vib	1528.66	0.00	A	20.2553
17	vib	2938.23	0.00	A	10.2404
18	vib	2942.91	0.00	A	0.4608
19	vib	3128.63	0.00	A	36.0092
20	vib	3131.17	0.00	A	15.5309
21	vib	3155.24	0.00	A	9.1515
22	rot	0.5685529	-		
23	rot	0.2219137	-		
24	rot	0.1683439	-		

```
--------------------------------------------------------------------------------

--------------------------------------------------------------------------------
------------------------ ZPE and THERMAL CONTRIBUTIONS -------------------------
--------------------------------------------------------------------------------
Eelectronic                    [kJ/mol]   = -548885.576
Ezpe                           [kJ/mol]   = +179.926
Eelectronic+Ezpe               [kJ/mol]   = -548705.650

Eelectronic                    [hartrees] = -209.059446
Ezpe                           [hartrees] = +0.068530
Eelectronic+Ezpe               [hartrees] = -208.990916

Thermal Correction to Energy   [kJ/mol]   = +193.363
Thermal Correction to Enthalpy [kJ/mol]   = +195.841
Thermal Correction to Gibbs    [kJ/mol]   = +108.218
--------------------------------------------------------------------------------
```

#8 Z 2-amino-ethenol

Charge = 0 Multiplicity = 1 Stoichiometry   C2H5NO

OPTIMIZED GEOMETRY

Center Number	Atomic Number	Atomic Type	Coordinates (Angstroms) X	Y	Z
1	6	0	-0.575379	0.699459	-0.000000
2	6	0	0.752953	0.585561	0.000001
3	7	0	-1.349236	-0.512339	-0.000000

4	8	0	1.400782	-0.600562	-0.000000
5	1	0	-1.046201	1.675217	-0.000001
6	1	0	1.418925	1.436724	0.000001
7	1	0	-1.947501	-0.579852	0.814871
8	1	0	0.695244	-1.271495	-0.000004
9	1	0	-1.947511	-0.579846	-0.814864

A, B, C / GHz 18.0964688 6.3036178 4.7938224

FREQUENCIES AND ROTATIONAL CONSTANTS

INDEX NO.	DOF TYPE	CM-1(UNSCALED)	CM-1(SCALED BY)	SYMMETRY	IR-INTENSITY
1	vib	197.11	0.00	A	16.9927
2	vib	267.34	0.00	A	3.6363
3	vib	481.74	0.00	A	0.0000
4	vib	702.91	0.00	A	141.2077
5	vib	735.88	0.00	A	68.0399
6	vib	752.32	0.00	A	0.2139
7	vib	883.12	0.00	A	74.1197
8	vib	953.64	0.00	A	7.4076
9	vib	1018.07	0.00	A	22.3498
10	vib	1121.62	0.00	A	127.9884
11	vib	1200.29	0.00	A	3.3242
12	vib	1234.60	0.00	A	87.2743
13	vib	1372.40	0.00	A	5.0837
14	vib	1428.77	0.00	A	43.7776
15	vib	1634.39	0.00	A	13.7260
16	vib	1730.10	0.00	A	99.6606
17	vib	3144.58	0.00	A	19.6393
18	vib	3205.40	0.00	A	16.2805
19	vib	3485.92	0.00	A	0.4391
20	vib	3546.76	0.00	A	6.6037
21	vib	3633.87	0.00	A	64.5731
22	rot	0.6036332	-		
23	rot	0.2102661	-		
24	rot	0.1599047	-		

ZPE and THERMAL CONTRIBUTIONS

Eelectronic	[kJ/mol]	= -549396.773
Ezpe	[kJ/mol]	= +195.773
Eelectronic+Ezpe	[kJ/mol]	= -549201.000
Eelectronic	[hartrees]	= -209.254151
Ezpe	[hartrees]	= +0.074566
Eelectronic+Ezpe	[hartrees]	= -209.179585

Thermal Correction to Energy [kJ/mol] = +207.882
Thermal Correction to Enthalpy [kJ/mol] = +210.363
Thermal Correction to Gibbs [kJ/mol] = +125.704

#83 aziridine N-oxide

Charge = 0 Multiplicity = 1 Stoichiometry C2H5NO

OPTIMIZED GEOMETRY

Center Number	Atomic Number	Atomic Type	Coordinates (Angstroms) X	Y	Z
1	6	0	0.858179	-0.745010	-0.141695
2	6	0	0.855792	0.745894	-0.141627
3	7	0	-0.317475	-0.000077	0.410090
4	8	0	-1.483192	-0.000831	-0.204529
5	1	0	1.481533	-1.272964	0.566929
6	1	0	0.594663	-1.261912	-1.050384
7	1	0	1.478515	1.276251	0.565753
8	1	0	0.590804	1.261343	-1.050736
9	1	0	-0.341482	-0.000841	1.433965

A, B, C / GHz 17.8798095 7.4682673 6.3936097

FREQUENCIES AND ROTATIONAL CONSTANTS

INDEX NO.	DOF TYPE	CM-1(UNSCALED)	CM-1(SCALED BY)	SYMMETRY	IR-INTENSITY
1	vib	409.71	0.00	A	5.5757
2	vib	426.24	0.00	A	14.8442
3	vib	679.28	0.00	A	23.2803
4	vib	755.30	0.00	A	2.6169
5	vib	818.27	0.00	A	1.4672
6	vib	919.88	0.00	A	1.0088
7	vib	1019.91	0.00	A	55.1834
8	vib	1099.62	0.00	A	8.4921
9	vib	1101.76	0.00	A	61.2077
10	vib	1128.70	0.00	A	3.3251
11	vib	1154.34	0.00	A	45.0661
12	vib	1207.56	0.00	A	2.2749
13	vib	1227.20	0.00	A	10.1090
14	vib	1431.05	0.00	A	7.2269
15	vib	1454.72	0.00	A	5.0470
16	vib	1482.33	0.00	A	2.8228
17	vib	3132.33	0.00	A	8.3512

18	vib	3136.88	0.00	A	6.4665
19	vib	3243.27	0.00	A	1.3379
20	vib	3254.91	0.00	A	0.0287
21	vib	3309.30	0.00	A	22.5367
22	rot	0.5964062	-		
23	rot	0.2491146	-		
24	rot	0.2132679	-		

ZPE and THERMAL CONTRIBUTIONS

Eelectronic	[kJ/mol]	= -549101.88
Ezpe	[kJ/mol]	= +193.751
Eelectronic+Ezpe	[kJ/mol]	= -548908.129
Eelectronic	[hartrees]	= -209.141832
Ezpe	[hartrees]	= +0.073796
Eelectronic+Ezpe	[hartrees]	= -209.068036
Thermal Correction to Energy	[kJ/mol]	= +204.067
Thermal Correction to Enthalpy	[kJ/mol]	= +206.545
Thermal Correction to Gibbs	[kJ/mol]	= +125.801

#85 O-ethenyl-hydroxylamine

Charge = 0 Multiplicity = 1 Stoichiometry C2H5NO

OPTIMIZED GEOMETRY

Center Number	Atomic Number	Atomic Type	Coordinates (Angstroms) X	Y	Z
1	6	0	-0.609356	0.364178	-0.000000
2	6	0	-1.860130	-0.075200	0.000000
3	7	0	1.700433	0.227774	0.000000
4	8	0	0.454225	-0.494558	-0.000001
5	1	0	-2.095461	-1.129695	0.000001
6	1	0	-2.670980	0.635756	0.000000
7	1	0	2.187744	-0.143420	-0.811885
8	1	0	2.187742	-0.143419	0.811887
9	1	0	-0.328959	1.408955	-0.000001

A, B, C / GHz 44.5110312 4.4938650 4.1712904

```
-------------------- FREQUENCIES AND ROTATIONAL CONSTANTS ---------------------
-------------------------------------------------------------------------------
INDEX NO. DOF TYPE  CM-1(UNSCALED)  CM-1(SCALED BY ) SYMMETRY IR-INTENSITY
   1        vib          59.10          0.00              A        5.2525
   2        vib         223.44          0.00              A       45.8597
   3        vib         345.17          0.00              A        3.8969
   4        vib         550.43          0.00              A        3.9704
   5        vib         695.83          0.00              A        6.3031
   6        vib         850.83          0.00              A       57.5419
   7        vib         866.98          0.00              A        7.9262
   8        vib         989.57          0.00              A       49.9241
   9        vib         996.67          0.00              A       20.4491
  10        vib        1141.11          0.00              A       64.2882
  11        vib        1271.24          0.00              A      143.7806
  12        vib        1317.88          0.00              A        3.5018
  13        vib        1327.86          0.00              A        1.5394
  14        vib        1424.90          0.00              A        7.8375
  15        vib        1657.65          0.00              A       21.0955
  16        vib        1697.04          0.00              A      155.9041
  17        vib        3161.62          0.00              A        1.6310
  18        vib        3185.44          0.00              A        5.2834
  19        vib        3254.38          0.00              A        5.3587
  20        vib        3436.52          0.00              A        0.2065
  21        vib        3517.67          0.00              A        1.9937
  22        rot      1.4847282          -
  23        rot      0.1498992          -
  24        rot      0.1391393          -
-------------------------------------------------------------------------------

-------------------------------------------------------------------------------
------------------------ ZPE and THERMAL CONTRIBUTIONS ------------------------
-------------------------------------------------------------------------------
Eelectronic                    [kJ/mol]   = -549234.759
Ezpe                           [kJ/mol]   = +191.231
Eelectronic+Ezpe               [kJ/mol]   = -549043.528

Eelectronic                    [hartrees] = -209.192443
Ezpe                           [hartrees] = +0.072836
Eelectronic+Ezpe               [hartrees] = -209.119607

Thermal Correction to Energy   [kJ/mol]   = +204.650
Thermal Correction to Enthalpy [kJ/mol]   = +207.128
Thermal Correction to Gibbs    [kJ/mol]   = +118.859
-------------------------------------------------------------------------------
```

#24 aziridine N-oxide Cs

```
Charge = 0 Multiplicity = 1  Stoichiometry    C2H5NO
```

OPTIMIZED GEOMETRY

Center Number	Atomic Number	Atomic Type	X	Y	Z
1	6	0	-0.235967	-0.836187	0.745505
2	6	0	-0.235967	-0.836187	-0.745505
3	7	0	-0.235967	0.461677	-0.000000
4	8	0	0.815001	1.256235	-0.000000
5	1	0	-1.141358	-1.098465	1.275632
6	1	0	0.699372	-0.984314	1.261034
7	1	0	0.699372	-0.984314	-1.261034
8	1	0	-1.141358	-1.098465	-1.275632
9	1	0	-1.152670	0.918182	-0.000000

Coordinates (Angstroms)

A, B, C / GHz 17.8799931 7.4661638 6.3913741

FREQUENCIES AND ROTATIONAL CONSTANTS

INDEX NO.	DOF TYPE	CM-1(UNSCALED)	CM-1(SCALED BY)	SYMMETRY	IR-INTENSITY
1	vib	409.32	0.00	A"	5.5343
2	vib	426.21	0.00	A'	14.8347
3	vib	678.94	0.00	A"	23.3611
4	vib	754.95	0.00	A'	2.6062
5	vib	818.51	0.00	A'	1.3925
6	vib	919.28	0.00	A"	1.0005
7	vib	1020.34	0.00	A'	55.0306
8	vib	1099.91	0.00	A"	4.6432
9	vib	1101.77	0.00	A'	65.3571
10	vib	1128.46	0.00	A"	3.3502
11	vib	1154.38	0.00	A'	45.1983
12	vib	1207.59	0.00	A"	2.2558
13	vib	1227.21	0.00	A'	10.6216
14	vib	1431.23	0.00	A'	7.2299
15	vib	1454.96	0.00	A"	5.0312
16	vib	1482.55	0.00	A'	2.8218
17	vib	3131.97	0.00	A"	8.3453
18	vib	3136.49	0.00	A'	6.4124
19	vib	3242.61	0.00	A"	1.3381
20	vib	3254.27	0.00	A'	0.0312
21	vib	3309.80	0.00	A'	22.5509
22	vib	409.32	0.00	A"	5.5343
23	vib	426.21	0.00	A'	14.8347
24	vib	678.94	0.00	A"	23.3611
25	vib	754.95	0.00	A'	2.6062
26	vib	818.51	0.00	A'	1.3925
27	vib	919.28	0.00	A"	1.0005

```
  28        vib        1020.34          0.00               A'        55.0306
  29        vib        1099.91          0.00               A"         4.6432
  30        vib        1101.77          0.00               A'        65.3571
  31        vib        1128.46          0.00               A"         3.3502
  32        vib        1154.38          0.00               A'        45.1983
  33        vib        1207.59          0.00               A"         2.2558
  34        vib        1227.21          0.00               A'        10.6216
  35        vib        1431.23          0.00               A'         7.2299
  36        vib        1454.96          0.00               A"         5.0312
  37        vib        1482.55          0.00               A'         2.8218
  38        vib        3131.97          0.00               A"         8.3453
  39        vib        3136.49          0.00               A'         6.4124
  40        vib        3242.61          0.00               A"         1.3381
  41        vib        3254.27          0.00               A'         0.0312
  42        vib        3309.80          0.00               A'        22.5509
  43        rot      0.5964124          -
  44        rot      0.2490444          -
  45        rot      0.2131933          -
--------------------------------------------------------------------------------

--------------------------------------------------------------------------------
------------------------ ZPE and THERMAL CONTRIBUTIONS -------------------------
--------------------------------------------------------------------------------
Eelectronic                    [kJ/mol]   = -549101.877
Ezpe                           [kJ/mol]   = +193.738
Eelectronic+Ezpe               [kJ/mol]   = -548908.139

Eelectronic                    [hartrees] = -209.141831
Ezpe                           [hartrees] = +0.073791
Eelectronic+Ezpe               [hartrees] = -209.068040

Thermal Correction to Energy   [kJ/mol]   = +204.059
Thermal Correction to Enthalpy [kJ/mol]   = +206.538
Thermal Correction to Gibbs    [kJ/mol]   = +125.788
--------------------------------------------------------------------------------
```
